# Defects vibrations engineering for enhancing interfacial thermal transport in polymer composites

**DOI:** 10.1126/sciadv.adp6516

**Published:** 2025-01-22

**Authors:** Yijie Zhou, Robert Ciarla, Artittaya Boonkird, Saqlain Raza, Thanh Nguyen, Jiawei Zhou, Naresh C. Osti, Eugene Mamontov, Zhang Jiang, Xiaobing Zuo, Jeewan Ranasinghe, Weiguo Hu, Brendan Scott, Jihua Chen, Dale K. Hensley, Shengxi Huang, Jun Liu, Mingda Li, Yanfei Xu

**Affiliations:** ^1^ Department of Mechanical and Industrial Engineering, University of Massachusetts Amherst, Amherst, MA 01003, USA.; ^2^ Department of Chemical Engineering, University of Massachusetts Amherst, Amherst, MA 01003, USA.; ^3^ Department of Nuclear Science and Engineering, Massachusetts Institute of Technology, Cambridge, MA 02139, USA.; ^4^ Department of Mechanical and Aerospace Engineering, North Carolina State University, Raleigh, NC 27695, USA.; ^5^ Department of Materials Science and Engineering, Stanford University, Stanford, CA 94305, USA.; ^6^ Neutron Scattering Division, Oak Ridge National Laboratory, Oak Ridge, TN 37831, USA.; ^7^ Advanced Photon Source, Argonne National Laboratory, Argonne, IL 60439, USA.; ^8^ Department of Electrical and Computer Engineering, Rice University, Houston, TX 77005, USA.; ^9^ Department of Polymer Science and Engineering, University of Massachusetts Amherst, Amherst, MA 01003, USA.; ^10^ Center for Nanophase Materials Sciences, Oak Ridge National Laboratory, Oak Ridge, TN 37831, USA.; ^11^ Rice Advanced Materials Institute, Rice University, Houston, TX 77005, USA.

## Abstract

To push upper boundaries of thermal conductivity in polymer composites, understanding of thermal transport mechanisms is crucial. Despite extensive simulations, systematic experimental investigation on thermal transport in polymer composites is limited. To better understand thermal transport processes, we design polymer composites with perfect fillers (graphite) and defective fillers (graphite oxide), using polyvinyl alcohol (PVA) as a matrix model. Measured thermal conductivities of ~1.38 ± 0.22 W m^−1^ K^−1^ in PVA/defective filler composites is higher than those of ~0.86 ± 0.21 W m^−1^ K^−1^ in PVA/perfect filler composites, while measured thermal conductivities in defective fillers are lower than those of perfect fillers. We identify how thermal transport occurs across heterogeneous interfaces. Thermal transport measurements, neutron scattering, quantum mechanical modeling, and molecular dynamics simulations reveal that vibrational coupling between PVA and defective fillers at PVA/filler interfaces enhances thermal conductivity, suggesting that defects in polymer composites improve thermal transport by promoting this vibrational coupling.

## Teaser

Contrary to common belief, defects enhance thermal transport in composites, as both the experiment and model reveal.

## INTRODUCTION

Polymer-based electronic devices, such as high-power batteries and soft robotics, have seen remarkable advancements in recent decades (*1*–*6*). However, these devices often generate a substantial amount of waste heat during operation, which can lead to overheating and safety hazards (*7*, *8*). Efficiently dissipating heat generated in polymer-based electronic devices is essential for ensuring their reliable and safe operation, but it remains a long-standing challenge (*9*, *10*). Thermally conductive polymers are needed because thermally insulating polymers hinder heat dissipation (*11*). Unfortunately, common polymers are thermal insulators with low thermal conductivities on the order of 0.1 to 0.3 W m^−1^ K^−1^ (*9*, *12*). To increase thermal conductivities (*k*) in polymers, highly thermally conductive fillers (e.g., carbon nanotube, graphene, and graphite; *k* > 1000 W m^−1^ K^−1^) have been added into polymers at high volume fractions (>40 vol %) (*13*–*15*). However, measured thermal conductivity enhancement in these composites are generally limited to within one order of magnitude, which are much lower than theoretical predicted values (*16*, *17*). Achieving polymer composites with enhanced thermal conductivity while using lower filler volume fractions would be a difficult but extremely desirable goal.

Major challenges to achieving high effective thermal conductivities in polymer composites include high filler/filler and polymer/filler interfacial thermal resistances, a lack of effective control of the dispersion of thermally conductive fillers, and/or the requirement for filler loading at high volume fractions (*18*, *19*). High filler loadings in polymers deteriorate the mechanical performance and introduce processing challenges related to polymer’s rheological behaviors (*20*). Theoretical models, numerical simulations, and experimental studies have shown that improving electron transmission and/or phonon transmission are essential for enhancing interfacial thermal transport between two crystalline solids (*9*, *21*, *22*). However, these interfacial thermal transport theories and simulations cannot be applied directly to polymers because polymer chains lack the regular periodicities and long-range orders that are characteristics of crystalline materials (*9*, *23*–*25*). Both simulations and experiments have shown that an increase in interfacial defects can lead to either a decrease (*22*, *26*, *27*) or an increase (*22*, *28*, *29*) in thermal conductance in various types of inorganic hybrid interfaces. To date, interfacial thermal transport in solids is not fully understood (*18*). It remains difficult to predict and control interfacial thermal transport behaviors in polymer composites. The systematic experimental investigations of interfacial thermal transport mechanisms in polymer composites are limited compared to inorganic materials (*22*, *30*). Understanding interfacial thermal transport mechanisms in polymer composites is challenging (*31*). This is partially due to the complex structures in polymer composites, which are typically composed of multiple phases, disorders, and have high degrees of heterogeneities at multiple length scales, ranging from the molecular scale to the microscale.

**Fig. 1.**
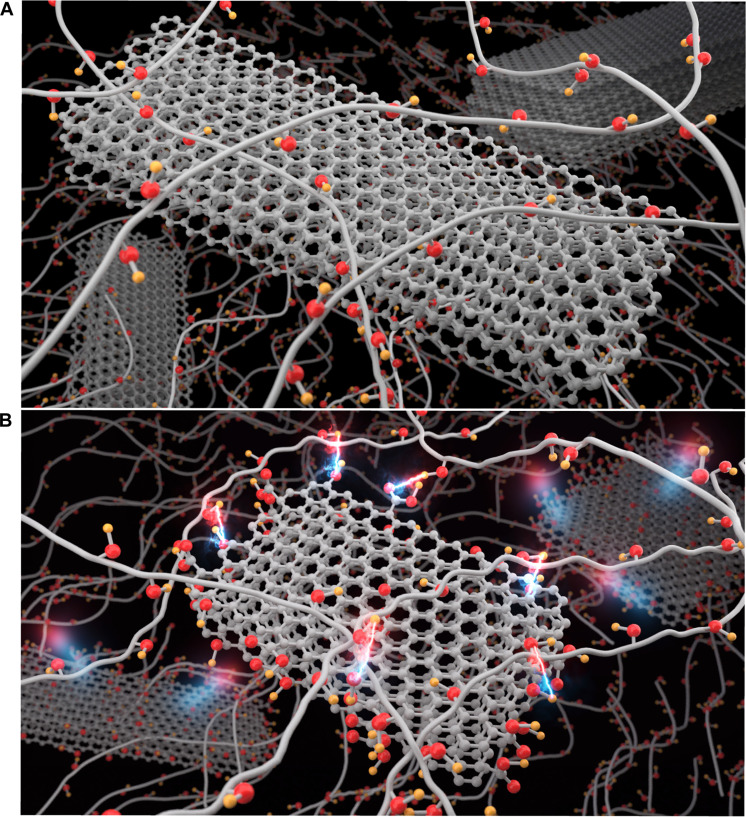
Study design for exploring defect-driven thermal transport enhancement in polymer composites. (**A**) Schematic illustration of PVA/perfect filler (graphite) composites. The polymer matrix is oxygen-containing polar PVA. The matrix is filled with high-purity graphite fillers, which are referred to as perfect fillers. (**B**) Schematic illustration of PVA/defective filler (graphite oxide) composites. The same polymer matrix made of oxygen-containing PVA, but it is filled with defective fillers (graphite oxide). To create these defective fillers, oxygen-containing defects (e.g., hydroxyl functional groups) are introduced on the surfaces and edges of the graphite particles via graphite oxidation methods (*34*). As a result, the surfaces and edges of defective graphite fillers become rough and uneven. When these defective graphite fillers are added to the polymer matrix, they create a heterogeneous composite material with heterogeneous interfaces. The defects in fillers may enhance interfacial thermal transport in polymer matrices.

In this study, to lay the foundation for understanding thermal transport mechanisms in polymer composites and controlling heat transfer across heterogenous interfaces, we address two fundamental questions: First, because defects are ubiquitous in polymer composites, can defects in fillers reduce polymer/filler interfacial thermal resistance and improve filler dispersion in polymers? Second, contradicting conventional understanding (*32*, *33*), can effective thermal conductivities in composites made of polymers and defective fillers with low thermal conductivities be higher than that of composites made of polymers and perfect fillers (graphite) with high thermal conductivities? To answer above questions, we design and synthesize perfect fillers (graphite) and defective fillers (graphite oxide) with controlled defects; we choose oxygen-containing polar polyvinyl alcohol (PVA) as a polymer matrix model for polymer composites (Fig. 1). By having the interfacial oxygen-containing defects to couple with the polymers through the vibrational resonant couplings, we experimentally measured that high 402% increase of thermal conductivity is observed with as low as 5% volume fraction of fillers. We further develop a simple quantum mechanical model that could qualitatively explain the contradictory phenomena that we observe—increasing thermal conductivity despite decreasing heat capacity as defect density increases, which is possibly due to the vibrational levels of the defect states. This simple quantum mechanical model further clarifies that composites consisting of polymers and defective fillers, characterized by low thermal conductivities, can demonstrate superior effective thermal conductivities compared to composites composed of polymers and “ideal” (or perfect) fillers such as graphite, which has high thermal conductivities. Our experimental evidence on the thermal transport properties of PVA/defective filler (graphite oxide) composites and PVA/perfect filler (graphite) composites, combined with neutron scattering, quantum mechanical modeling, and molecular dynamics simulations, suggests that defects can reduce interfacial thermal resistance and enhance effective thermal conductivity through vibrational coupling. Stronger vibrational coupling between the PVA and defective filler (graphite oxide) at PVA/filler interface introduced by defects leads to lower interfacial resistance and higher effective thermal conductivity compared to those of PVA/perfect filler (graphite) composites, as further supported by effective medium theory, quantum mechanical models, and molecular dynamics simulations. The reduction in interfacial thermal resistance and the improvement in measured effective thermal conductivity may result from the emergence of unique vibrational modes that arise from atomic defects at the filler/polymer interfaces, combined with strong intermolecular noncovalent interactions, such as hydrogen bonds. This research may open exciting opportunities to design and create polymer composites as effective thermal interface materials with high thermal conductivities. Polymer-based thermal interface materials with high thermal conductivity are crucial components in various devices, including electronics, where they play a key role in transferring heat from devices to the environment.

## RESULTS

To experimentally evaluate how defects vibrations enhance polymer/filler interfacial thermal transport through the emergence of unique vibrational modes intrinsic to the interfaces and defect atoms, we synthesize defective filler (graphite oxide) by introducing oxygen-containing defects (e.g., hydroxyl groups, epoxide groups, and carboxyl groups) on graphite surfaces and edges via graphite oxidation by a modified Hummers method (Fig. 1B) (*34*). The presence of these oxygen-containing functional groups on graphite surfaces has been confirmed by the attenuated total reflectance–Fourier transform infrared spectroscopy (ATR-FTIR) technique, which will be discussed in more detail below. A statistical analysis of lateral sizes ($a_{1}$), thicknesses ($a_{3}$), and aspect ratios $\left(\frac{a_{3}}{a_{1}}\right)$ in perfect fillers (graphite, 384 pieces) and defective fillers (graphite oxide, 570 pieces) was conducted using Zygo’s three-dimensional optical profiler (Zygo Nexview) (figs. S1 and S2). The detailed working principle of Zygo’s optical profiler is in section S1 in the Supplementary Materials. The lateral size and thickness of these fillers were confirmed through atomic force microscopy (AFM; fig. S3). The scanning electron microscope (SEM) images further corroborated the lateral size of the fillers (fig. S4).

**Fig. 2.**
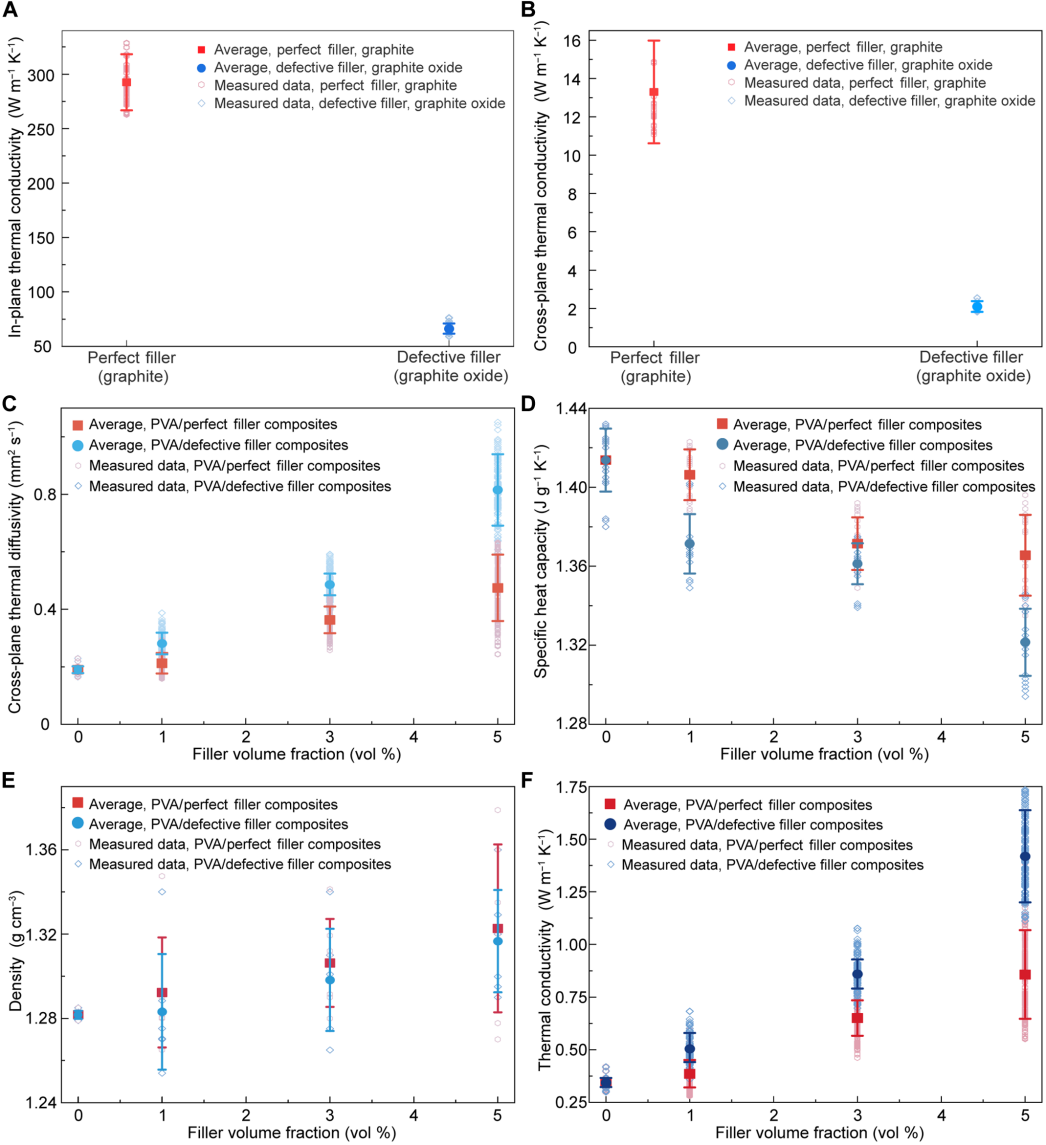
Experimental understanding of thermal transport mechanisms in fillers, polymers, and polymer composites. (**A**) Measured in-plane thermal conductivities of the “perfect fillers” (graphite) and the defective fillers (graphite oxide) at 25°C. We performed three thermal diffusivity measurements for each sample, and we examined nine different samples from separate batches. The error bars of the thermal conductivities of the fillers are based on error propagation from cross-plane thermal diffusivity, specific heat capacity, and density measurements, which were obtained from 15 different samples across five different batches. (**B**) Measured cross-plane thermal conductivities of perfect fillers (graphite) and defective fillers (graphite oxide) measured at 25°C. We performed three thermal diffusivity measurements for each sample, and we examined 12 different samples from separate five batches. The error bars of thermal conductivities of fillers are the error propagations based on cross-plane thermal diffusivity, specific heat capacity, and density measurements of fillers. (**C**) Measured cross-plane thermal diffusivities at 25°C. The error bars of thermal diffusivity measurements mainly come from thickness differences of the polymer composites and thermal diffusivities differences among nine measurements for each sample. (**D**) Measured specific heat capacities at 25°C. The error bars of specific heat capacities are the population SD based on three measurements of each sample. (**E**) Measured densities at 25°C. The error bars of the density are the population SD based on six measurements of each sample. (**F**) Measured cross-plane thermal conductivities at 25°C. The error bars of thermal conductivities are calculated on the basis of error propagations of cross-plane thermal diffusivity, specific heat capacity, and density measurements of PVA composites. Please refer to the section S1 for detailed estimation of the error propagations of measured thermal conductivity.

To confirm the presence of defects in fillers, Raman spectra of defective fillers (graphite oxide) and perfect fillers (graphite) are probed. A strong intensity of G band ~1582 cm^−1^ that originates from the $E_{2g}$ vibration mode is observed in perfect fillers (graphite) (*35*–*38*). In contrast, a decreased intensity of G band ~1586 cm^−1^ and an increased intensity of disorder-induced D band ~1350 cm^−1^ are observed in defective fillers (graphite oxide) (fig. S5) (*35*–*38*). This increased intensity of D band, related to the $A_{1g}$ breathing mode, is observed in defective fillers (graphite oxide) because the oxidation of graphite alters the basal plane structure of graphite (*35*–*38*). Defective fillers (graphite oxide) have low in-plane thermal conductivities of ~66.29 ± 4.64 W m^−1^ K^−1^ and low cross-plane thermal conductivities of ~2.10 ± 0.28 W m^−1^ K^−1^ (Fig. 2, A and B). In contrast, perfect fillers (graphite) have high in-plane thermal conductivities of ~292.55 ± 25.72 W m^−1^ K^−1^ and high cross-plane thermal conductivities of ~13.30 ± 2.68 W m^−1^ K^−1^ (Fig. 2, A and B). The error analysis of measured thermal transport properties was estimated using eqs. S1 to S5. The measured in-plane thermal conductivities in perfect fillers (graphite) are lower than those reported in a single crystal graphite (*14*, *39*). This might be due to the small crystallites in perfect fillers (graphite) and the presence of grain boundaries and defects (*14*, *39*). Measured values for thermal diffusivities (*40*) (figs. S6, A and B; S7; and S8 to S11), specific heat capacities (*41*) (fig. S6C), and densities (fig. S6D) in perfect fillers (graphite) and defective fillers (graphite oxide) are provided in the Supplementary Materials. Details for making pressed pellets of fillers [perfect fillers (graphite) or defective fillers (graphite oxide)] for thermal diffusivity measurements in fillers are in section S1.

To gain insight into thermal transport in polymer composites, thermal conductivities in polymer composites are determined in Eq. 1. We probe the thermal diffusivity (α) by a laser-flash technique and the specific heat capacity ($c_{p}$) by a differential scanning calorimetry technique. Density (ρ) is calculated by using mass divided by volume (Fig. 2)

$$k=\alpha c_{p}\rho$$ (1)

where k is thermal conductivity (W m^−1^ K^−1^), α is thermal diffusivity (m^2^ s^−1^), $c_{p}$ is specific heat capacity (J kg^−1^ K^−1^), and ρ is density (kg m^−3^).

Quantifying cross-plane thermal diffusivities and thermal conductivities is important not only for understanding thermal transport properties of polymer composites but also for optimizing performance for applications where efficient heat dissipation perpendicular to the surface is critical (*42*, *43*). The measured cross-plane thermal diffusivities in pure PVA films at room temperature are low, with a range of ~0.19 ± 0.02 mm^2^ s^−1^ (Fig. 2C). In contrast, the measured cross-plane thermal diffusivities in PVA/perfect filler (graphite, 5 vol %) composites are higher, with a range of ~0.47 ± 0.12 mm^2^ s^−1^. The cross-plane thermal diffusivities measured in PVA/defective filler (graphite oxide, 5 vol %) composites are even higher, with a range of ~0.80 ± 0.12 mm^2^ s^−1^ (Fig. 2C and figs. S12 for pure PVA films and S13 to S18 for PVA/filler composites). Measured specific heat capacities in composites are relatively similar, with values ranging from ~1.32 ± 0.017 to ~1.36 ± 0.020 J g^−1^ K^−1^ (Fig. 2D). The densities of PVA composites were measured (Fig. 2E).

**Fig. 3.**
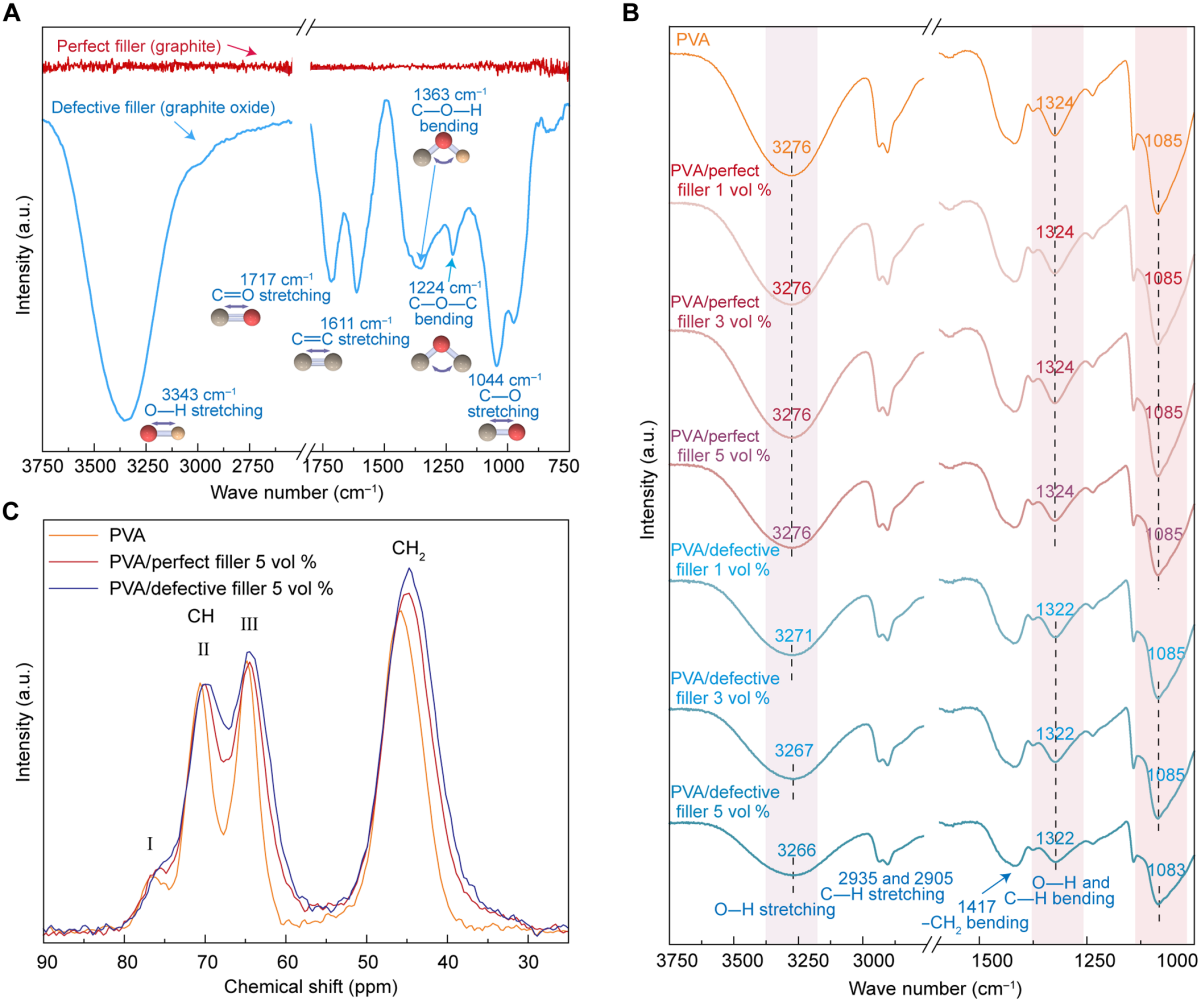
Experimental investigations of molecular-level structures and functional group vibrations in fillers, polymers, and composites. (**A**) ATR-FTIR spectra of perfect fillers (graphite) and defective fillers (graphite oxide). (**B**) ATR-FTIR spectra of PVA matrices, PVA/perfect filler (graphite) composites, and PVA/defective filler (graphite oxide) composites. (**C**) ^13^C solid-state NMR spectra of PVA matrices, PVA/perfect filler (graphite, 5 vol %) composites, and PVA/defective filler (graphite, 5 vol %) composites. a.u., arbitrary unit.

**Fig. 4.**
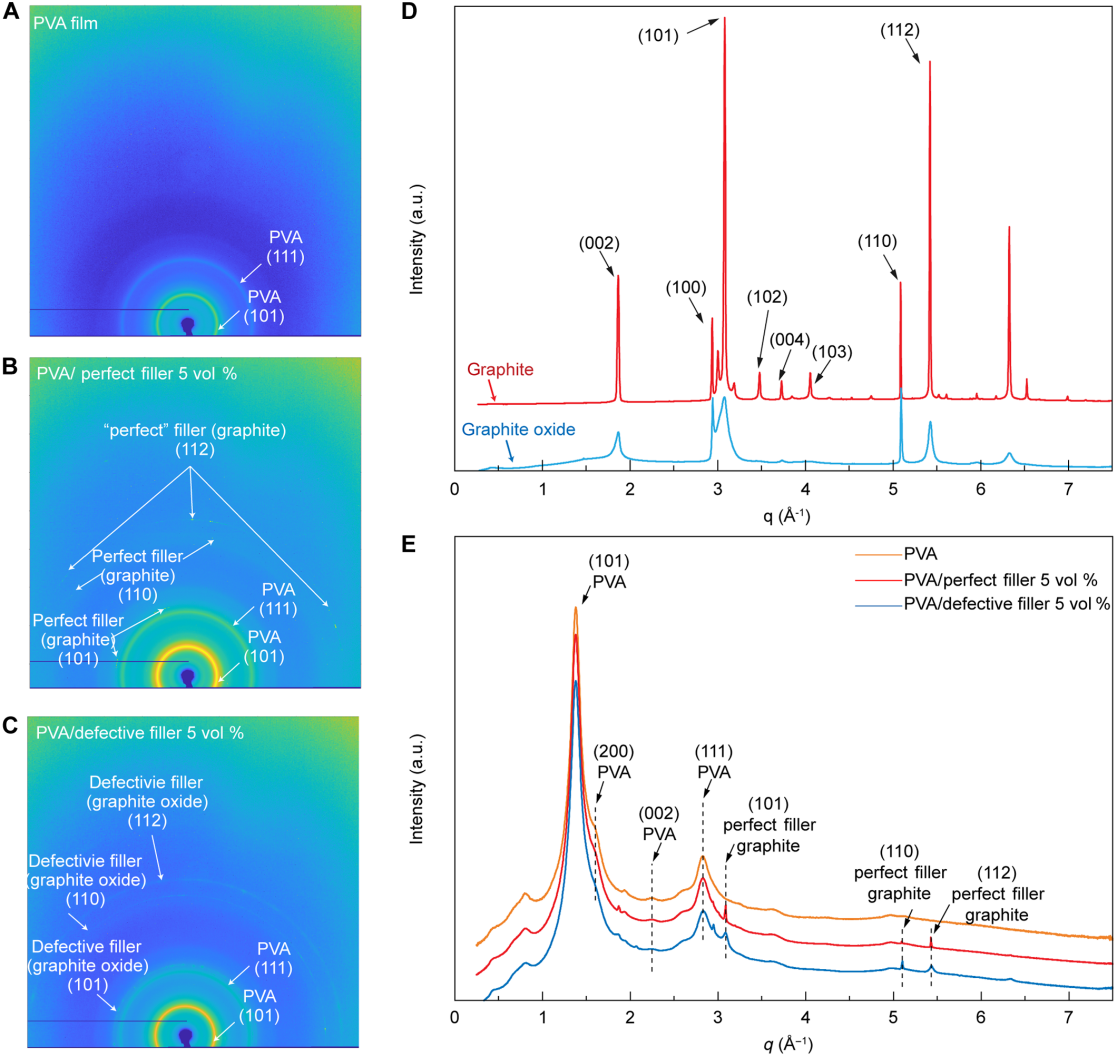
Structural study of fillers and polymer composites using a synchrotron x-ray scattering method. (**A**) Wide-angle x-ray scattering (WAXS) patterns of PVA matrices. (**B**) WAXS patterns of PVA/perfect filler (graphite, 5 vol %) composites. (**C**) WAXS patterns of PVA/defective filler (graphite oxide, 5 vol %) composites. (**D** and **E**) One-dimensional curves of the synchrotron x-ray scattering intensity versus scattering vector (*q*) for several materials used in this study: a pressed pellet of the perfect fillers (graphite), a pressed pellet of defective fillers (graphite oxide), PVA film, PVA/perfect filler (graphite, 5 vol %) composite films, and PVA/defective filler (graphite oxide, 5 vol %) composite films. (D) One-dimensional curves of the synchrotron x-ray scattering intensity versus scattering vector (*q*) for a pressed pellet of the perfect fillers (graphite) and a pressed pellet of the perfect fillers (graphite) and a pressed pellet of defective fillers (graphite oxide). (E) One-dimensional curves of the synchrotron x-ray scattering intensity versus scattering vector (*q*) for PVA films, PVA/perfect filler (graphite, 5 vol %) composites, and PVA/defective filler (graphite oxide, 5 vol %) composites. The preparation details for making samples for synchrotron x-ray scattering measurements are in the section S1.

**Fig. 5.**
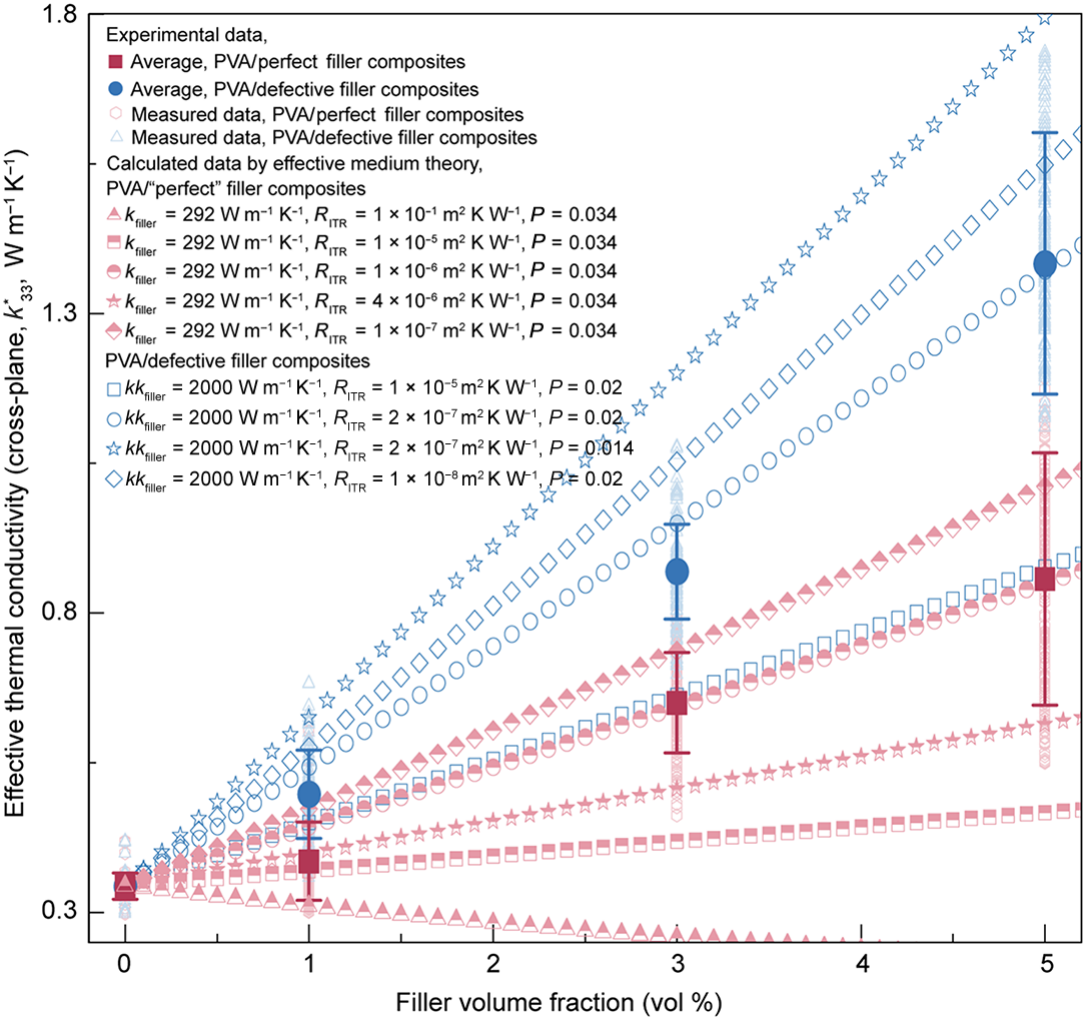
Comparisons between effective medium theory model and experiment in PVA/filler composites in the cross-plane direction. Additional calculation details are available in the section S2. The comparison between measured and calculated thermal conductivities in the cross-plane direction ($k_{33}^{*}$) in PVA/defective filler (graphite oxide) composites, using variable $R_{\text{ITR}}$, indicates an overestimated $R_{\text{ITR}}$ in PVA/defective filler (graphite oxide, 5 vol %) composites, approximately at 2 × 10^−7^ m^2^ K W^−1^. The comparison between measured and calculated thermal conductivities in the cross-plane direction ($k_{33}^{*}$) in PVA/perfect filler (graphite) composites, using variables $R_{\text{ITR}}$, indicates an underestimated $R_{\text{ITR}}$ in PVA/perfect filler (graphite, 5 vol %) composites, approximately at 1 × 10^−6^ m^2^ K W^−1^. The overestimated $R_{\text{ITR}}\;\left(2\times 10^{-7}\;m^{2}\;K\;W^{-1}\right)$ in PVA/defective filler (graphite oxide, 5 vol %) composites is still smaller than $R_{\text{ITR}}\;\left(1\times 10^{-6}\;m^{2}\;K\;W^{-1}\right)$ in PVA/perfect filler (graphite) composites.

**Fig. 6.**
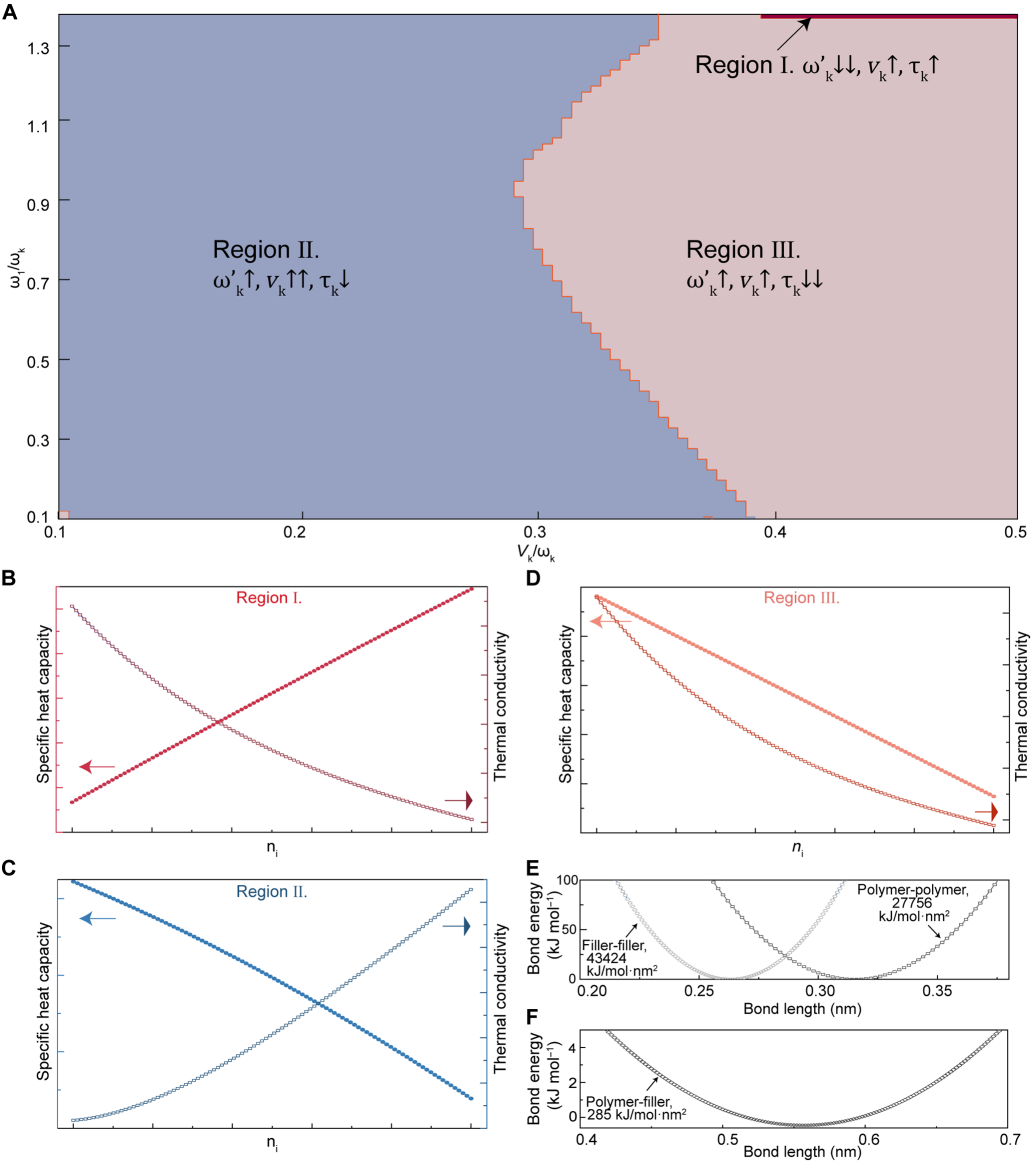
Theoretical study of thermal transport mechanisms in polymer composites. (**A**) Numerical results of the coupled vibration state model of polymer composite. The results are correlated to the vibrational energy of the fillers ($\omega_{1}$), vibrational energy of the polymer backbone ($\omega_{k}$), and the coupling potential between fillers and polymer backbone ($V_{k}$). The regions I, II, and III indicates the different behavior of thermal conductivity κ(T) and specific heat capacity C(T) with the increasing filler fraction ($n_{i}$) of the polymer composites. The double arrows in regions II and III indicate the domaining effect in specific region. The renormalized phonon energy of polymer backbone ($\omega_{k}^{\prime }$), group velocity ($v_{k}$), and phonon relaxation time ($\tau_{k}$) are interpreted for explaining the behavior of the changing C(T) and κ(T). (**B**) In region I, with the increasing filler fraction, C(T) increases, while κ(T) decreases. (**C**) In region II, the increasing filler fraction results in decreased C(T) and increased κ(T), which matches the results observed in PVA/defective filler (graphite oxide) composites. (**D**) In region III, both C(T) and κ(T) decrease as the increasing of filler fraction. (**E** and **F**) Relationships between coarse-grain potential to bond lengths in various materials (*65*). (E) Polymer-polymer (PVA-PVA) and filler-filler (graphite oxide-graphite oxide). (F) Polymer-filler (PVA-graphite oxide).

**Fig. 7.**
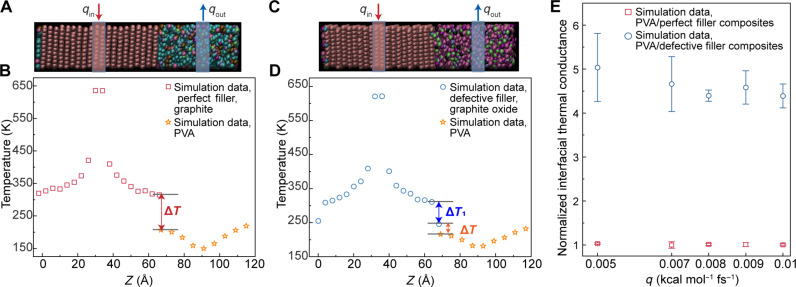
Molecular dynamics simulations of the thermal transport in polymer composites. (**A**) Visual molecular dynamics (VMD) snapshot of PVA/perfect filler (graphite) composites. (**B**) Temperature profile (*q* = 0.009 kcal mol^−1^ fs^−1^) showing the temperature drop (Δ*T*) at the graphite/PVA interface in PVA/perfect filler (graphite) composites. (**C**) VMD snapshot of PVA/defective filler (graphite oxide) composites. (**D**) Temperature profile (*q* = 0.009 kcal mol^−1^ fs^−1^) showing the temperature drop (Δ*T*) at the graphite-oxide-PVA interface and the temperature drop (Δ*T*_1_) between the graphite layer connected with hydroxyl (−OH) groups and its adjacent layer in PVA/defective filler (graphite oxide) composites. (**E**) Normalized interfacial thermal conductance at polymer-filler interfaces in PVA/perfect filler (graphite) composites and PVA/defective filler (graphite oxide) composites. Error bars represent the sample SD.

**Fig. 8.**
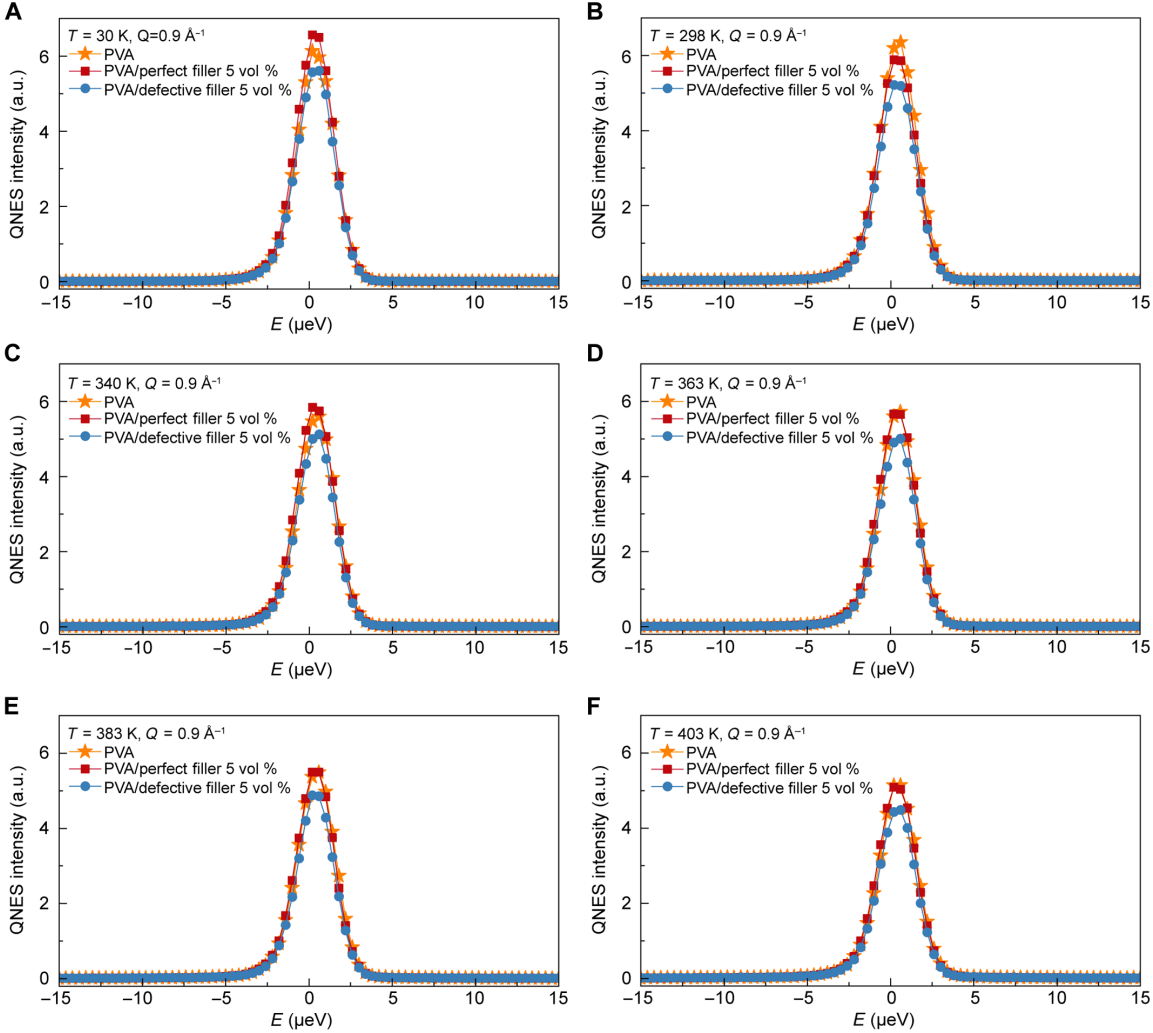
The analysis of polymer chain dynamics in composites through neutron scattering experiments. Comparison of representative QENS spectra of pure PVA, PVA/perfect filler (graphite, 5 vol %) composites, and PVA/defective filler (graphite oxide, 5 vol %) composites measured at *Q* = 0.9 Å^−1^ as a function of temperature: (**A**) 30 K, (**B**) 298 K, (**C**) 340 K, (**D**) 363 K, (**E**) 383 K, and (**F**) 403 K. The spectra have been zoomed in to magnify the elastic line centered at *E* = 0.

Notably, measured cross-plane thermal conductivities (measured effective thermal conductivity) in polymer/defective filler (graphite oxide, 5 vol %) composites and PVA/perfect filler (graphite, 5 vol %) composites are ~1.38 ± 0.22 and ~0.86 ± 0.21 W m^−1^ K^−1^, respectively (Fig. 2F). However, measured thermal conductivities in defective fillers (graphite oxide) are lower than those of perfect fillers (graphite) (Fig. 2, A and B). The x-ray scattering patterns indicate that there are no preferred polymer chain and filler orientations in these polymer composites (it will be discussed below) (*44*). This could be favorable for achieving near-isotropic thermal conductivities in PVA composites. Thus, measured cross-plane thermal conductivities in polymer composites suggest the effective thermal conductivities in near-isotropic PVA-based composites (Fig. 2F). Despite the lower thermal conductivities and specific heat capacities of the defective fillers compared to the perfect fillers (Fig. 2, A and B, and fig. S6C), the measured thermal conductivity in PVA/defective filler (graphite oxide) composites (~1.38 ± 0.22 W m^−1^ K^−1^) is higher than that in PVA/perfect filler (graphite) composites (~0.86 ± 0.21 W m^−1^ K^−1^) (Fig. 2F). In addition, the specific heat capacities in PVA/defective filler (graphite oxide) composites are lower than those in PVA/perfect filler (graphite) composites (Fig. 2D). To better understand these measured behaviors of the thermal transport properties in PVA/perfect filler (graphite) composites and PVA/defective filler (graphite oxide) composites, we conducted ATR-FTIR measurements (Fig. 3, A and B), ^13^C solid-state nuclear magnetic resonance (NMR) experiments (Fig. 3C), and x-ray scattering experiments (Fig. 4) while also applying an effective medium theory (Fig. 5), developing a simple quantum mechanical model (Fig. 6), performing molecular dynamics simulations (Fig. 7), and conducting neutron scattering experiments (Fig. 8), all of which will be discussed in more detail below.

From the atomic level view of polymer/filler interfacial thermal transport, atoms in fillers interact with atoms in polymers via noncovalent interactions between PVA and fillers (graphite or graphite oxide). To experimentally understand why the thermal conductivities in PVA/defective filler (graphite oxide) composites are higher than those of PVA/perfect filler (graphite) composites (Fig. 2F) despite the measured thermal conductivities in defective fillers being lower than those of perfect fillers (Fig. 2, A and B), we first examine the interfacial vibration couplings by investigating the hydrogen bonds between PVA and fillers using the ATR-FTIR technique (Fig. 3, A and B). To experimentally confirm the presence of intermolecular vibration couplings arising from atomic mass defects at polymer/filler interfaces, we first examine vibration modes of functional groups on defective fillers (graphite oxide) and perfect fillers (graphite) by using ATR-FTIR spectroscopy (Fig. 3A). Stretching vibration peaks of oxygen-containing groups—including 1044 cm^−1^ for alkoxy group (C─O), 1717 cm^−1^ for carbonyl group (C═O), and 3343 cm^−1^ for hydroxyl group (−OH)—are observed in the defective fillers (graphite oxide) (*45*). Bending vibration peaks, including 1224 cm^−1^ for epoxy groups (C─O─C) and 1363 cm^−1^ for hydroxyl groups (C─OH), are observed in Fig. 3A (*45*). No vibration modes of oxygen-containing groups are observed on perfect fillers (graphite) (Fig. 3A). Figure 3B shows ATR-FTIR spectra of PVA, PVA/perfect filler (graphite) composites, and PVA/defective filler (graphite oxide) composites. The peak at 3276 cm^−1^ in the ATR-FTIR spectra of PVA is attributed to O─H stretching vibration. This peak is broad, indicating that it arises from a range of different O─H bonds, including inter- or intramolecular hydrogen bonds (*46*, *47*). The addition of defective fillers (graphite oxide) to PVA matrices causes a shift in the strong vibration peak of PVA from 3271 to 3267 cm^−1^ and 3266 cm^−1^ with the addition of 1 and 5 vol % defective fillers (graphite oxide), respectively (Fig. 3B). These peak shifts suggest that there may be interactions between hydroxyl groups on PVA chains and functional groups on surfaces of defective fillers (graphite oxide) (*46*, *48*, *49*). Interactions could be due to hydrogen bonding between the hydroxyl groups on PVA and the oxygen-containing functional groups on defective fillers (graphite oxide), such as carbonyl, hydroxyl, and epoxy groups. This hydrogen bonding could result in a change in the vibrational energy of the hydroxyl groups on PVA, leading to the observed peak shifts. These hydrogen bonding interactions can cause a distortion of the electron cloud around the C─H and O─H bonds, resulting in a red shift of the corresponding vibrational modes (*46*, *48*, *49*). Similarly, interactions between hydroxyl groups on PVA chains and oxygen-containing functional groups on surfaces of defective fillers (graphite oxide) can cause a red shift of the C─O bond peak at 1085 cm^−1^. This red shift is due to the increased electron density around the C─O bond because of hydrogen bonding interactions (Fig. 3B) (*48*, *49*). The presence of the perfect fillers (graphite) has not substantially affected molecular structures of PVA because peaks observed in the PVA are still present in the composites (Fig. 3B) (*48*, *49*).

To experimentally confirm whether defects improve filler dispersions in polymers, we probe the structures of PVA chains and their interfaces with fillers using ^13^C solid-state NMR spectroscopy at room temperature (Fig. 3C) (*50*). Defective fillers (graphite oxide) and perfect fillers (graphite) are added into PVA matrices at 5 vol %. There is a tall peak at 46 parts per million (ppm) corresponding to CH_2_ carbons and a group of three peaks between 60 and 80 ppm, conventionally named peak I, II, and III from downfield to upfield, corresponding to CH carbons whose OH forms 2, 1, and 0 hydrogen bonds with neighboring protons (Fig. 3C) (*51*, *52*). Signals from perfect fillers (graphite) and defective fillers (graphite oxide) are undetectable. The chemical shift of the CH_2_ signal in PVA observed in ^13^C solid-state NMR spectrum is at 45.77 ppm. However, when PVA is incorporated with perfect fillers (graphite), the chemical shift of the CH_2_ signal changes to ~45.07 ppm; when PVA is mixed with defective fillers (graphite oxide), the chemical shift of the CH_2_ signal shift changes to ~44.76 ppm. Similarly, peak II of the CH signal in the ^13^C solid-state NMR spectroscopy of PVA is at 70.79 ppm, while it shifts to 70.27 and 69.88 ppm in PVA/perfect filler (graphite) composites and PVA/defective filler (graphite oxide) composites, respectively. These upfield (toward smaller ppm value) chemical shifts of CH_2_ and CH signals could be due to two potential mechanisms: First, the presence of the conductive electrons in graphite or graphite oxide changes the magnetic field in the surrounding, and second, the introduction of fillers into PVA matrices increases the population of gauche conformers in PVA chains.

In addition, the peak widths at half-maximum of the CH_2_ signal in PVA, PVA/perfect filler (graphite) composites, and PVA/defective filler (graphite oxide) composites are 891, 1062, and 1116 Hz, respectively (Fig. 3C). The CH_2_ signal in PVA/defective filler (graphite oxide) composite is the broadest, while that in neat PVA is the narrowest. The CH signal shows similar peak broadening: Peak II and peak III in neat PVA are resolved at around 40% peak maximum, while those in PVA composites are resolved at around 70 and 80% maximum, respectively. The peak broadening in the PVA composites again could be due to two mechanisms: First, the presence of conductive electrons in the graphite or graphite oxide increase the magnetic field inhomogeneity in their surroundings, and second, the presence of the fillers broadens the distribution of the conformation of the PVA segments in their surroundings, which leads to a broadening of the ^13^C solid-state NMR signals (*53*–*55*). Both mechanisms of the influence of the fillers to the polymer matrix are short range, i.e., within a few nanometers. Therefore, larger peak position shifts and peak widths both indicate better dispersion of the fillers in the matrix. We also investigate representative transmission electron microscopy (TEM) images of PVA/defective filler (graphite oxide) composites and PVA/perfect filler (graphite) composites (fig. S19) to provide visible images of PVA/filler composites at the microscale or nanoscale. We also probe the tapping-mode AFM images of PVA/defective filler (graphite oxide) composites and PVA/perfect filler (graphite) composites (fig. S20) to provide visible images of PVA/filler composites at the microscale or nanoscale.

To gain a better understanding of relationships between thermal transport and structures in polymers and polymer/filler composites, we used the synchrotron wide-angle x-ray scattering technique to investigate orientations of fillers and polymer chains (Fig. 4) (*56*). Our observations reveal characteristic Bragg scatterings, which include the (101) plane groups in PVA matrix and (101) plane groups in graphite-based fillers (Fig. 4, B and C, and fig. S21) (*57*, *58*). The x-ray scattering patterns also displayed multiple concentric rings, which are characteristic of Bragg scattering off different lattice planes of crystals. The presence of these concentric rings rather than distinct dots in patterns suggests that there are no preferred orientations of crystallites within polymer composites (Fig. 4, A to C) (*56*, *59*). We further calculated the statistical orientations of the fillers based on their scattering patterns at the (101) peak in PVA/perfect (graphite) composites (Fig. 4B) and PVA/defective filler (graphite oxide) composites (Fig. 4C), both yielding <cos^2^θ> values of ^1^/_3_, which further suggests that fillers are randomly oriented. Thus, the measured thermal conductivities in the cross-plane direction of polymer composites (Fig. 2F), particularly in near-isotropic PVA-based composites with randomly oriented fillers, suggest efficient heat transfer throughout all directions within the material. More detailed calculation of <cos^2^θ> is available in the fig. S21.

Summarizing the above results, we have observed unusual thermal transport phenomena in polymer composites. First, as the filler fraction ratio increases, the thermal conductivity of the polymer composite increases as expected, while the specific heat capacity decreases (Fig. 2, D and F). The thermal conductivity of the polymer composite is higher when defective fillers are used despite these fillers having lower intrinsic thermal conductivities (Fig. 2, A and F). We note that multiple factors (e.g., filler thermal conductivity, polymer thermal conductivity, polymer chain dynamics, interfacial bonding, and vibrational coupling between polymer and filler at the polymer/filler interface) can influence thermal transport in composite materials. Nonetheless, we first suspect that the interfacial bonding between the polymer matrix and the fillers is key to achieving higher thermal conductivity. Next, we explore other factors affecting thermal conductivity in polymer composites. Molecular dynamics simulations were used to investigate how vibrational coupling at the polymer/filler interface affects thermal transport in PVA/perfect filler (graphite) composites and PVA/defective filler (graphite oxide) composites. Neutron scattering experiments were conducted to study how PVA chain dynamics influence thermal transport in these composites. In addition, the radial distribution function (RDF), coordination number, and mean-squared displacement (MSD) of PVA carbons were determined using molecular dynamics simulations to assess their impacts on thermal transport.

To better understand how interfacial bonding affects measured higher thermal conductivity, we first apply an effective medium theory to evaluate the impact of various factors on the composite’s thermal conductivity (Fig. 5 and fig. S22). These factors, derived from our measurements, include filler lateral size ($a_{1}$, figs. S1 to S3), filler thickness ($a_{3}$, figs. S1 to S3), filler aspect ratio ($\frac{a_{3}}{a_{1}}$, fig. S1), statistical filler orientation (<cos^2^θ>, Fig. 4 and fig. S21), filler thermal conductivity (Fig. 2A), and polymer (PVA) thermal conductivity (Fig. 2F). To ensure accuracy, we collected statistical data for $a_{1}$ and $a_{3}$ for 570 pieces of defective fillers (graphite oxide) and for 384 pieces of perfect fillers (graphite) (figs. S1 to S3). To ensure the effective medium theory is applicable, we also analyzed the distribution of fillers in PVA matrices using TEM (fig. S19) and AFM (fig. S20), along with the ^13^C solid-state NMR technique (Fig. 3C). We have further developed a simple quantum mechanical model and molecular dynamic simulations based on the coupling of the vibrational states of the polymer chains (PVA backbone) and the fillers at PVA/filler interface in these composites. This allow us to better understand how the interfacial bonding can be altered due to the introduction of defective fillers and in turn lead to enhanced vibrational coupling between PVA and filler at PVA/filler interface, reduced interfacial thermal resistance, and improved thermal conductivity (Figs. 6 and 7 in the main text; sections S3 and S4). The effective thermal conductivities of polymer composites, calculated using effective medium theory, were determined through eqs. S6 to S16. Equations S17 to S50 relate to a simple effective quantum mechanical model aimed at better understanding how dynamic defects enhance thermal conductivity. Equation S51 pertains to molecular dynamics simulations for understanding thermal transport mechanisms in polymer composites. In addition to studying the relationship between measured thermal conductivity and vibrational coupling between PVA and the filler at the PVA/filler interface in these composites, we further investigated the influence of polymer chain dynamics on the thermal transport properties in PVA/perfect (graphite) composites and PVA/defective filler (graphite oxide) composites using neutron scattering experiments (Fig. 8), with further details provided below. Equations S52 and S53 relate to neutron scattering experiments and analyses to investigate polymer chain dynamics in polymer composites.

We compare the calculated thermal conductivities ($k_{33}^{*}$) using an effective medium theory (*60*) and the experimental data for both PVA/defective filler (graphite oxide) composites and PVA/perfect filler (graphite) composites in the cross-plane direction (Fig. 5 and fig. S22). This comparison is intended as a guideline for understanding which factor is most influential in thermal transport. In the effective medium theory, the thermal transport mainly depends on a few key material and geometrical parameters, including a thermal conductivity of the filler ($k_{\text{filler}}$), a volume fraction of fillers (f), interfacial thermal resistance ($R_{\text{ITR}}$), a polymer matrix thermal conductivity ($k_{\text{polymer}}$), and geometric characteristics of fillers including lateral dimensions ($a_{1}$and $a_{2}$; figs. S1 to S3), thickness ($a_{3}$; figs. S1 to S3), the filler’s aspect ratio ($p=\frac{a_{3}}{a_{1}}$; fig. S1), and the statistical orientation of the fillers <cos^2^θ> (*61*), as specified in eqs. S6 to S16 (*60*). In our calculation, we have used experimental data for $a_{1}$ (fig. S1), $a_{3}$ (fig. S1), p (fig. S1), $k_{\text{polymer}}$ (Fig. 2F), <cos^2^θ> (Fig. 4 and fig. S21), and f. The only unknowns are the filler’s thermal conductivity $k_{\text{filler}}$ and the interfacial thermal resistance $R_{\text{ITR}}$. To find $R_{\text{ITR}}$, we first estimate $k_{\text{filler}}$ and then vary $R_{\text{ITR}}$ to match the experimental data. For PVA/defective filler (graphite oxide) composites, we have taken $k_{\text{filler}}$ to be 2000 W m^−1^ K^−1^ (*62*, *63*). This is considerably higher than the measured value (66 W m^−1^ K^−1^) for defective fillers (graphite oxide) (Fig. 2A) (*62*, *63*). Under this assumption, the obtained $R_{\text{ITR}}$ will be overestimated PVA/defective filler (graphite oxide) composites. In such a case, this yields an overestimated $R_{\text{ITR}}$ value, which is estimated to be approximately ~2 × 10^−7^ m^2^ K W^−1^ in PVA/defective filler (graphite oxide, 5 vol %) composites (Fig. 5) (*60*, *64*). On the other hand, for PVA/perfect filler (graphite) composites, we have taken $k_{\text{filler}}$ to be 293 W m^−1^ K^−1^, which is the measured thermal conductivity of pressed pellet of the perfect fillers (graphite) and is an underestimation of the intrinsic thermal conductivity of the filler (Fig. 2A). Under this assumption, the obtained $R_{\text{ITR}}$ will be underestimated in PVA/perfect filler (graphite) composites. In such a case, this yields an underestimated $R_{\text{ITR}}$ value, which is estimated to be approximately 1 × 10^−6^ m^2^ K W^−1^ in PVA/perfect filler (graphite, 5 vol %) composites (Fig. 5). While in both cases we have taken conservative estimates, the overestimated $R_{\text{ITR}}$ (~2 × 10^−7^ m^2^ K W^−1^) in PVA/defective filler (graphite oxide, 5 vol %) composites is still smaller than the underestimated $R_{\text{ITR}}$ (1 × 10^−6^ m^2^ K W^−1^) in PVA/perfect filler (graphite, 5 vol %) composites. This underscores the importance of interfacial bonding in reducing the interfacial thermal resistances and enhancing thermal transport in polymer composites.

To gain a deeper insight on the reduced interfacial thermal resistance caused by defective fillers, we develop a simple quantum mechanical model to explain this counter-intuitive thermal transport phenomenon based on the vibrational coupling picture. Despite the simplicity, the model can explain the distinct behaviors of thermal conductivity and heat capacity, at least qualitatively. The effective Hamiltonian of this coupled system can be written as Eq. 2

$$\begin{array}{cc} H & =\sum_{q}\omega_{q}\left(a_{q}^{+}a_{q}+\frac{1}{2}\right)+\omega_{1}\left(a_{1}^{+}a_{1}+\frac{1}{2}\right)+\omega_{2}\left(a_{2}^{+}a_{2}+\frac{1}{2}\right)\\ & +\sum_{q\;j=1,2}\left(V_{q\;j}a_{q}^{+}a_{j}+V_{q\;j}^{*}a_{j}^{+}a_{q}\right) \end{array}$$ (2)

where $\omega_{1}$, $\omega_{q}$, and $V_{q\;j}$ denote the phonon vibrational energy of the fillers, the polymer backbone phonon spectra, and the coupling energy potential between fillers and polymer backbone, respectively. To keep the essence without introducing too many undeterminable parameters, we adopt a minimalist approach to focus on a single-mode phonon. The coupling potentials $V_{q\;j}$ can be estimated on the basis of molecular dynamics simulation results from literature data, as shown in Fig. 6 (E and F) (*65*). The standard Bosonic commutation relation hold for both polymer backbone and fillers, i.e., $\left[a_{q},a_{k}^{+}\right]=\delta_{\text{qk}},\left[a_{i},a_{j}^{+}\right]=\delta_{\mathrm{ij}}$ etc. For complex defective fillers, an additional energy level $\omega_{2}$ is introduced. This model is inspired by Anderson’s two-level system model of thermal transport in disordered solids, where the vibrational coupling becomes dominant (*66*), and has been used to explain complex thermal transport process in interacting defects systems (*67*). The closed form solution of self-energy, group velocity, and thermal conductivity can be derived from Green’s function equation of motion approach (section S3). The numerical result of the simple quantum mechanical model is shown in Fig. 6. The details for specific heat capacity calculation are eq. S47. In regions II and III (Fig. 6A), the decrease of the specific heat capacity C(T) with increasing filler fraction ($n_{i}$) links to the increasing of the renormalized vibrational energy ($\omega_{k}^{\prime }$) (Fig. 6, C and D). The increasing of thermal conductivity κ(T) in region II is a result of higher increasing of phonon group velocity ($v_{k}$) compared to the phonon scattering ($\tau_{k}$). This trend generally occurs when the coupling potential $V_{k}$ is weaker than $\omega_{k}$, while $\omega_{1}$ may be smaller or comparable to $\omega_{k}$. On the other hand, if the increasing of phonon scattering dominates the enhancement of the group velocity, the thermal conductivity will decrease with $n_{i}$ (region III). Last but not least, it is also worthwhile mentioning that the vibrational coupling is not the most commonly used coupling for defects; for more common potential coupling of defects, the behaviors are not expected to emerge. Given the extreme structural complexity of the polymer composite, we believe the model offers an alternative pathway to demonstrate the possibility of the counterintuitive results between κ(T) and C(T) in a qualitative manner within reasonable parameter range, rather than focusing on exact parameters in the simplified model (*68*).

It is found that measured thermal transport properties in composites including PVA/perfect filler (graphite) composites and PVA/defective filler (graphite oxide) composites (Fig. 2, D and F) located in modeled region II (Fig. 6, A and C), where the increased fraction of filler leads to the increased thermal conductivities and decreased specific heat capacity of the composites. On the basis of the simple quantum mechanical model, it is anticipated that with the increased fraction of fillers, increase in thermal conductivity of the polymer composite can be observed due to increased phonon group velocity in region II (Fig. 6A) (*65*).

Last, for defective fillers (graphite oxide), which can be modeled as nondegenerate defects with $\omega_{1}\neq \omega_{2}$, will lead to a higher thermal conductivity κ(T) but lower heat capacity C(T) compared to the perfect filler (modeled as $\omega_{1}=\omega_{2}$), as discussed in section S3. These simple mechanical model results agree with experimental observations: PVA/defective filler (graphite oxide) composites made of polymers and defective fillers with low thermal conductivities can exhibit higher effective thermal conductivities than those in PVA/perfect filler (graphite) made of polymers and perfect fillers such as graphite, which have high thermal conductivities. Stronger vibrational coupling between the polymer and defective filler at the polymer/filler interface in PVA/defective filler (graphite oxide) composites may reduce interfacial thermal resistance and enhance thermal conductivity compared to PVA/perfect filler (graphite) composites.

We further understood how vibrational coupling between the polymer and filler at the polymer/filler interfaces leads to lower interfacial thermal resistance and higher effective thermal conductivity in PVA/defective filler (graphite oxide) composites than in PVA/perfect filler (graphite) composites using molecular dynamic simulation techniques at the atomic level (Fig. 7, fig. S23, and section S4). We calculated the density of states for the carbon atoms near the PVA/filler interface region, with filler carbons (graphite or graphite oxide) and PVA carbons considered separately. The results shown in fig. S23 (A and B) for the 0- to 25-THz frequency range suggest that sharper modes are available for graphite carbons near the PVA/filler interface in the PVA/perfect filler (graphite) composite compared to those in the PVA/defective filler (graphite oxide) composite. However, unlike the sharper modes in the PVA/perfect filler (graphite) composite (fig. S23A), the broader modes of graphite oxide carbons near the PVA/filler interface in the PVA/defective filler (graphite oxide) composite (fig. S23B) allow for stronger coupling between the filler carbons and PVA carbons in the PVA/defective (graphite oxide) filler composite compared to the PVA/perfect filler (graphite) composite. Moreover, the results in fig. S23 (C and D) suggest that PVA carbons near the PVA/filler interface in the PVA/defective filler (graphite oxide) composite exhibit more modes at low frequencies compared to those in the PVA/perfect filler (graphite) composite. These factors contribute to stronger vibrational coupling between the PVA carbons and filler carbons at the PVA/filler interface in the PVA/defective filler (graphite oxide) composite, leading to a greater interfacial thermal conductance (Fig. 7E) and lower interfacial thermal resistance in the PVA/defective filler (graphite oxide) composite.

As shown in Fig. 7 (B and D), the temperature plots representing the temperature in different regions along the *z* direction for PVA/perfect filler (graphite) composites and PVA/defective filler (graphite oxide) composites are presented. Visual molecular dynamics (VMD) snapshots of PVA/perfect filler (graphite) composites and PVA/defective filler (graphite oxide) composites are shown in Fig. 7 (A and C) (*69*). We observed that Δ*T* (temperature difference at the PVA/filler interface) was much smaller for PVA/defective filler (graphite oxide) composites compared to PVA/perfect filler (graphite) composites (Fig. 7, B and D). This supports lower interfacial thermal resistance and higher interfacial thermal conductance in PVA/defective filler (graphite oxide) composites compared to PVA/perfect filler (graphite) composites. To understand the higher interfacial thermal conductance in PVA/defective filler (graphite oxide) composites despite fewer PVA chains near the surface, we calculated the MSD (fig. S24) of PVA carbons near the PVA/filler interface. MSD is higher in PVA/defective filler (graphite oxide) composites than in PVA/perfect filler (graphite) composites, suggesting more freedom of movement for PVA chains, which facilitates heat transfer and results in greater interfacial thermal conductance and lower thermal resistance.

In PVA/defective filler (graphite oxide) composites, the top and bottom layers of the defective filler (graphite oxide layers) are graphite layers that are connected to functional groups such as hydroxyl groups. We observed a notable temperature drop for these layers of graphite with respect to their adjacent layers (marked as Δ*T*_1_ in Fig. 7D). The temperature drop is attributed to the increased roughness of the graphite layers with hydroxyl groups. The calculated roughness of the top graphite layer in PVA/defective filler (graphite oxide) composites compared to PVA/perfect filler (graphite) composites, as shown in fig. S24, reveals that the roughness in PVA/defective filler (graphite oxide) composites is an order of magnitude higher. This higher roughness, supported by coordination numbers (fig. S24, C and D) and visualized in fig. S24A, aligns with our neutron scattering results, which will be discussed in more detail below.

To further understand how polymer chain (PVA backbones) dynamics influence the effective thermal conductivity in PVA/perfect filler (graphite) composites and PVA/defective filler (graphite oxide) composites (Fig. 2, D and F) at the atomic level, we use the quasi-elastic neutron scattering (QENS) technique. Figure 8 compares representative QENS spectra of PVA, PVA/perfect filler (graphite, 5 vol %) composites, and PVA/ defective filler (graphite oxide, 5 vol %) composites measured at *Q* = 0.9 Å^−1^ across temperatures of 30, 298, 340, 363, 383, and 403 K. These temperatures (30, 298, 340, 363, 383, and 403 K) cover the range below and above the glass transition temperature of PVA, as determined by differential scanning calorimetry (fig. S25). Further details on the QENS measurements are available in the Supplementary Materials (section S5).

The QENS spectra at 30 K were taken as the instrument resolution function (Fig. 8A). At this temperature, every chemical species in a sample remains “dynamically frozen” and immobile within the instrument’s sensitivity. Because the masses of each sample were nearly the same, at 30 K, the elastic intensity centered at *E* = 0 scales with the number of incoherent scatterers, which are the hydrogen atoms in the PVA sample. An increase in temperature enhances thermally induced dynamics, resulting in a monotonic decrease in the elastic intensity. While the PVA and PVA/perfect filler (graphite, 5 vol %) composites are more comparable in intensity, with some temperature-dependent variations, the PVA/defective filler (graphite oxide, 5 vol %) composites systematically exhibits lower intensity (Fig. 8, B to F). This is also the case for the 30 K dataset (Fig. 8A), where practically all the scattering intensities are elastic. The number of incoherent scatterers (hydrogen atoms in the PVA polymer) is lower in the PVA/defective filler (graphite oxide, 5 vol %) composites compared to the PVA/perfect filler (graphite, 5 vol %) composites despite both composites containing 5 vol % of fillers. Furthermore, QENS data from PVA at 298 K (Fig. 8B) could be fitted with a delta function, confirming that there are no detectable dynamics at 298 K in PVA. However, the QENS data from PVA/perfect filler (graphite, 5 vol %) composites and PVA/defective filler (graphite oxide, 5 vol %) composites (Fig. 8B) needed an additional function, a Cole-Cole function with α=0, to fit the spectra. The half width at half maximum (HWHMs) of the QENS signals are *Q* independent, suggesting a completely localized dynamic process within the PVA molecules in the presence of fillers. The data for all three samples at other temperatures (Fig. 8, C to F) were also fitted with the same function and showed the same *Q*-independent behavior.

Elastic incoherent scattering factor (EISF) obtained from PVA, PVA/perfect filler (graphite, 5 vol %) composites, and PVA/defective filler (graphite oxide, 5 vol %) composites—as presented in fig. S26—are similar between the samples at all temperatures. At all the measured temperatures, including the baseline temperature of 30 K, the elastic intensity from PVA/perfect filler (graphite, 5 vol %) composites is always systematically higher compared to that from PVA/defective filler (graphite oxide, 5 vol %) composites. This suggests that the PVA/defective filler (graphite oxide, 5 vol %) composites must have fewer PVA polymer chains despite having the same volume fraction of the fillers. Because 5% of the graphite and graphite oxide volume fraction is used to prepare both composites, fewer PVA must be filling up the interfacial region with graphite oxide compared to the interfacial region with graphite, likely due to the rougher surface for the PVA/filler interface in the PVA/defective filler (graphite oxide, 5 vol %) composites. The anticipated arrangement of PVA molecules around the filler particles is schematically presented in fig. S27. When the particle surface is rougher, there are more void spaces around the graphite oxide filler, which are not accessible for polymer molecules in the composite. Thus, the intensities of the QENS signal suggest that the graphite oxide filler surface must have higher roughness than the graphite filler surface. The observed neutron scattering results in fig. S27 align with the molecular dynamics simulations of the root mean square roughness, showing that the roughness of the top graphite layer in graphite oxide is higher than that of graphite (fig. S24A).

The reduced filling of PVA chains in the interfacial region with graphite oxide, compared to graphite, and the increased roughness of the graphite oxide surface, as indicated by QENS (Fig. 8), EISF (fig. S26), and molecular dynamics simulations of coordination number and roughness (fig. S24, A, C, and D), may not fully account for the lower interfacial thermal resistance observed in PVA/defective filler (graphite oxide, 5 vol %) composites compared to PVA/perfect filler (graphite, 5 vol %) composites. This suggests that the enhanced vibrational coupling between PVA and the defective filler at the PVA/defective filler interface plays a key role in achieving higher thermal conductivity and lower interfacial thermal resistance, relative to PVA/perfect filler (graphite, 5 vol %) composites.

Moreover, the RDF (fig. S24B) shows a higher magnitude for PVA carbons in the PVA/perfect filler (graphite) composite, indicating that more carbon atoms in the PVA backbone are closer to the graphite surface compared to the PVA/defective filler (graphite oxide) composite. This further supports the conclusion that enhanced vibrational coupling at the PVA/defective filler interface is important for achieving higher thermal conductivity and lower interfacial thermal resistance compared to PVA/perfect filler (graphite, 5 vol %) composites.

## DISCUSSION

This work presents experimental evidence that challenges traditional understandings of interfacial defects as adding resistance to heat transfer across heterogenous interfaces. Instead, we demonstrate that interfacial defects can enhance effective thermal conductivities in polymer composites through vibrational couplings that arise from oxygen-containing defects. Specifically, measured cross-plane thermal conductivities ~1.38 ± 0.22 W m^−1^ K^−1^ in PVA/defective filler (graphite oxide) composites (graphite oxide, 5 vol %) are higher than those ~0.86 ± 0.21 W m^−1^ K^−1^ in PVA/perfect filler (graphite, 5 vol %). However, measured in-plane thermal conductivities in defective fillers (~66.29 ± 4.64 W m^−1^ K^−1^) are lower than those of perfect fillers (~292.55 ± 25.72 W m^−1^ K^−1^). Interfacial defects enhance thermal transport across heterogenous interfaces in polymer composites. Our measured thermal transport properties, neutron scattering experimental observations, and numerical result well explained the observed opposite trend between decreasing specific heat capacity and increasing thermal conductivity in polymer composites.

By understanding and controlling interfacial thermal transport, it is possible to reduce interfacial thermal resistances in polymer composites with high densities of heterogenous interfaces. Through manipulating the local heat flux carried by atomic vibrations that comprise these interfacial vibration couplings, effective thermal conductivities of polymer composites can be enhanced. By developing polymer composites with high cross-plane thermal conductivities, it is possible to create effective thermal interface materials that can be used in various sustainable applications such as thermal management of electronic devices, aerospace materials, and energy storage.

## MATERIALS AND METHODS

### Experimental design

To experimentally and theoretically investigate how defect vibrations enhance thermal transport at the polymer/filler interface in polymer composites through unique vibrational modes linked to these interfaces and defect atoms, we synthesized defective filler (graphite oxide) by introducing oxygen-containing defects on the surfaces and edges of graphite using a modified Hummers method for graphite oxidation.

### Materials

All chemicals were purchased and used as received. PVA (weight-average molecular weight of 89,000 to 98,000, 99 + % hydrolyzed, CAS: 9002-89-5), potassium permanganate (CAS: 7722-64-7), and graphite (flakes, ≥98% carbon basis, +50 mesh particle size (≥80%), natural, CAS: 7782-42-5) were purchased from Sigma-Aldrich. Concentrated sulfuric acid (CAS: 7664-93-9) was purchased from Alfa Aesar. Sodium nitrate (CAS: 7631-99-4), hydrogen peroxide (CAS: 7722-84-1), and ethanol (CAS: 64-17-5) were purchased from Thermo Fisher Scientific.

### Preparation of defective fillers (graphite oxide)

Defective fillers (graphite oxide) with oxygen-containing defects (oxygen and hydroxyl functional groups) on graphite surfaces and edges were prepared via the modified Hummers method (*34*). Typically, graphite flakes (3.0 g) were mixed with concentrated sulfuric acid (69.0 ml) and sodium nitrate (1.5 g) in a 500-ml flask at 0°C with continuous stirring for 30 min. After that, potassium permanganate (9.0 g) was added into a reaction mixture in small portions to prevent the temperature from rising above 10°C. Then, the temperature of the reaction mixture was raised to 35°C using an oil bath, and the mixture was stirred for 30 min. After completion of the reaction, 138 ml of deionized water was gradually added into the reaction. Then, the temperature of the reaction mixture was raised to ~98°C using an oil bath, and the mixture was stirred for 15 min. The suspension was reacted further by adding a mixture of hydrogen peroxide (6 ml) and water (23 ml). The mixture was stirred for 1 hour. After the graphite oxide was formed through the procedure described above, it was separated from the suspension using a centrifuge. The centrifugation was carried out at a speed of 10,000 rpm for 10 min. The separated graphite oxide was washed with water using an ultrasonic bath for 8 hours. The next step involved centrifuging graphite oxide multiple times and washing it with water until the pH of the supernatant (the liquid above the solid material) reached 7. After the purifying process, graphite oxide was dried in an oven at a temperature of 110°C for 24 hours before it was further used in the experiment. We used a modified Hummer’s method. Notably, unlike the hydrogen peroxide (H_2_O_2_):sulfuric acid (H_2_SO_4_):permanganate (KMnO_4_):graphite (mole ratio mole ratio 2.15: 5.15: 0.23: 1) used in the original reference for Hummer’s method (*34*), we used a different ratio of hydrogen peroxide (H_2_O_2_):sulfuric acid (H_2_SO_4_):permanganate (KMnO_4_):graphite (mole ratio of 3.07:5.15:0.23:1). In this work, extra hydrogen peroxide was used as a terminating agent for any residual potassium permanganate (*34*, *70*). This contrasts with the original reference (*34*), where a lesser amount of hydrogen peroxide was used. The C/O atomic ratio of graphite oxide was estimated from (*34*, *71*–*74*).

### Preparation of PVA solution and PVA thin films

A solution containing 8 weight % (wt %) of PVA was prepared using deionized water as the solvent. Typically, PVA powders (8 g) were added into deionized water (92 g). Then, the temperature of the mixture was raised to ~85°C using an oil bath, and the mixture was stirred for 3 hours until the PVA was dissolved. The solution was then allowed to cool to room temperature and was stirred for an additional hour. The PVA films (thickness of ~20 μm) for structural and thermal transport property studies were prepared by casting PVA solution using a coating rod (Buschman HS75) at room temperature. The PVA films were dried at 95°C for 15 min by a hot plate to remove the solvent. PVA films were lastly dried in an oven at a temperature of 95°C for 12 hours before they were further used in the experiment.

### Preparation of PVA/defective filler (graphite oxide) composite films or PVA/perfect filler (graphite) composite films

The procedure involved dispersing the fillers (graphite oxide or graphite) in PVA solution using ultrasonication and constant stirring to obtain a homogeneous dispersion. This step was repeated three times to ensure complete dispersion of the fillers in the PVA solution. Once the dispersion was prepared, it was cast onto a clean substrate using a coating rod (Buschman HS75) to achieve a uniform film under room temperature. The film was then dried using a hot plate at 95°C for 5 min to remove the excess water and further dried in an oven at 95°C for 12 hours to ensure complete removal of the water and to form a solid composite film. The perfect filler composite preparation follows the same procedure as the defective filler composite preparation, except for the type of filler material used. For example, to make composite films made of PVA/defective filler (graphite oxide, 5 vol %) composites, graphite oxide powders (0.033 g) were added into PVA solution (8 wt %, 5 ml). This solution was dispersed using an ultrasonic bath for 30 min. After ultrasound treatment, the dispersion was followed up with constant stirring for 8 hours. This procedure of ultrasonication and constant stirring was repeated three times in total. These steps were necessary to ensure that fillers were dispersed in the PVA solution. The dispersion was then cast on a clean glass substrate. A coating rod (Buschman HS75) was used to make a uniform film. The film was first dried on a hot plate at 95°C for 5 min and then dried at 95°C for 12 hours in an oven.

## Supplementary Materials

### This PDF file includes:

Supplementary Text Figs. S1 to S27 Table S1 References
