# Supplementary material for: Defects vibrations engineering for enhancing interfacial thermal transport in polymer composites
Source: Sci Adv. 2025 Jan 22;11(4):eadp6516. doi: 10.1126/sciadv.adp6516 (PMC11753404; doi:10.1126/sciadv.adp6516)
Supplement: Supplementary file 1 — Supplementary Text Figs. S1 to S27 Table S1 References [file sciadv.adp6516_sm.pdf]

Science Advances
AAAS

Supplementary Materials for

Defects vibrations engineering for enhancing interfacial thermal transport in polymer composites

Yijie Zhou *et al.*

Corresponding author: Yanfei Xu, yanfeixu@umass.edu

Sci. Adv. **11**, eadp6516 (2025)
DOI: 10.1126/sciadv.adp6516

This PDF file includes:

Supplementary Text
Figs. S1 to S27
Table S1
References

Supplementary Text

Section S1. Experimental characterizations, sample preparations, and error analysis.

1.1 Experimental characterizations and sample preparations.

Attenuated total reflectance Fourier transform infrared spectroscopy (ATR-FTIR) analysis. The ATR-FTIR was carried out on a Bruker ALPHA II FTIR system. The ATR-FTIR measurements were performed by scanning the spectrum from 4000 cm^{-1} to 400 cm^{-1}, with a resolution of 2 cm^{-1}. Each sample was scanned 16 times to improve the signal-to-noise ratio, and the average value was taken. Before the ATR-FTIR measurements, samples were dried using an oven at 110 °C for 12 hours to remove any moisture. The ATR-FTIR measurements were taken at room temperature under ambient atmosphere. Measured data was corrected using automatic baseline correction.

Raman spectroscopy analysis. The Raman spectra were recorded on a Horiba LabRAM Raman spectrometer. The Raman spectrometer was equipped with a 600/mm grating and a 50× objective. The acquisition time was 30 s. The laser power on the sample was kept at 10 mW. The excitation laser wavelength was 532.5 nm. In each Raman measurement, two accumulations were carried out. Thus, each spectrum was averaged three measurements. Raman measurements were taken at room temperature under ambient atmosphere.

^{13}C solid-state nuclear magnetic resonance (NMR) analysis. ^{13}C solid-state NMR cross polarization with magic angle spinning (CP/MAS) experiments were performed on a Bruker 600 MHz solid-state NMR spectrometer in a 4 mm broadband-observe CP/MAS probe. The spinning speed was 8 kHz. A contact time of 2 ms, a recycle delay of 20 s, and a decoupling field strength of 70 kHz were used for NMR experiments. Chemical shift was calibrated by setting the unprotonated aromatic carbon signal of 1,4-di(*t*-butyl)benzene at 148.8 ppm.

Synchrotron X-ray scattering measurement. Synchrotron X-ray scattering experiments were conducted at beamline 12-ID-B of the Advanced Photon Source located at Argonne National Laboratory. The wavelength λ of the X-ray beam is 0.93 Å (13.3 keV). The sample-to-detector distance was 188.389 mm. Thin polymer composite films were folded to achieve a thickness above 0.25 mm for synchrotron X-ray scattering experiments. To make a pressed pellet of fillers (the "perfect" fillers (graphite) or defective fillers (graphite oxide)) for synchrotron X-ray scattering measurements, the weight of fillers being used was between 0.15 to 0.45 grams, and they were poured into a customized mold with a diameter of 25.4 millimeters. "Perfect" filler (graphite) was purchased from Sigma-Aldrich (size of 50 mesh, CAS: 7782-42-5); defective filler (graphite oxide) was prepared via the modified Hummers method (*34*). A hydraulic press was used to apply pressure to the graphite or graphite oxide flakes that were poured into the customized mold, resulting in the formation of pressed pellets of fillers ("perfect" fillers (graphite) or defective fillers (graphite oxide)). Typically, after applying a pressure of 58 MPa using a hydraulic press for 5 minutes, the pressed pellets of fillers ("perfect" fillers (graphite) or defective fillers (graphite oxide)) were removed from the mold.

Thermal diffusivity measurements. Cross-plane thermal diffusivities in PVA and PVA composites were measured by laser flash apparatus with a lamp voltage of 150 V and a pulse width of 50 μs (LFA 467 HyperFlash, NETZSCH). All PVA films and polymer composites were sprayed with graphite (DGF 123, Miracle Power Products) prior to measuring cross-plane thermal diffusivities. A transparent model provided by the NETZSCH software was used for analyzing the data obtained from laser flash experiments and extracting thermal diffusivity. The densities of pressed pellets of fillers (the "perfect" fillers (graphite) or defective fillers (graphite oxide))can be calculated by measuring the thickness and weight of a pressed pellets of fillers ("perfect" fillers (graphite) or

defective fillers (graphite oxide)) made from the flakes. The in-plane and cross-plane thermal diffusivities of the pressed pellets of fillers (the “perfect” fillers (graphite) or defective fillers (graphite oxide)) are measured using a lamp voltage of 250 V and a pulse width of 600 μs. The penetration model provided by the NETZSCH software was used for analyzing the data obtained from laser flash experiments and extracting the cross-plane thermal diffusivities of a pressed pellet of fillers (the “perfect” fillers (graphite) or defective fillers (graphite oxide)). The in-plane anisotropic model provided by the NETZSCH software was used for extracting the in-plane thermal diffusivities of a pressed pellet of fillers (the “perfect” fillers (graphite) or defective fillers (graphite oxide)).

Preparation of pressed pellets of fillers (“perfect” fillers (graphite) or defective fillers (graphite oxide)) for thermal diffusivity measurements in “perfect” fillers (graphite) and defective fillers (graphite oxide). To measure the cross-plane and in-plane thermal diffusivities of “perfect” fillers (graphite) purchased from Sigma-Aldrich (flakes, ≥98% carbon basis, +50 mesh particle size (≥80%), natural, CAS: 7782-42-5, Sigma-Aldrich product number 808113-2.5KG) and defective fillers (graphite oxide) made by modified Hummers’ method (*34*), samples were prepared using the following procedures. Dried fillers (0.25 g) were first dispersed in deionized water (50 mL) to make 5 g/L dispersion. The dispersion was then ultrasonicated for 30 minutes using an ultrasonic cleaner (VWR Symphony 97043-936), followed by constant stirring for 8 hours at room temperature. This process of ultrasonication (30 minutes) and stirring (8 hours) was repeated three times in total. After the ultrasonication and stirring process, the dispersion was dried in an oven (Thermo Scientific Blue M vacuum oven VO914A1) at 110 °C for 24 hours. After drying, the ultrasound-treated fillers were weighed out (around 0.15 to 0.45 g) as per experimental design and were poured into a customized mold with a diameter of 25.4 mm, to make pressed pellets of fillers (“perfect” fillers (graphite) or defective fillers (graphite oxide)) for thermal diffusivity testing. A pressure of 58 MPa was applied to the mold using a hydraulic press for 5 minutes to form the pressed pellets of fillers (“perfect” fillers (graphite) or defective fillers (graphite oxide)). For the in-plane thermal diffusivity tests, the pressed pellets of fillers (“perfect” fillers (graphite) or defective fillers (graphite oxide)) was carefully removed from the pellet pressing mold and then loaded into the in-plane thermal diffusivity test holder with (or without) graphite spray (DGF 123, Miracle Power Products).For the cross-plane thermal diffusivity tests, the pressed pellets of fillers (“perfect” fillers (graphite) or defective fillers (graphite oxide)) was further cut into a smaller pellet with a diameter of 12.7 mm using a hollow punch of the same diameter and then loaded into the cross-plane thermal diffusivity test holder with graphite spray (DGF 123, Miracle Power Products).

Preparation of polymer films and polymer composite films for thermal diffusivity measurements. To measure the cross-plane thermal diffusivities, PVA films, PVA/defective filler (graphite oxide) composites, and PVA/“perfect” filler (graphite) composites with thicknesses of ~15-30 μm were cut into a round-shape films (12.7 mm in diameter) by using a hollow punch with a diameter of 12.7 mm. The films were first sprayed with graphite coatings (DGF 123, Miracle Power Products) on both sides and were then kept at room temperature for 5 minutes until the graphite spray dried. The details for making PVA films, PVA/defective filler (graphite oxide) composites, and PVA/“perfect” filler (graphite) composites were in the main text (experimental section).

Specific heat capacity measurements. The specific heat capacities of samples were measured using differential scanning calorimetry (DSC, TA Instruments 2500). In DSC measurements, samples were heated at a ramp rate of 10 °C/minute until it reached a temperature of 130 °C. Then it was cooled down to 0 °C at the same ramp rate of 10 °C/minute. DSC measurements involved repeating the heating and cooling cycle four times, with the first cycle used to eliminate any thermal history of the sample and the last three cycles used to obtain the specific heat capacity of the sample. Sample weight in DSC experiments was in the range of 8 to 12 mg. During the DSC measurement, nitrogen was used as a DSC standard purge gas and was kept purging with a gas flow of 400 mL/ minute.

Scanning electron microscope measurements (SEM). The SEM images of the fillers were taken by FEI Magellan 400 XHR Scanning Electron Microscope.

Three-dimensional (3D) profiling of "perfect" fillers (graphite) and defective fillers (graphite oxide): sample preparations and characterizations. Lateral and thickness characterization of "perfect" fillers (graphite) and defective fillers (graphite oxide) via a Zygo's three-dimensional optical profiler and atomic force microscopy (AFM). The lateral dimensions ($a_1$and a_2) and thickness (a_3) of these fillers (Figs. S1-S3) were measured utilizing a Zygo's three-dimensional optical profiler (Zygo Nexview) and atomic force microscopy (Asylum MFP-3D). The Zygo's three-dimensional optical profiler uses the principle of optical interference to measure a sample (*75, 76*). The working principle of the Zygo's three-dimensional optical profiler involves dividing the illumination into two paths: one directed towards a precision reference surface, and the other towards the test surface (*75, 76*). The reflections from these surfaces converge at a camera detector, where they interfere with each other, forming a pattern of light and dark intensities. This interference pattern accurately depicts the surface topography with lateral and thickness information (a_1, a_2, a_3 in Fig. S2B, Fig. S2C, Fig S2Eand Fig S2F in supplementary materials) of the test surface (*75, 76*).

Sample preparations for 3D profiling of fillers. To measure the lateral dimensions ($a_1$and a_2) and thickness (a_3) of "perfect" fillers (graphite) and defective fillers (graphite oxide) (Fig. S2) using a Zygo's three-dimensional optical profiler and atomic force microscopy, fillers on silica substrates are prepared. The fillers were first dispersed in deionized water to make a 1 g/L dispersion. The dispersion was then dropped onto a clean silica substrate and heated on a hot plate at 95 °C for 10 minutes. This step was done to evaporate the water and ensure that the fillers were evenly distributed on the substrates.

Transmission electron microscopy (TEM) measurements of PVA composites: sample preparations and characterizations. TEM characterizations. TEM images of PVA composites were captured using a JEOL NeoARM scanning transmission electron microscope.

Sample preparations for TEM characterizations. The TEM samples were prepared by dropping 20 μL of PVA/"perfect" filler (graphite, 5 vol%) composites dispersion or PVA/defective filler (graphite oxide, 5 vol%) composites dispersion onto the tabbed, center-marked grid (400 mesh, copper). Deionized water was the solvent for the dispersion. To achieve a sample thin enough for TEM imaging (less than 100 nm in thickness), these images were obtained by applying a dilute solution (1 wt%) of polymer while maintaining the same 5 vol% filler/PVA ratio as used in the

samples for thermal transport measurements. To make thin film samples on the TEM grid, most of the composite dispersion dropped onto the TEM grid has to be removed by Kimwipes paper. The remaining solution was dried under normal atmospheric conditions at room temperature.

Quasielastic neutron scattering (QENS) measurements of PVA composites. The dynamics of polyvinyl alcohol (PVA) film, PVA/"perfect" filler (graphite, 5 vol%) composites, and PVA/defective filler (graphite oxide, 5 vol%) composites were analyzed using the QENS technique. A QENS experiment was performed using the backscattering silicon spectrometer (BASIS) (*77*) of the spallation neutron source of the Oak Ridge National Laboratory. BASIS was operated at the standard configuration using the incident neutrons with bandwidth centered at 6.4 Å. In this setting, employing the Si (111) analyzer panels, the instrument provides a fine resolution of 3.4 μeV (full width at half-maximum). It covers an energy transfer range of ±100 μeV and a Q (momentum transfer vector) range of 0.2–2.0 $Å^{-1}$. This setup is typically used to resolve segmental dynamics in polymeric systems (*78, 79*). All three samples of nearly equal masses, in the form of films of ~ 0.2 mm in thickness, were placed in flat plate aluminum sample holders and sealed using indium wire. Data were collected at 298 K, 340 K, 363 K, 383 K, and 403 K, covering the temperature ranges below and above the glass transition temperatures obtained from the differential scanning calorimetry (Fig. S25). Sample-specific resolution was measured at the baseline temperature of ~ 30 K. Closed-cycle refrigerators were used to control the sample temperature. For detailed information on data analysis, please refer to Section S5 in supplementary materials.

1.2 Population standard deviation and error propagation analysis of thermal diffusivity, specific heat capacity, density, and thermal conductivity.

The population standard deviation (σ) is determined by Equation S1.

$$\sigma = \sqrt{\frac{\Sigma(x_i-\mu)^2}{N}} \quad \text{(S1)}$$

Where σ is the population standard deviation, Σ is the sum from 1 to N, N is the total number of the population, x_i is an individual value (e.g., thermal diffusivity, specific heat capacity, or density), and μ is the population mean.

The population mean (μ) is determined by Equation S2.

$$\mu = \bar{x} = \frac{1}{N}\left(\sum_{i=1}^{N} x_i\right) = \frac{x_1 + x_2 + \cdots + x_N}{N} \quad \text{(S2)}$$

Where μ (or $\bar{x}$) is the population mean, Σ is the sum from 1 to N, N is the total number of the population, and x_i is an individual value (e.g., thermal diffusivity, specific heat capacity, or density).

Thermal conductivities (k) in polymer composites are determined by Equation S3(the same as Equation 1 in the main text)

$$k = \alpha C_p \, \rho \quad \text{(S3)}$$

Where k is thermal conductivity ($W\ m^{-1}\ K^{-1}$), α is thermal diffusivity ($m^2\ s^{-1}$), C_p is specific heat capacity ($J\ kg^{-1}\ K^{-1}$), and ρ is density ($kg\ m^{-3}$).

The error propagation of k is determined by Equations S4 and S5.

$$\Delta k = \sqrt{\left(\frac{\partial k}{\partial \alpha}\right)^2 (\sigma_\alpha)^2 + \left(\frac{\partial k}{C_p}\right)^2 (\sigma_{c_p})^2 + \left(\frac{\partial \rho}{\rho}\right)^2 (\sigma_\rho)^2} \quad \text{(S4)}$$

which equals to

$$\Delta k = \sqrt{\left(\bar{c_p} \times \bar{\rho}\right)^2 (\sigma_\alpha)^2 + (\bar{\alpha} \times \bar{\rho})^2 (\sigma_{c_p})^2 + \left(\bar{\alpha} \times \overline{C_p}\right)^2 (\sigma_\rho)^2} \quad \text{(S5)}$$

Where Δk is the error propagation of thermal conductivity ($W\ m^{-1}\ K^{-1}$), σ_α is the population standard deviation of thermal diffusivity ($m^2\ s^{-1}$), σ_{c_p} is the population standard deviation of the specific heat capacity ($J\ kg^{-1}\ K^{-1}$), and σ_ρ is the population standard deviation of density ($kg\ m^{-3}$).

Section S2. Calculated effective thermal conductivities of polymer composites using effective medium theory.

Our measured cross-plane thermal conductivity (measured effective thermal conductivity) of PVA/defective filler (graphite oxide) composites and PVA/"perfect" filler (graphite) composites (Fig. 2B) are compared with the calculated effective thermal conductivities in the cross-plane direction (k_{33}^*, Fig. 5) based on the effective medium theory (Equations S6-S16) (*60*).

According to Equations S6-S16, the thermal conductivity of a filler (k_{filler}), the volume fraction of fillers (f), the interfacial thermal resistance (R_{ITR}) of a polymer composite, the thermal conductivity of a polymer matrix ($k_{polymer}$), the lateral dimensions (a_1 and a_2) and thickness (a_3) of these fillers as illustrated in Figs. S1 and S2, the aspect ratio of filler ($\frac{a_3}{a_1}$) as illustrated in Fig. S1, and the statistical orientations of the fillers $< cos^2\theta >$ (Fig. S21) play crucial roles in calculating values of $k_{11}^*, k_{22}^*, and\ k_{33}^*$ (Fig. 5 and Fig. S22).

$$k_{11}^* = k_{22}^* = k_{polymer} \frac{2+f[\beta_{11}(1-L_{11})(1+<co\ ^2\theta>)+\beta_{33}(1-L_{33})(1-<co\ ^2\theta>)]}{2-f[\beta_{11}L_{11}(1+<co\ ^2\theta>)+\beta_{33}L_{33}(1-<co\ ^2\theta>)]} \quad \text{(S6)}$$

$$k_{33}^* = k_{polymer} \frac{1+f[\beta_{11}(1-L_{11})(1-<co\ ^2\theta>)+\beta_{33}(1-L_{33})<\cos^2\theta>]}{1-f[\beta_{11}L_{11}(1-<\cos^2\theta>)+\beta_{33}L_{33}<\cos^2\theta>]} \quad \text{(S7)}$$

$$\beta_{ii} = \frac{k_{ii}^c - k_{polymer}}{k_{polymer} + L_{ii}(k_{ii}^c - k_{polymer})} \quad (i = 1,2,3) \quad \text{(S8)}$$

$$k_{ii}^c = \frac{k_{polymer}}{1+\gamma L_{ii} \frac{k_{filler}}{k_{polymer}}} \quad (i = 1,2,3) \quad \text{(S9)}$$

$$L_{11} = L_{22} = \frac{p^2}{2(p^2-1)} - \frac{p}{2(p^2-1)^{\frac{3}{2}}} \cosh^{-1} p \quad (for\ p > 1) \quad \text{(S10)}$$

$$L_{11} = L_{22} = \frac{p^2}{2(p^2-1)} + \frac{p}{2(1-p^2)^{\frac{3}{2}}} \cos^{-1} p \quad (for\ p < 1) \quad \text{(S11)}$$

$$L_{33} = 1 - 2L_{11} \quad \text{(S12)}$$

$$\gamma = \left(1 + \frac{1}{p}\right) \frac{R_{ITR} k_{polymer}}{a_1} \quad (for\ p \geq 1) \quad \text{(S13)}$$

$$\gamma = (1 + 2p) \frac{R_{ITR} k_{polymer}}{a_3} \quad (for\ p \leq 1) \quad \text{(S14)}$$

$$p = \frac{\alpha_3}{\alpha_1} \quad \text{(S15)}$$

$$< \cos^2\theta > = \frac{\int \rho(\theta)\cos^2\theta sin \ \ d\theta}{\int \rho(\theta)\cos^2\theta \ d\theta} \text{ (S16)}$$

Where $k_{11}^{*} and \ \ k_{22}^{*}$ represent the in-plane thermal conductivities, while k_{33}^{*} represents cross-plane thermal conductivity in a polymer composite (Fig. S1). The k_{ii}^{*}, k_{filler}, and $k_{polymer}$ denote calculated effective thermal conductivity of the polymer composite, theoretical thermal conductivity of the filler, and theoretical thermal conductivity of the polymer. The subnote ii indicates the direction, where 11 and 22 represent the in-plane directions, and 33 represents the cross-plane direction in this study (Fig. S1). f is the volume fraction of the fillers. p represents the aspect ratio ($\frac{a_3}{a_1}$) of the filler, which is related to a_3 and a_1. Measured thickness (a_3) and lateral dimensions (a_1 and a_2) are shown in Figs. S1-S3. $< \cos^2\theta >$ represents statistical orientations of the fillers in polymer matrix. R_{ITR} represents the interfacial thermal resistance in a polymer composite.

The volume fraction of fillers (f) in this experimental research ranges from 1% to 5%. The thermal conductivities measured in the in-plane and cross-plane directions of a pressed pellet of fillers (the “perfect” fillers (graphite) or defective fillers (graphite oxide) are depicted in Figs. 2A and 2B, respectively. The thermal conductivities measured in the cross-plane directions of polymer matrices and polymer composites are shown in Fig. 2F. The lateral dimensions and thicknesses of “perfect” fillers (graphite) and defective fillers (graphite oxide) were measured using a Zygo's three-dimensional optical profiler (Figs. S1-S2) and atomic force microscopy (Fig. S3), to determine the aspect ratios of the fillers ($\frac{a_3}{a_1}$). To confirm the morphology and distribution of the fillers as measured by the optical profilometer (Figs. S1-S2), additional techniques including transmission electron microscopy (Fig. S19) and atomic force microscopy (Fig. S20) were employed.

To accurately calculate the statistical aspect ratios ($\frac{a_3}{a_1}$ in Figs. S1C and S1F) of fillers, the 3D profiles of both “perfect” fillers (graphite) and defective fillers (graphite oxide) were measured using 384 pieces of “perfect” fillers (graphite) and 570 pieces of defective fillers (graphite oxide). More details related to $\frac{a_3}{a_1}$ calculations are in Figs. S1. Based on the Gaussian fitting results, we measured the aspect ratios for “perfect” fillers (graphite) to be centered at 0.034 (ranging from 0.004 to 0.1 in Fig. S1F), while the aspect ratios for the defective filler (graphite oxide) centered at 0.02 (ranging from 0.001 to 0.065 in Fig. S1C in the supplementary materials).

To determine the statistical orientations of the fillers, the $< \cos^2\theta >$ values were calculated based on their scattering patterns observed at the (101) peak positions (Figs. 4 and S21) in both PVA/“perfect” filler (graphite) composites (Fig. 4B) and PVA/defective filler (graphite oxide) composites (Fig. 4C). More details related to $< \cos^2\theta >$ calculations are based on previous publication (*61*) and Fig. S21. For randomly oriented structures without preferred orientations, the $< \cos^2\theta >$ value is $\frac{1}{3}$ in both PVA/“perfect” (graphite, 5 vol%) composites (Fig. 4B and Fig. S21E) and PVA/defective filler (graphite oxide, 5 vol%) composites (Fig. 4C and Fig. S21C).

As mentioned earlier, Equations S6-S16 in the supplementary materials highlight the significance of various factors in calculating $k_{11}^{*}, k_{22}^{*}, and \ k_{33}^{*}$ (Fig. 5 and Fig. S22). These factors include the

thermal conductivity of a filler (k_{filler}), the volume fraction of fillers (f), the interfacial thermal resistances (R_{ITR}) of a polymer composite, the thermal conductivity of a polymer matrix ($k_{polymer}$), the aspect ratio of filler ($\frac{a_3}{a_1}$) as illustrated in Fig. S1, and the statistical orientations of the fillers $< cos^2\theta >$ (Fig. S21).

Firstly, we investigate how thermal conductivity of a filler (k_{filler}) and the interfacial thermal resistances (R_{ITR}) of a polymer composite determine the calculated thermal conductivity and measured thermal conductivity in polymer composites (Figs. S22A and S22B). In Fig. S22A, we used the thermal conductivity value for a defective filler (graphite oxide) (k_{filler}), which was based on the measured thermal conductivity (~2000 $W\ m^{-1}\ K^{-1}$) of a "perfect" filler (graphite) in the in-plane direction (*62, 63*). This "perfect" filler (graphite) with measured thermal conductivity ~2000 $W\ m^{-1}\ K^{-1}$ has similar lateral dimensions (a_1 and a_2) and a similar thickness (a_3) to those of our defective fillers (graphite oxide) (*62, 63*). This was done to overestimate the interfacial thermal resistances R_{ITR} in PVA/defective filler (graphite oxide) composites when comparing the measured thermal conductivities in the cross-plane direction in PVA/defective filler (graphite oxide) composites with calculated k_{33}^* using effective medium theory. In Fig. S22B, we used our measured thermal conductivity (~293 $W\ m^{-1}\ K^{-1}$, Fig. 2A) value of pressed "perfect" fillers (graphite) for the thermal conductivity of a "perfect" filler (graphite) (k_{filler}) in the in-plane direction. This was done to underestimate the interfacial thermal resistances R_{ITR} in PVA/"perfect" filler (graphite) composites when comparing the measured thermal conductivities in the cross-plane direction in PVA/defective filler (graphite oxide) composites and calculated k_{33}^* using effective medium theory.

The measured value of $< \cos^2\theta >$ being $\frac{1}{3}$ (Fig. S21C and Fig. S21E in the supplementary materials) is used for the calculated k_{33}^* in Figs. S22A and S22B, in both PVA/"perfect" filler (graphite) composites and PVA/defective filler (graphite oxide) composites. Similarly, the measured thermal conductivities (Fig. 2) of polymer matrices being 0.34 $W\ m^{-1}\ K^{-1}$ are used for the calculated k_{33}^* in the same composites. The measured aspect ratios of 0.02 (Fig. S1C in the supplementary materials) and 0.034 (Fig. S1F in the supplementary materials) are employed to calculate k_{33}^* in PVA/defective filler (graphite oxide) composites and PVA/"perfect" filler (graphite) composites, respectively.

Secondly, we investigate how thermal conductivities of a polymer matrix ($k_{polymer}$) and the interfacial thermal resistances (R_{ITR}) of a polymer composite determine the calculated thermal conductivity and measured thermal conductivity in polymer composites (Figs. S22C and S22D in the supplementary materials). We used the value 0.34 $W\ m^{-1}\ K^{-1}$ for $k_{polymer}$, which was based on the measured thermal conductivity (0.34 $W\ m^{-1}\ K^{-1}$) of polymer (PVA) films (Fig. 2F). It is observed that the measured thermal conductivity of common polymers typically ranges from 0.1 $W\ m^{-1}\ K^{-1}$ to 0.5 $W\ m^{-1}\ K^{-1}$ (*80, 81*). Accordingly, we use values of 0.34 $W\ m^{-1}\ K^{-1}$ and 0.5 $W\ m^{-1}\ K^{-1}$ for $k_{polymer}$ to calculate k_{33}^* values for both PVA/defective filler (graphite oxide) composites and PVA/"perfect" filler (graphite) composites (Fig. S22C and Fig. S22D in the supplementary materials).

The measured value of $< \cos^2\theta >$ value being $\frac{1}{3}$ (Fig. S21C and Fig. S21E in the supplementary

materials) is used for the calculated k_{33}^{*} in Fig. S22C and Fig. S22D, in both PVA/“perfect” filler (graphite) composites and PVA/defective filler (graphite oxide) composites. We choose 2000 $W\ m^{-1}\ K^{-1}$ for k_{filler} in the calculated k_{33}^{*} for PVA/defective filler (graphite oxide) composites, aiming to overestimate the R_{ITR} of polymer composites. We choose 293 $W\ m^{-1}\ K^{-1}$ for k_{filler} in the calculated k_{33}^{*} for PVA/“perfect” filler (graphite) composites, aiming to underestimate the R_{ITR} of polymer composites. The measured aspect ratios of 0.02 (Fig. S1C) and 0.034 (Fig. S1F) are used for calculating k_{33}^{*} in PVA/defective filler (graphite oxide) composites and PVA/“perfect” filler (graphite) composites, respectively (Fig. S22C and Fig. S22B in the supplementary materials).

Thirdly, we investigate how aspect ratios of filler (p) and the interfacial thermal resistances (R_{ITR}) of a polymer composite determine the calculated thermal conductivity and measured thermal conductivity in polymer composites (Fig. S22E and Fig. S22H in the supplementary materials). We used $k_{polymer}$ of 0.34 $W\ m^{-1}\ K^{-1}$ based on the experimental results (Fig. 2F).

In Fig. S22E, we varied p value based on the Gaussian fitting results (Fig. S1C) of 3D profile of defective fillers (graphite oxide), where p is centered at 0.02 and ranges from 0.001 to 0.065, to calculate k_{33}^{*} in PVA/defective filler (graphite oxide) composites. With a lowest p value of 0.001, calculated k_{33}^{*} in PVA/defective filler (graphite oxide) composites was overestimated while the R_{ITR} of the polymer composites was overestimated (*60*). With the highest p value of 0.065, calculated k_{33}^{*} in PVA/defective filler (graphite oxide) composites was underestimated while the R_{ITR} of the polymer composites was underestimated (*60*). In Fig. S22H, we varied p based on the Gaussian fitting results (Fig. S1F) of 3D profile of “perfect” fillers (graphite), where p is centered at 0.034 and ranges from 0.004 to 0.1, to calculate k_{33}^{*} in PVA/“perfect” filler (graphite) composites. Similarly, with a lowest p value of 0.004, calculated k_{33}^{*} in PVA/“perfect” filler (graphite) composites was overestimated while the R_{ITR} of the polymer composites was overestimated. With a highest p value of 0.1, calculated k_{33}^{*} in PVA/“perfect” filler (graphite) composites was underestimated while the R_{ITR} of the polymer composites was underestimated.

The measured value of $< \cos^2\theta >$ being $\frac{1}{3}$ (Fig. S21C and Fig. S21E in the supplementary materials) is used for the calculated k_{33}^{*} in Fig. S20E and Fig. S20F, in both PVA/“perfect” filler (graphite) composites and PVA/defective filler (graphite oxide) composites. We choose 2000 $W\ m^{-1}\ K^{-1}$ for k_{filler} in the calculated k_{33}^{*} for PVA/defective filler (graphite oxide) composites, aiming to overestimate the R_{ITR} of PVA/defective filler (graphite oxide) composites. We choose 293 $W\ m^{-1}\ K^{-1}$ for k_{filler} in the calculated k_{33}^{*} for PVA/“perfect” filler (graphite) composites, aiming to underestimate the R_{ITR} of PVA/“perfect” filler (graphite) composites.

Fourthly, we investigate how thicknesses of filler (a_3) and the interfacial thermal resistances (R_{ITR}) of a polymer composite determine the calculated thermal conductivity and measured thermal conductivity in polymer composites (Fig. S22G and Fig. S22H in the supplementary materials). We used $k_{polymer}$ of 0.34 $W\ m^{-1}\ K^{-1}$ based on the experimental results (Fig. 2F). The value of $< \cos^2\theta >$ value being $\frac{1}{3}$ (Fig. S21C and Fig. S21E in the supplementary materials) is used for the calculated k_{33}^{*} in Fig. S22G and Fig. S22H, in both PVA/“perfect” filler (graphite) composites and PVA/defective filler (graphite oxide) composites. We choose 2000 $W\ m^{-1}\ K^{-1}$ for k_{filler} in

the calculated k_{33}^{*} for PVA/defective filler (graphite oxide) composites, aiming to overestimate the R_{ITR} of PVA/defective filler (graphite oxide) composites. We choose 293 $W\ m^{-1}\ K^{-1}$ for k_{filler} in the calculated k_{33}^{*} for PVA/"perfect" filler (graphite) composites, aiming to underestimate the R_{ITR} of PVA/"perfect" filler (graphite) composites. The measured aspect ratios of 0.02 defective fillers (graphite oxide) (Fig. S1C) and 0.034 in "perfect" fillers (graphite) (Fig. S1F) are used for calculating k_{33}^{*} in PVA/defective composites and PVA/"perfect" filler (graphite) composites, respectively.

In Fig. S22G in the supplementary materials, we varied thicknesses of defective fillers (graphite oxide) (a_3) based on the Gaussian fitting results (Fig. S1B) of 3D profile of defective fillers (graphite oxide), where a_3 is centered at 0.4 μm and ranges from 0.1 μm to 1.5 μm, to calculate k_{33}^{*} in PVA/defective filler (graphite oxide) composites. With a lowest a_3 value of 0.1 μm, calculated k_{33}^{*} in PVA/defective filler (graphite oxide) composites was underestimated while the R_{ITR} of the polymer composites was underestimated (*60*). With the highest a_3 value of 1.5 μm, calculated k_{33}^{*} in PVA/defective filler (graphite oxide) composites was overestimated while the R_{ITR} of the polymer composites was overestimated (*60*). In Fig. S22H in the supplementary materials, we varied thicknesses of "perfect" filler (graphite) (a_3) based on the Gaussian fitting results (Fig. S1E) of 3D profile of "perfect" filler (graphite), where a_3 is centered at 1 μm and ranges from 0.2 μm to 2 μm, to calculate k_{33}^{*} in PVA/"perfect" filler (graphite) composites. Similarly, with a lowest a_3 value of 0.2 μm, calculated k_{33}^{*} in PVA/"perfect" filler (graphite) composites was underestimated while the R_{ITR} of the polymer composites was underestimated. With a highest p value of 2 μm, calculated k_{33}^{*} in PVA/"perfect" filler (graphite) composites was overestimated while the R_{ITR} of the polymer composites was overestimated.

Upon comparing the measured thermal conductivity in the polymer/filler composites in the cross-plane direction with calculated k_{33}^{*}, it is evident that the R_{ITR} values are approximately $\sim 2 \times 10^{-7}$ $m^2\ K\ W^{-1}$ in PVA/defective filler (graphite oxide) composites (Figs. S22A, S22C, S22E, and S22G in the supplementary materials) and $\sim 1 \times 10^{-6}$ $m^2\ K\ W^{-1}$ in PVA/"perfect" filler (graphite) composites (Figs. S22B, S22D, S22F and S22H), respectively.

Section S3. A simple quantum mechanical model for better understanding how dynamic defects enhanced thermal conductivity in polymer composites.

3.1 Simple mechanical model (vibrational Hamiltonian model) setup.

Please note that the phonon frequency/energy and coupling energy in Section S3 are defined in natural unit for equation simplicity. We consider a phonon bath $\omega_{\boldsymbol{q}}$ as the backbone vibrational modes in the polymer, which interacts with the defects. Rather than treating the defect as a static object, here we treat the defect as a two-level system, resembling PW Anderson's treatment on the specific heat on amorphous solids (*66*), with the two levels from the vibrational states of the local defects as ω_1 and ω_2. The total Hamiltonian can be written as

$$H = \sum_{\mathbf{q}} \omega_{\mathbf{q}} \left(a_{\mathbf{q}}^{+} a_{\mathbf{q}} + \frac{1}{2}\right) + \omega_1 \left(a_1^{+} a_1 + \frac{1}{2}\right) + \omega_2 \left(a_2^{+} a_2 + \frac{1}{2}\right)$$
$$+ \sum_{\mathbf{q}j} (V_{\mathbf{q}j} a_{\mathbf{q}}^{+} a_{\mathrm{j}} + V_{\mathbf{q}j}^{*} a_j^{+} a_{\mathbf{q}}) + V_{12} a_1^{+} a_2 + V_{12}^{*} a_2^{+} a_1 \quad \text{(S17)}$$

Where the coefficients V_{qj} denotes the hybridization element between the vibrational defects level *j=1,2* to the polymer **q**, and V_{12} denotes the tunneling between the two defect modes. In Anderson's

work (*66*), from a tunneling picture (*66*), it is shown that $V_{12} = \omega_1 \, exp\left(-\sqrt{\frac{1}{2}mV\Delta x}\right)$.

Here, all operators satisfy the Bosonic canonical commutation relation as

$$[a_m, a_n^+] = \delta_{mn}, m, n = \boldsymbol{q}, 1{,}2 \quad \text{(S18)}$$

3.2 Green's functions.

Since Equation S17 is quadratic, we anticipate the Green's functions have a closed form solution. We define the general retarded Green's functions for both the polymer and the defects as

$$D_{mn}(t-t') = -i\theta(t-t')\langle[a_m(t), a_n^+(t')]\rangle \quad \text{(S19)}$$

Where $m, n = \boldsymbol{q}, 1{,}2$. We adopt the equation of motion method to obtain the Green's functions. Taking time derivative to Equation S19, we have

$$i\partial_t D_{mn}(t-t') = \delta(t-t')\delta_{mn} + i\theta(t-t')\langle[[H, a_m](t), a_n^+(t')]\rangle \quad \text{(S20)}$$

Where the Heisenberg equation-of-motion is used. Now, using the full Hamiltonian Equation S17, we compute the commutator $[H, a_m]$; for any $m \in \{\{\boldsymbol{q}\}, 1{,}2\}$, complement $\bar{m} \equiv \{\{\boldsymbol{q}\}, 1{,}2\} - m$

$$[H, a_m] = -\omega_m a_m - \textstyle\sum_{\bar{m}} V_{m\bar{m}} a_{\bar{m}} \quad \text{(S21)}$$

Substituting Equation S21 back to Equation S20, we have the general equation-of-motion of Green's functions:

$$i\partial_t D_{mn}(t-t') = \delta(t-t')\delta_{mn} + \omega_m D_{mn}(t-t') + \textstyle\sum_{\bar{m}} V_{m\bar{m}} D_{\bar{m}n}(t-t') \quad \text{(S22)}$$

Defining the frequency-domain Green's functions as

$$D_{mn}(\omega) = \int d(t-t') e^{+i(\omega+i0^+)(t-t')} D_{mn}(t-t') \quad \text{(S23)}$$

Then the Green's function equation of motion Equation S22 can be rewritten as

$$(\omega - \omega_m + i0^+) D_{mn}(\omega) - \textstyle\sum_{\bar{m}} V_{m\bar{m}} D_{\bar{m}n}(\omega) = \delta_{mn} \quad \text{(S24)}$$

Or equivalently, writing in explicit form for $m \in \{\{\boldsymbol{q}\}, 1{,}2\}$, we have

$$\begin{aligned}
(\omega - \omega_{\boldsymbol{q}} + i0^+) D_{\boldsymbol{qk}}(\omega) - V_{\boldsymbol{q}1} D_{1\boldsymbol{k}}(\omega) - V_{\boldsymbol{q}2} D_{2\boldsymbol{k}}(\omega) &= \delta_{\boldsymbol{kq}} \\
(\omega - \omega_{\boldsymbol{q}} + i0^+) D_{\boldsymbol{q}1}(\omega) - V_{\boldsymbol{q}1} D_{11}(\omega) - V_{\boldsymbol{q}2} D_{21}(\omega) &= 0 \\
(\omega - \omega_{\boldsymbol{q}} + i0^+) D_{\boldsymbol{q}2}(\omega) - V_{\boldsymbol{q}2} D_{22}(\omega) - V_{\boldsymbol{q}1} D_{12}(\omega) &= 0 \\
(\omega - \omega_1 + i0^+) D_{1\boldsymbol{q}}(\omega) - \textstyle\sum_{\boldsymbol{k}} V_{1\boldsymbol{k}} D_{\boldsymbol{kq}}(\omega) - V_{12} D_{2\boldsymbol{q}}(\omega) &= 0 \\
(\omega - \omega_1 + i0^+) D_{11}(\omega) - \textstyle\sum_{\boldsymbol{k}} V_{1\boldsymbol{k}} D_{\boldsymbol{k}1}(\omega) - V_{12} D_{21}(\omega) &= 1 \quad \text{(S25)} \\
(\omega - \omega_1 + i0^+) D_{12}(\omega) - \textstyle\sum_{\boldsymbol{k}} V_{1\boldsymbol{k}} D_{\boldsymbol{k}2}(\omega) - V_{12} D_{22}(\omega) &= 0 \\
(\omega - \omega_2 + i0^+) D_{2\boldsymbol{q}}(\omega) - \textstyle\sum_{\boldsymbol{k}} V_{2\boldsymbol{k}} D_{\boldsymbol{kq}}(\omega) - V_{21} D_{1\boldsymbol{q}}(\omega) &= 0 \\
(\omega - \omega_2 + i0^+) D_{21}(\omega) - \textstyle\sum_{\boldsymbol{k}} V_{2\boldsymbol{k}} D_{\boldsymbol{k}1}(\omega) - V_{21} D_{11}(\omega) &= 0 \\
(\omega - \omega_2 + i0^+) D_{22}(\omega) - \textstyle\sum_{\boldsymbol{k}} V_{2\boldsymbol{k}} D_{\boldsymbol{k}2}(\omega) - V_{21} D_{12}(\omega) &= 1
\end{aligned}$$

This is a set of 9 linear equations with 9 unknown variables, and thus can be solved out explicitly. For later computational convenience, we also need a set of adjoint equations. This can be obtained by either taking the derivative w.r.t. *t'* in Equation S19, or using the Lehmann representation of Green's function, which is more general. The frequency-domain Green's functions can be written as

$$D_{mn}(\omega) = \frac{1}{Z}\textstyle\sum_{pq}\left[\frac{e^{-\beta E_p}\langle p|a_m|q\rangle\langle q|a_n^+|p\rangle}{\omega+E_p-E_q+i0^+} - \frac{e^{-\beta E_p}\langle p|a_n^+|q\rangle\langle q|a_m|p\rangle}{\omega+E_q-E_p+i0^+}\right] \quad \text{(S26)}$$

from which we immediately obtain $D_{mn}(\omega) = D_{nm}^*(\omega)$. Substituting into Equation S24, we obtain

$$(\omega - \omega_n - i0^+) D_{mn}(\omega) - \textstyle\sum_{\bar{n}} V_{n\bar{n}}^* D_{m\bar{n}}(\omega) = \delta_{mn} \quad \text{(S27)}$$

Explicitly, we have two of the following equations that come handy later:

$$D_{\alpha\boldsymbol{k}}(\omega) = \frac{V_{\boldsymbol{k}1}^* D_{\alpha 1}(\omega) + V_{\boldsymbol{k}2}^* D_{\alpha 2}(\omega)}{\omega - \omega_{\boldsymbol{k}} - i0^+} = \sum_{\beta=1,2} \frac{V_{\boldsymbol{k}\beta}^* D_{\alpha\beta}(\omega)}{\omega - \omega_{\boldsymbol{k}} - i0^+}, \alpha = 1,2 \quad \text{(S28)}$$

3.3 Phonon Green's function in polymer matrix.

We now are ready to compute the phonon Green's function in the polymer $D_{\boldsymbol{qk}}(\omega)$. Label above 9 equations in Equation (S25) as (a)-(i). Equations (b) and (c) can be rewritten as

$$D_{\boldsymbol{k}1}(\omega) = \frac{V_{\boldsymbol{k}1} D_{11}(\omega) + V_{\boldsymbol{k}2} D_{21}(\omega)}{\omega - \omega_{\boldsymbol{k}} + i0^+}$$
$$D_{\boldsymbol{k}2}(\omega) = \frac{V_{\boldsymbol{k}2} D_{22}(\omega) + V_{\boldsymbol{k}1} D_{12}(\omega)}{\omega - \omega_{\boldsymbol{k}} + i0^+} \quad \text{(S29)}$$

Substituting the Equation S29 back to Equations (e) and (h) to cancel out $D_{\boldsymbol{k}1}(\omega)$, and similarly back to (f) and (i) to cancel out $D_{\boldsymbol{k}2}(\omega)$, and define coefficients

$$\lambda_{11}(\omega) = \sum_{\boldsymbol{k}} \frac{V_{1\boldsymbol{k}} V_{\boldsymbol{k}1}}{\omega - \omega_{\boldsymbol{k}} + i0^+}, \lambda_{12}(\omega) = \sum_{\boldsymbol{k}} \frac{V_{1\boldsymbol{k}} V_{\boldsymbol{k}2}}{\omega - \omega_{\boldsymbol{k}} + i0^+},$$
$$\lambda_{21}(\omega) = \sum_{\boldsymbol{k}} \frac{V_{2\boldsymbol{k}} V_{\boldsymbol{k}1}}{\omega - \omega_{\boldsymbol{k}} + i0^+}, \lambda_{22}(\omega) = \sum_{\boldsymbol{k}} \frac{V_{2\boldsymbol{k}} V_{\boldsymbol{k}2}}{\omega - \omega_{\boldsymbol{k}} + i0^+} \quad \text{(S30)}$$

Then we have the following equations only containing the dynamical defects' Green's functions:

$$(\omega - \omega_1 - \lambda_{11}(\omega))D_{11}(\omega) - (\lambda_{12}(\omega) + V_{12})D_{21}(\omega) = 1$$
$$(\omega - \omega_2 - \lambda_{22}(\omega))D_{21}(\omega) - (\lambda_{21}(\omega) + V_{21})D_{11}(\omega) = 0$$
$$(\omega - \omega_1 - \lambda_{11}(\omega))D_{12}(\omega) - (\lambda_{12}(\omega) + V_{12})D_{22}(\omega) = 0$$
$$(\omega - \omega_2 - \lambda_{22}(\omega))D_{22}(\omega) - (\lambda_{21}(\omega) + V_{21})D_{12}(\omega) = 1 \quad \text{(S31)}$$

Solving linear equation of Equation S31, we have defect Green's functions

$$D_{11}(\omega) = -\frac{\omega - \omega_2 - \lambda_{22}(\omega)}{g(\omega)}, D_{12}(\omega) = -\frac{\lambda_{12}(\omega) + V_{12}}{g(\omega)}$$
$$D_{21}(\omega) = -\frac{\lambda_{21}(\omega) + V_{21}}{g(\omega)}, D_{22}(\omega) = -\frac{\omega - \omega_1 - \lambda_{11}(\omega)}{g(\omega)} \quad \text{(S32)}$$

Where $g(\omega) \equiv (\lambda_{12}(\omega) + V_{12})(\lambda_{21}(\omega) + V_{21}) - (\omega - \omega_1 - \lambda_{11}(\omega))(\omega - \omega_2 - \lambda_{22}(\omega))$.
Now substituting Equation S28 back to Equation (a) in Equation S25, we have

$$D_{\boldsymbol{qk}}(\omega) = \frac{\delta_{\boldsymbol{qk}}}{\omega - \omega_{\boldsymbol{q}} + i0^+} + \frac{\sum_{\alpha,\beta=1}^{2} V_{\boldsymbol{q}\alpha} V_{\boldsymbol{k}\beta}^* D_{\alpha\beta}(\omega)}{(\omega - \omega_{\boldsymbol{q}} + i0^+)(\omega - \omega_{\boldsymbol{k}} - i0^+)} \quad \text{(S33)}$$

Or write down explicitly using Equation S32, we have the final form

$$D_{\boldsymbol{qk}}(\omega) = \frac{\delta_{\boldsymbol{qk}}}{\omega - \omega_{\boldsymbol{q}}} - \frac{1}{(\omega - \omega_{\boldsymbol{q}})(\omega - \omega_{\boldsymbol{k}})g(\omega)} \begin{pmatrix} V_{\boldsymbol{q}1} V_{\boldsymbol{k}1}^* (\omega - \omega_2 - \lambda_{22}(\omega)) + V_{\boldsymbol{q}1} V_{\boldsymbol{k}2}^* (\lambda_{12}(\omega) + V_{12}) \\ + V_{\boldsymbol{q}2} V_{\boldsymbol{k}1}^* (\lambda_{21}(\omega) + V_{21}) + V_{\boldsymbol{q}2} V_{\boldsymbol{k}2}^* (\omega - \omega_1 - \lambda_{11}(\omega)) \end{pmatrix} \quad \text{(S34)}$$

which is the Green's function of the polymer phonon dispersion $\omega_{\boldsymbol{q}}$, after interacting with the

dynamical defects with two energy levels.

Consider the case where the two levels of defects hybridize with the polymer backbone are the same, so $V_{\boldsymbol{k}1}^* = V_{\boldsymbol{k}2}^* = V_{\boldsymbol{k}}^*, V_{\boldsymbol{k}1} = V_{\boldsymbol{k}2} = V_{\boldsymbol{k}}$, $\lambda_{11}(\omega) = \lambda_{12}(\omega) = \lambda_{21}(\omega) = \lambda_{22}(\omega) = \lambda(\omega) = \sum_{\boldsymbol{k}} \frac{|V_{\boldsymbol{k}}|^2}{\omega - \omega_{\boldsymbol{k}}}$, and $V_{12} = V_{21}$,$g(\omega) \equiv (\lambda(\omega) + V_{12})(\lambda(\omega) + V_{21}) - (\omega - \omega_1 - \lambda(\omega))(\omega - \omega_2 - \lambda(\omega))$ then Equation S34 can be simplified, where the $-\lambda_{22}(\omega)$ and $\lambda_{12}(\omega)$ terms are canceled out since $V_{\boldsymbol{k}1}^* = V_{\boldsymbol{k}2}^*$. The simplified Greens function is given by

$$D_{\boldsymbol{qk}}(\omega) = \frac{\delta_{\boldsymbol{qk}}}{\omega - \omega_{\boldsymbol{q}}} - \frac{2V_{\boldsymbol{q}}V_{\boldsymbol{k}}^*\left(\omega - \frac{\omega_1 + \omega_2}{2} + V_{12}\right)}{(\omega - \omega_{\boldsymbol{q}})(\omega - \omega_{\boldsymbol{k}})g(\omega)} \quad \text{(S35)}$$

Where the coefficients are defined as $g(\omega) \equiv (\lambda(\omega) + V_{12})^2 - (\omega - \omega_1 - \lambda(\omega))(\omega - \omega_2 - \lambda(\omega))$, and $\lambda(\omega) = \sum_{\boldsymbol{k}} \frac{|V_{\boldsymbol{k}}|^2}{\omega - \omega_{\boldsymbol{k}} + i0^+}$. In the case where the two defects are equal with $\omega_1 = \omega_2 = \omega_0$, i.e. two-level degeneracy, the Equation S35can further be simplified as

$$D_{\boldsymbol{qk}}(\omega) = \frac{\delta_{\boldsymbol{qk}}}{\omega - \omega_{\boldsymbol{q}}} + \frac{2V_{\boldsymbol{q}}V_{\boldsymbol{k}}^*}{(\omega - \omega_{\boldsymbol{q}})(\omega - \omega_{\boldsymbol{k}})(\omega - \omega_0 - 2\lambda(\omega) - V_{12})} \quad \text{(S36)}$$

To proceed, imagine there are many defects there, performing impurity average procedure, on average the phonon momentum will not change (*68*), i.e., $\langle V_{\boldsymbol{q}}V_{\boldsymbol{k}}^* \rangle = n_i |V_{\boldsymbol{k}}|^2 \delta_{\boldsymbol{qk}}$, in which n_i represents the density of the impurities, or equivalently filler volume fraction. For weak scattering regime, the impurity-averaged phonon propagator from Equation S35 can be written as

$$D_{\boldsymbol{k}}(\omega) = \langle D_{\boldsymbol{qk}}(\omega) \rangle \approx \frac{1}{\omega - \omega_{\boldsymbol{k}} - \Sigma(\boldsymbol{k}, \omega)} \quad \text{(S37)}$$

Where the phonon self-energy correction $\Sigma(\boldsymbol{k}, \omega)$ can be written as

$$\Sigma(\boldsymbol{k}, \omega) = -\frac{2n_i|V_{\boldsymbol{k}}|^2\left(\omega - \frac{\omega_1 + \omega_2}{2} + V_{12}\right)}{g(\omega)} \quad \text{(S38)}$$

For the degenerate defects $\omega_1 = \omega_2 = \omega_0$, which can be used to describe the perfect fillers (and defective fillers in an approximate way), we have

$$\Sigma(\boldsymbol{k}, \omega) = \frac{n_i|V_{\boldsymbol{k}}|^2}{\frac{\omega - \omega_0 - V_{12}}{2} - \lambda(\omega)} \quad \text{(S39)}$$

from which we can readily write down the real and imaginary parts as

$$Re\,\Sigma\,(\boldsymbol{k}, \omega) = \frac{n_i|V_{\boldsymbol{k}}|^2\left(\frac{\omega - \omega_0 - V_{12}}{2} - Re\,\lambda(\omega)\right)}{\left(\frac{\omega - \omega_0 - V_{12}}{2} - Re\,\lambda(\omega)\right)^2 + (Im\,\lambda(\omega))^2}$$
$$Im\,\Sigma\,(\boldsymbol{k}, \omega) = \frac{n_i|V_{\boldsymbol{k}}|^2\,Im\,\lambda(\omega)}{\left(\frac{\omega - \omega_0 - V_{12}}{2} - Re\,\lambda(\omega)\right)^2 + (Im\,\lambda(\omega))^2} \quad \text{(S40)}$$

Where we have

$$Re\,\lambda\,(\omega) = \sum_{\boldsymbol{k}} P \frac{|V_{\boldsymbol{k}}|^2}{\omega - \omega_{\boldsymbol{k}}} \quad \text{(S41)}$$

$$Im\,\lambda\,(\omega) = -\pi \sum_{\boldsymbol{q}} \left|V_{\boldsymbol{q}}\right|^2 \delta(\omega - \omega_{\boldsymbol{q}}) \quad \text{(S42)}$$

<u>3.4 Kubo formula for lattice thermal conductivity of defective polymers.</u>

The generic thermal conductivity computed from the normal Green's function approach can be written as (*82*)

$$\kappa(T) = \frac{k_B\beta}{3L^3} \lim_{\delta \to 0} \int_0^{+\infty} e^{-\delta t} dt \int_0^{\beta} d\lambda \langle \boldsymbol{S}(0) \cdot \boldsymbol{S}(t + i\lambda) \rangle \quad \text{(S43)}$$

Where the energy flow vector operator S can be written as $\boldsymbol{S}(t) = \sum_{\boldsymbol{k}} v_{\boldsymbol{k}}\omega_{\boldsymbol{k}}n_{\boldsymbol{k}}(t)$, with$n_{\boldsymbol{k}} = b_{\boldsymbol{k}}^{+}b_{\boldsymbol{k}}$ is the phonon number density operator, $v_{\boldsymbol{k}}$ is the phonon group velocity and $\omega_{\boldsymbol{k}}$is the dispersion. It has been shown that the phonon thermal conductivity Equation S41 can be rewritten in terms of the phonon Green's function as (*67*)

$$\kappa(T) = \frac{k_B\beta^2}{3\pi L^3}\sum_{\boldsymbol{kq}} \boldsymbol{v_k}\cdot\boldsymbol{v_q}\omega_{\boldsymbol{k}}\omega_{\boldsymbol{q}} \times \int_{-\infty}^{+\infty} d\omega \frac{e^{+\beta\omega}}{(e^{\beta\omega}-1)^2} Im\, D_{\boldsymbol{qk}}\,(\omega)\, Im\, D_{\boldsymbol{kq}}\,(\omega) \quad \text{(S44)}$$

Where the phonon Green's function is defined as retarded form of Equation S19to ensure consistency, and no other more common form of displacement-displacement correlator. In the case of phonon interacting with a two-level defects, $D_{\boldsymbol{qk}}(\omega)$ can be expressed in terms of Equation S35.

As a sanity check, if we have phonon propagation written as

$$D_{\boldsymbol{qk}}(\omega) = \frac{\delta_{\boldsymbol{qk}}}{\omega-\omega_{\boldsymbol{k}}+i\Gamma_{\boldsymbol{k}}(\omega)} \quad \text{(S45)}$$

Then we have

$Im\, D_{\boldsymbol{qk}}\,(\omega)\, Im\, D_{\boldsymbol{kq}}\,(\omega) = \delta_{\boldsymbol{kq}}\left(\frac{\Gamma_{\boldsymbol{k}}(\omega)}{(\omega-\omega_{\boldsymbol{k}})^2+\Gamma_{\boldsymbol{k}}^2(\omega)}\right)^2 \approx \frac{\pi\delta_{\boldsymbol{kq}}\Gamma_{\boldsymbol{k}}(\omega)\delta(\omega-\omega_{\boldsymbol{k}})}{(\omega-\omega_{\boldsymbol{k}})^2+\Gamma_{\boldsymbol{k}}^2(\omega)}$, and the total thermal conductivity Equation S44 can be written as

$$\kappa(T) = \frac{k_B\beta^2}{3L^3}\sum_{\boldsymbol{k}} v_{\boldsymbol{k}}^2\omega_{\boldsymbol{k}}^2 \frac{e^{+\beta\omega}}{(e^{\beta\omega}-1)^2}\frac{1}{\Gamma_{\boldsymbol{k}}(\omega)} = \frac{1}{3}\sum_{\boldsymbol{k}} v_{\boldsymbol{k}}^2\tau_{\boldsymbol{k}}\, C_{\boldsymbol{k}} \quad \text{(S46)}$$

Where $\tau_{\boldsymbol{k}} = \frac{1}{\Gamma_{\boldsymbol{k}}}$ and$\boldsymbol{C_k} = \frac{\boldsymbol{\omega_k\partial_T n_B(\omega_k)}}{\boldsymbol{L^3}}$ are phonon relaxation time and specific heat of a phonon with wavevector **k,** $\boldsymbol{n_B(\omega_k)}$ is the Bosonic occupation.

As to thermal diffusivity, we need the total specific heat capacity,

$$\begin{aligned} C(T) &= \sum_{\boldsymbol{k}} C_{\boldsymbol{k}}(T) = \sum_{\boldsymbol{k}} \frac{\omega_{\boldsymbol{k}}\partial_T n_B(\omega_{\boldsymbol{k}})}{L^3} = \frac{1}{L^3}\sum_{\boldsymbol{k}} \frac{\omega_{\boldsymbol{k}}^2 e^{\beta\omega_{\boldsymbol{k}}}}{T^2(e^{\beta\omega_{\boldsymbol{k}}}-1)^2} \\ &= \int d\omega \mathrm{Dos}(\omega)\frac{\omega^2 e^{\beta\omega}}{T^2(e^{\beta\omega}-1)^2} \end{aligned} \quad \text{(S47)}$$

In all formula, we can consider the phonon energy $\omega_{\boldsymbol{k}}$we should use the renormalized phonon energy $\omega_{\boldsymbol{k}}'$,

$$\begin{aligned} \omega_{\boldsymbol{k}}' &= \omega_{\boldsymbol{k}} + Re\,\Sigma\,(\boldsymbol{k},\omega) \\ \Gamma_{\boldsymbol{k}}(\omega) &= -\,Im\,\Sigma\,(\boldsymbol{k},\omega) \end{aligned} \quad \text{(S48)}$$

Now, the overarching goal is clear, that we would like to explain the decrease of specific heat capacity $C(T)$ with respect to filler fraction n_i using Equation S47, while the contradictory observation that the $\kappa(T)$ thermal conductivity actually increases using Equation S44. Normally, due to Equation S46, specific heat capacity and thermal conductivity trend are the same.

<u>3.5 The connection of the experimental data.</u>

The main goal to explain the data as the following, where $C(T)$ decreases vs n_i, while $\kappa(T)$ increases vs n_i, even though from a kinetic model Equation S46 they are tightly linked to each other and often share the same trend. To see that, we notice that in the formula of $C(T)$ Equation S47, each term $C_{\boldsymbol{k}} = \omega_{\boldsymbol{k}}\partial_T n_B(\omega_{\boldsymbol{k}})$ is a monotonically decrease function of phonon energy $\omega_{\boldsymbol{k}}$, and therefore the decrease of $C_{\boldsymbol{k}}$ shall be linked to an increase of $\omega_{\boldsymbol{k}}$. According to Equation S48, that means $Re\,\Sigma\,(\boldsymbol{k},\omega) > 0$.

On the other hand, after defect scattering, $\tau_{\boldsymbol{k}}$ decreases, and according to Equation S46, the only possibility is an enhancement of phonon velocity $v_{\boldsymbol{k}}$.

$$v_{\boldsymbol{k}} = \partial_{\boldsymbol{k}}\omega_{\boldsymbol{k}} + \partial_{\boldsymbol{k}}\, Re\, \Sigma\, (\boldsymbol{k}, \omega) \quad \text{(S49)}$$

i.e. a very large $\boldsymbol{k}$-variation of the reciprocal-space of the phonon self energy. Overall, by comparing with Equation S40, we anticipate that means $\frac{dV_{\boldsymbol{k}}}{d\boldsymbol{k}}$ large, which could mean a large heterogeneity that increases phonon group velocity and dominates over the scattering mechanism and the resulted reduction of lifetime and heat capacity. More quantitative calculations can be done but the contradicting behaviors of $C(T)$ and $\kappa(T)$ constrain the possibilities:

$$\begin{gathered} \omega_{\boldsymbol{k}} \uparrow, v_{\boldsymbol{k}} \uparrow\uparrow, \\ \tau_{\mathbf{k}} \downarrow, C_{\mathbf{k}} \downarrow\downarrow, \\ C(T) \downarrow, \kappa(T) \uparrow \end{gathered} \qquad \text{(S50)}$$

3.6 Numerical analysis.

To quantify the trend of $C(T)$, and $\kappa(T)$ as a function of filler fraction n_i, we assume $\omega_{\boldsymbol{k}}$ to take the form of the phonon dispersion of 1-D polymer chain. Furthermore, we consider $\omega_{\mathbf{1}}$ is a constant, assuming a single vibrational state defect. The peak value of ω_k and ω_1 is related to the square root of force constant between the polymer backbone and the fillers obtained by fitting quadratic function near the bond length between the beads (*65*). V_k is assumed to take the form of Lorentzian function peaked at the center between Gamma point and the zone boundary, where the peak value of V_k scale with the force constant between the polymer and filler (*65*). The numerical analysis is performed using the parameters in Table S1.

Finally, it is also observed that the heat capacity $C(T)$ with defective fillers is consistently lower than that of the perfect fillers, while the thermal conductivity $\kappa(T)$ of polymer composites with defective fillers is consistently higher. This can be readily explained through the additional vibrational defects level, by assuming $\omega_1 \neq \omega_2$ in Equation S39. Under the condition that the effective phonon group velocity increases, shown schematically in Equation S50, then the observation can be fully reproduced. Enhanced vibrational coupling between the polymer and the defective filler at the polymer/filler interface in PVA/defective filler (graphite oxide) composites could lower interfacial thermal resistance and improve thermal conductivity relative to PVA/"perfect" filler (graphite) composites. The ratio $\frac{\omega_1}{\omega_k}$ and $\frac{V_k}{\omega_k}$ from the force constant in Figs. 6E-F are approximately 1.25 and 0.1, respectively which shows the decreasing trend of $C(T)$ and increasing trend of $\kappa(T)$ as observed in the experiment.

Section S4. Molecular dynamics (MD) simulations to understand thermal transport mechanisms in polymer composites.

MD simulations were performed using the Large-scale Atomic/Molecular Massively Parallel Simulator (LAMMPS) software package (*83, 84*). The initial structures for PVA/"perfect" filler (graphite) composites and PVA/defective filler (graphite oxide) composites were constructed starting from a 10 unit cells × 10 unit cells × 10 unit cells graphite model. The top and bottom graphite layers in PVA/defective filler (graphite oxide) composites were connected to 10 hydroxyl (-OH) groups each with a C/O ratio of 20:1. The 36 PVA chains with 10 repeat units in each chain were packed on the top of "perfect" filler (graphite) and defective filler (graphite-oxide). Polymer Consistent Force Field was used in this work (*85-89*). Periodic boundary conditions were used in all three dimensions. The units and atom style were set in the simulations as real and full respectively, whereas 0.5 fs was used as the timestep. 10 Å cutoff distance was used for Lennard-Jones and coulombic interactions. Long-range coulombic interactions beyond cutoff distance were calculated using Ewald summation with an accuracy of 0.0001. Non-bonded (pairwise) interactions between permanently bonded (either directly or via one intermediate bond) pair of atoms were excluded using special bonds command considering both Lennard-Jones and coulombic interactions with weighing factors 0 0 1 (*86, 87*). The neighbor lists were built using bin style after every timestep if necessary, with a skin distance of 2 Å. The initial velocity of the system was created using gaussian distribution corresponding to a temperature of 300 K while zeroing the linear and angular momentum. Three independent simulation runs were done for calculating the interfacial thermal conductance and roughness. The simulation steps are as follows:

Step 1: The systems were relaxed in NPT (constant mass, pressure and temperature) ensemble using Nose-Hoover Barostat and Thermostat for 0.75 ns. The pressure was set as 1 atmosphere with each dimension controlled independently using the tri keyword and the damping parameter of 500 fs was used for the Barostat. The temperature was set as 300 K and the damping parameter of 50 fs was used for the thermostat.

Step 2: The systems were further relaxed in NVT (constant mass, volume and temperature) ensemble using Nose-Hoover Thermostat for 0.75 ns. The temperature was set as 300 K with a damping parameter of 50 fs.

Step 3: The systems were stabilized in NVE (constant mass, volume and energy) ensemble for 1 ns (interfacial thermal conductance and roughness calculations) and 0.75 ns (RDF, MSD and DOS calculations). In interfacial thermal conductance and roughness simulations, the heat values were applied in the hot and cold regions so that there is a constant heat flow in the system. After the system reaches steady state, the temperature profile across the interfaces and the whole system could be obtained.

Step 4 (Production run): The coordinates and temperature of all the atoms at every timestep were averaged for a certain time (e.g., 1.5 ns) in the NVE ensemble for interfacial thermal conductance and roughness simulations while applying the heat values. The Radial Distribution Function (RDF) and coordination number for carbons in PVA with respect to graphite (or graphite oxide) carbons were computed up to the cutoff distance (divided in 1000 bins) at every timestep and averaged for

1 ns in the NVE ensemble. Mean-squared displacement (MSD) for PVA carbons near the interface was outputted after every 1 ps for 1 ns in the NVE ensemble. Normalized velocity autocorrelation for graphite carbons, graphite oxide carbons and PVA carbons near the interface was outputted at every timestep for 1 ns in the NVE ensemble for the density of states (DOS) calculations.

The roughness of the top graphite layer in PVA/"perfect" filler (graphite) composites and PVA/defective filler (graphite oxide) composites is shown in Fig. S24A. The root mean square (RMS) roughness (R) is defined as:

$$R = \sqrt{\frac{1}{n}\sum_{j=1}^{n}\left(z_j - \mu\right)^2} \quad \text{(S51)}$$

Where n represents the total number of carbon atoms in the top graphite layer, z represents the z-coordinate of each carbon atom in the top graphite layer, and μ represents the average z-coordinate of all the carbon atoms in the top graphite layer. The roughness was calculated using the z-coordinates averaged over the production run. The roughness of the top graphite layer in PVA/defective filler (graphite oxide) and PVA/"perfect" filler (graphite) composites, shown in Fig. S24, indicates that the roughness in PVA/defective filler (graphite oxide) composites is an order of magnitude higher than in PVA/"perfect" filler (graphite) composites. Moreover, the roughness values are consistent at different heat values indicating no apparent dependence (Fig. S24A).

We determined the radial distribution function (RDF, Fig S24B) and the coordination number for PVA carbons with respect to graphite carbons (or graphite oxide carbons) in the PVA/"perfect" filler (graphite) composites and PVA/defective filler (graphite oxide) composites. As shown in Fig. S24B, the RDF magnitude for PVA carbons in the PVA/"perfect" filler (graphite) composites is higher than that in the PVA/defective filler (graphite oxide) composites. This suggests that more PVA carbon atoms are closer to the graphite surface in the PVA/"perfect" filler (graphite) composites compared to the PVA/defective filler (graphite oxide) composites. The coordination number plot in Figs. S24C-S24D suggest that fewer PVA chains are near the graphite oxide surface in the PVA/defective filler (graphite oxide) composites compared to the PVA/"perfect" filler (graphite) composites. These differences observed in (Fig. S24B and Fig. S24D) may be attributed to the increased roughness of the graphite oxide layer caused by the functional groups such as hydroxyl (-OH) group. The results in Fig. S24 support our neutron scattering findings (Figs. 8 and S26), showing fewer PVA chains near the graphite surface in PVA/defective filler (graphite oxide) composites compared to PVA/"perfect" filler (graphite) composites, which will be discussed in the Section S5.

To further understand the reason for greater interfacial thermal conductance for PVA/defective filler (graphite oxide) composites despite having fewer PVA chains closer to the surface, we calculated the density of states of carbon atoms near the PVA/filler interface in PVA composites (Fig. S23) and the mean-squared displacement (MSD) of the PVA carbons near the PVA/filler interface (Fig. S24E). The density of states analysis shown in Fig. S23 indicates that while sharper vibrational modes are present near the PVA-filler interface in the PVA/"perfect" filler (graphite) composites, the broader modes observed in the PVA/defective filler (graphite oxide) composites enable stronger coupling between the filler and PVA carbons. Additionally, the PVA carbons in the defective filler composites show more modes at lower frequencies, further enhancing this vibrational interaction. These factors contribute to stronger interfacial vibrational coupling in the PVA/defective filler (graphite oxide) composites, resulting in higher interfacial thermal

conductance and lower interfacial thermal resistance compared to the PVA/“perfect” filler (graphite) composites (Fig. 7). Fig. S24E clearly shows that MSD for PVA carbons near the interface is higher in PVA/defective filler (graphite oxide) composites as compared to PVA/“perfect” filler (graphite) composites. This suggests that PVA/defective filler (graphite oxide) composites allow more freedom of movement for the PVA chains near PVA-filler interface, facilitating heat transfer and resulting in greater interfacial thermal conductance and lower interfacial thermal resistance in the PVA/defective filler (graphite oxide) composites.

Section S5. Quasielastic neutron scattering (QENS) experiments and elastic incoherent scattering factor (EISF) analyses.

The dynamics of polyvinyl alcohol (PVA) film, PVA/“perfect” filler (graphite, 5 vol%) composites, and PVA/defective filler (graphite oxide, 5 vol%) composites were analyzed using the quasielastic neutron scattering (QENS) technique. The measured QENS data were reduced and analyzed using MantidPlot (*90*) and QClimax (*91*) software, respectively.

$$I(Q,E) = \left[X(Q)\delta(E) + \left(1 - X(Q)\right)S(Q,E)\right] \otimes R(Q,E) + B(Q,E) \quad \text{(S52)}$$

We analyzed the measured QENS spectra, $I(Q,E)$, using Equation S52, where the relative spectral weight, $X(Q)$, which is characterized by a delta function. $\delta(E)$ accounts for the contribution from elastic scattering. The dynamics of the polymers, rich in hydrogen, are well represented by $S(Q,E)$, called the dynamic structure factor. $S(Q,E)$ was modeled to a Cole-Cole function (*92*), as described in Equation S53, to extract the dynamics information. During the analysis, the dynamic structure factor is convoluted with the instrument resolution function, $R(Q,E)$, and a linear background term, $B(Q,E)$, which is added to account for the faster dynamics during the data analysis.

$$S(Q,E) = \frac{1}{\pi E_0(Q)}\left[\frac{\left(\frac{E}{E_0(Q)}\right)^{-\alpha(Q)}\cos\frac{\pi\alpha(Q)}{2}}{1+2\left(\frac{E}{E_0(Q)}\right)^{1-\alpha(Q)}\sin\frac{\pi\alpha(Q)}{2}+\left(\frac{E}{E_0(Q)}\right)^{2(-\alpha(Q))}}\right] \quad \text{(S53)}$$

Where $E_0(Q)$ is the half-width at half maximum (HWHM) of the quasielastic peak. α is the stretched exponent. When $\alpha = 0,$ Equation S53 becomes a Lorentzian function. The Q-dependence of half-width at half-maximum provides the nature of the dynamics processes present in the samples.

The elastic incoherent scattering factor (EISF) (Fig. S26) obtained from PVA, PVA/“perfect” filler (graphite, 5 vol%) composites, and PVA/defective filler (graphite oxide, 5 vol%) composites showed that the elastic intensity from PVA/“perfect” filler (graphite, 5 vol%) sample was systematically higher compared to that from PVA/defective filler (graphite oxide, 5 vol%) sample at all tested temperatures. This result suggests that fewer PVA chains are observed near the graphite surface in the PVA/defective filler (graphite oxide) composites compared to those in the PVA/“perfect” filler (graphite) composites which is likely due to the rougher surface for the PVA-filler interface in the PVA/defective filler (graphite oxide, 5 vol%) sample as discussed in the MD simulation section (Section S4). The anticipated arrangement of PVA molecules around the filler particles is presented in Fig. S27.

Section S6. Supporting figures and data.

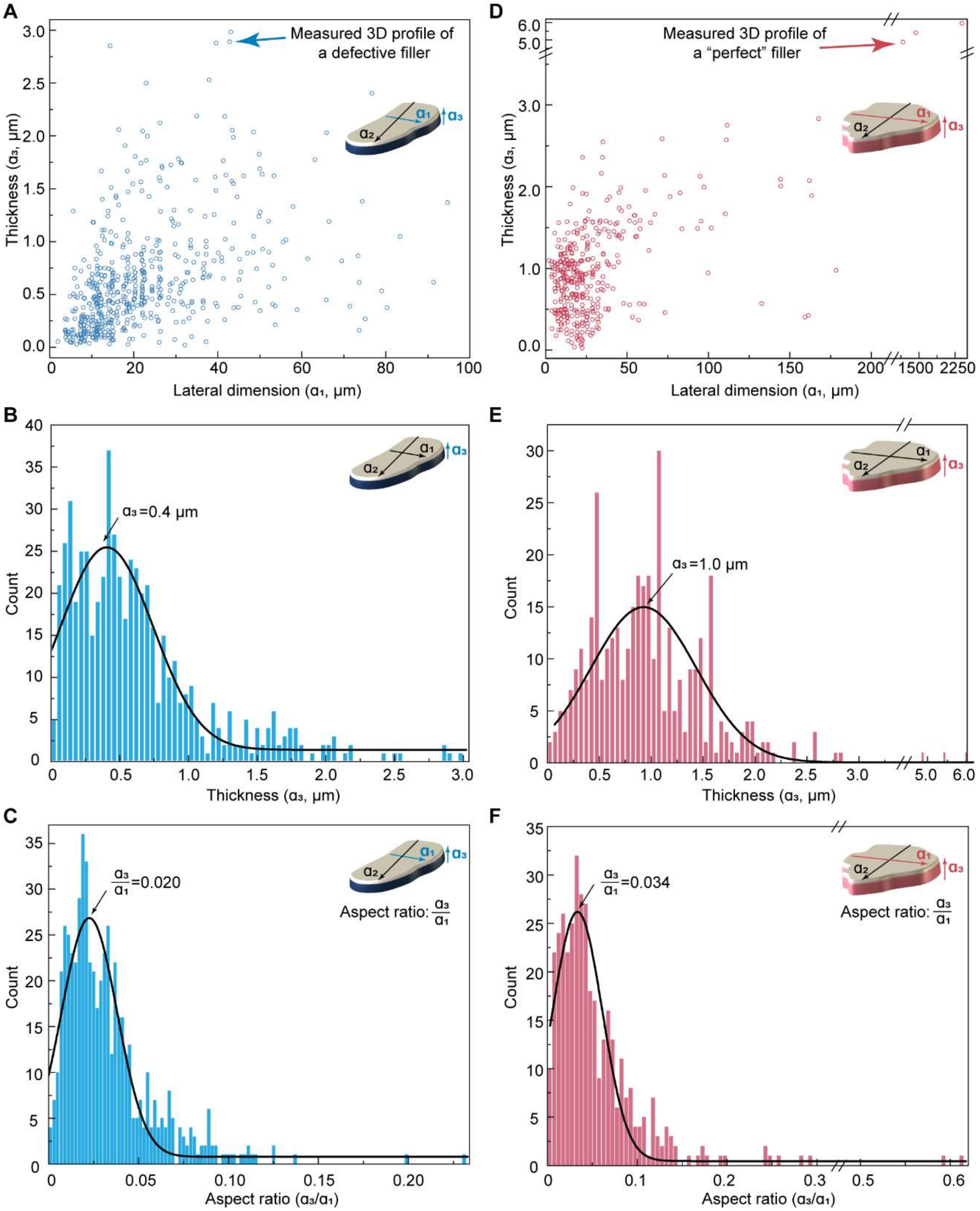

Fig. S1. Statistical analysis of lateral sizes (a_1), thicknesses (a_3), and aspect ratios ($\frac{a_3}{a_1}$) in "perfect" fillers (graphite) and defective fillers (graphite oxide) measured by a Zygo's three-dimensional (3D) optical profiler. To achieve statistical $\frac{a_3}{a_1}$ of fillers, 3D profiles of 384 pieces of "perfect" fillers (graphite) and 570 pieces of defective fillers (graphite oxide) were measured. (A) A singular circle symbol in this figure represented measured a_1 and a_3 of a singular

defective filler (graphite oxide). There were 570 circle symbols in total that represent measured 3D profiles of 570 different pieces of defective fillers (graphite oxide). **(B)** Relationships between counts and thicknesses (a_3) of defective fillers (graphite oxide). The data is plotted with a bin size of 0.04 and fitted with Gauss function using Origin Pro software. Such selection of the bin size is to enable the high resolution of filler thickness below 0.5 μm. Based on the Gauss fitting results, the thicknesses for the graphite oxide fillers range from 0.1 μm to 1.0 μm, with a center at 0.4 μm. **(C)** Relationships between counts and aspect ratios ($\frac{a_3}{a_1}$) of defective fillers (graphite oxide). The data is plotted with a bin size of 0.002 and fitted with Gauss function using Origin Pro software. Such selection of the bin size is to enable the high resolution of filler aspect ratio below 0.025. Based on the Gauss fitting results, the aspect ratios for the graphite oxide fillers range from 0.001 to 0.065, with a center at 0.020. **(D)** A singular circle symbol in this figure represented measured a_1 and a_3 of a singular "perfect" filler (graphite). There were 384 circles in total that represent measured 3D profiles of 384 different pieces of "perfect" fillers (graphite). **(E)** Relationships between counts and thicknesses (a_3) of "perfect" fillers (graphite). The data is plotted with a bin size of 0.05 and fitted with Gauss function using Origin Pro software. Such selection of the bin size is to enable the high resolution of filler thickness below 1.0 μm. Based on the Gauss fitting results, the thicknesses for the graphite oxide fillers range from 0.2 μm to 2.0 μm, with a center at 1.0 μm. **(F)** Relationships between counts and aspect ratios ($\frac{a_3}{a_1}$) of "perfect" fillers (graphite). The data is plotted with a bin size of 0.005 and fitted with Gauss function using Origin Pro software. Such selection of the bin size is to enable the high resolution of filler aspect ratio below 0.05. Based on the Gauss fitting results, the aspect ratios for the graphite filler range from 0.004 to 0.1, with a center at 0.034.

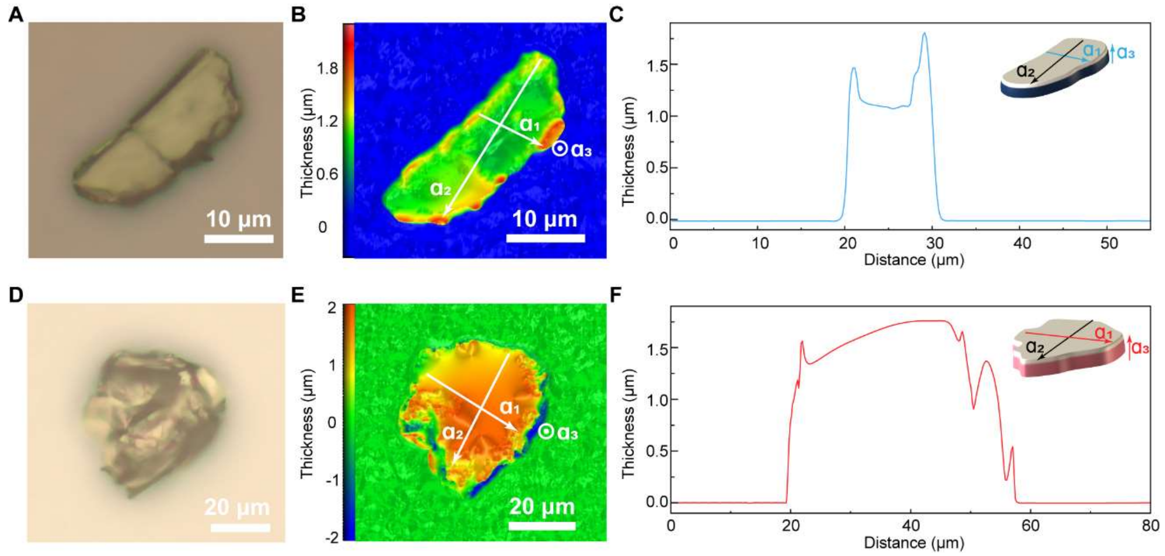

Fig. S2. Optical microscope images and three-dimensional (3D) profiles, which include lateral dimensions (a_1 and a_2) and thicknesses (a_3) of fillers, were measured using a Zygo's 3D optical profiler. To achieve statistical aspect ratios ($\frac{a_3}{a_1}$) of fillers, 3D profiles of both "perfect" fillers (graphite) and defective fillers (graphite oxide) were measured in 384 pieces of "perfect" fillers and 570 pieces of defective fillers. The single piece of defective filler (graphite oxide) in Fig. S2B and the single piece of "perfect" filler (graphite) in Fig. S2E serve as examples of how we measured lateral dimensions (a_1 and a_2) and thicknesses (a_3) of fillers. **(A)** Optical microscope image of a defective filler (graphite oxide). **(B)** A 3D profile, including lateral dimensions (a_1 and a_2) and thickness (a_3) of a defective filler (graphite oxide) in Fig. S2A measured by a Zygo's 3D optical profiler. **(C)** Thickness profile of the defective filler (graphite oxide) (a_3) in Fig. S2B along the a_1 direction measured by a Zygo's 3D optical profiler. **(D)** Optical microscope image of a "perfect" filler (graphite). **(E)** A 3D profile, including lateral dimensions (a_1 and a_2) and thickness (a_3) of a "perfect" filler (graphite) in Fig. S2D measured by a Zygo's 3D optical profiler. **(F)** Thickness profile of the "perfect" filler (graphite) (a_3) in Fig. S2D along a_1 direction measured by a Zygo's 3D optical profiler.

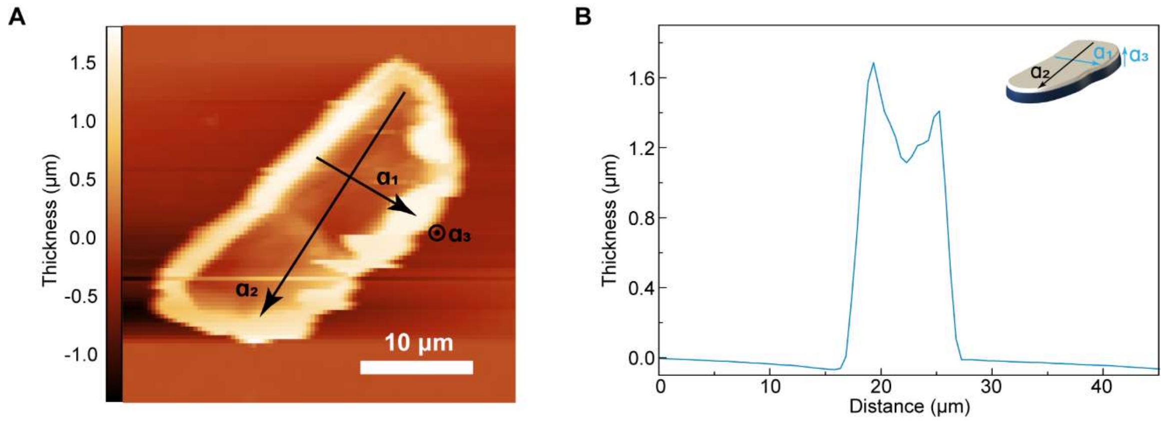

Fig. S3. The lateral size and thickness of the filler measured using atomic force microscopy. The results in Fig. S3 agree well with those measured using Zygo's three-dimensional optical profiler, as shown in Fig. S2B and Fig. S2C. (A) A three-dimensional profile, including lateral dimensions (a_1 and a_2) and thickness (a_3) of a defective filler (graphite oxide) in Fig. S2A was measured by an atomic force microscope. **(B)** Thickness profile of the defective filler (graphite oxide) (a_3) in Fig. S3A along a_1 direction measured by AFM. These results from the atomic force microscope confirm that the results measured by a Zygo's three-dimensional optical profiler in Fig. S2C are accurate.

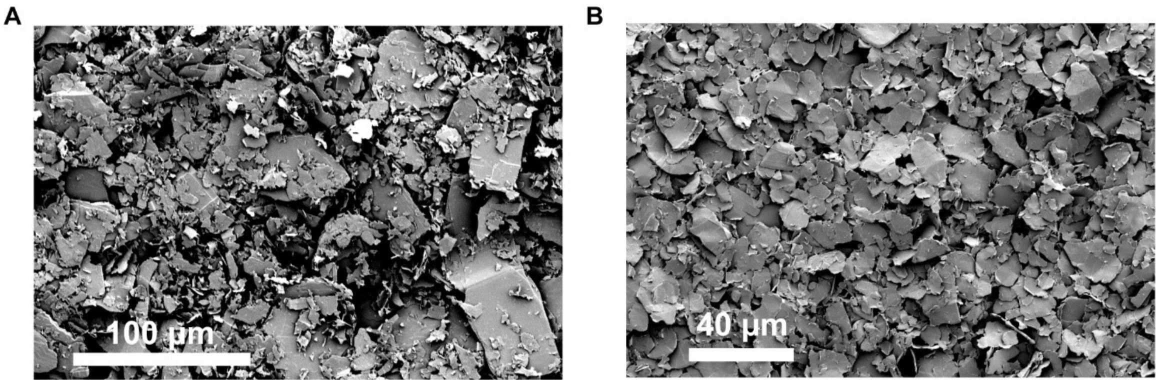

Fig. S4. The scanning electron microscope (SEM) images of the fillers. These images further corroborated the lateral size of the fillers displayed in Figs. S1, S2, and S3. **(A)** Scanning electron microscope (SEM) images of “perfect” fillers (graphite) with a scale bar of 100 μm. **(B)** SEM images of defective fillers (graphite oxide) with a scale bar of 40 μm.

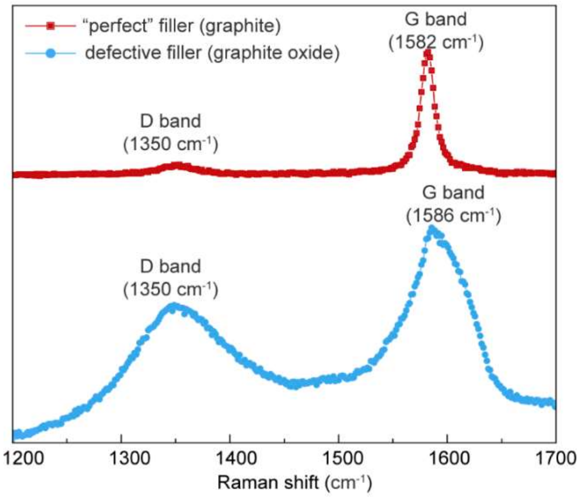

Fig. S5. Raman spectra of the "perfect" fillers (graphite) and the defective fillers (graphite oxide). The D-band is referred to as the defect band or disordered band.

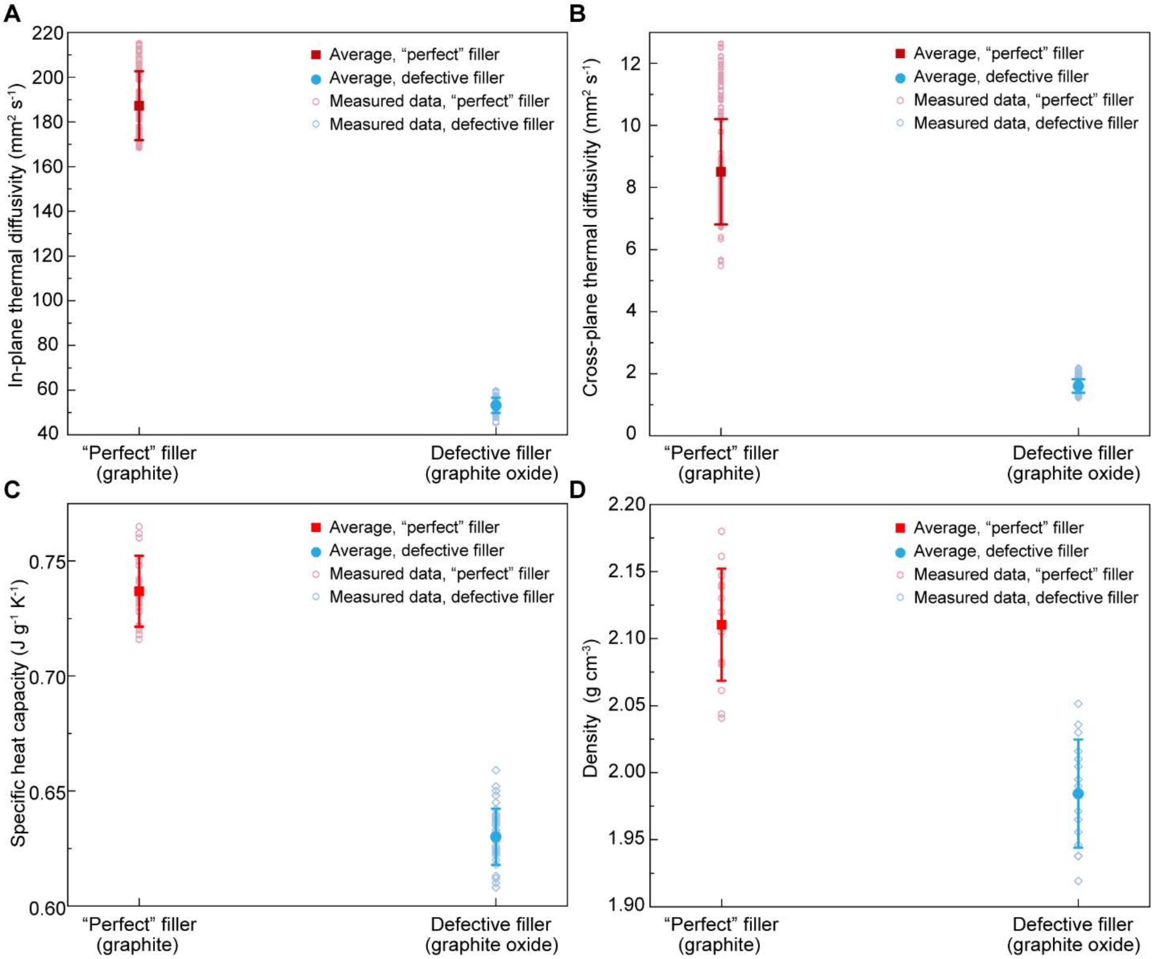

Fig. S6. Measured in-plane thermal diffusivities, specific heat capacities, and densities of fillers. **(A)** Measured in-plane thermal diffusivities of compressed pellets made from “perfect” fillers (graphite) or defective fillers (graphite oxide) at 25 °C. For details of sample preparations and thermal diffusivity measurements, please refer to the experimental section in the supplementary materials. The error bars in the figure are the population standard deviation. The error bars of the in-plane thermal diffusivities of the fillers are based on error propagation from the measured in-plane thermal diffusivities obtained from 15 samples across 5 batches. **(B)** Measured cross-plane thermal diffusivities of compressed pellets made from “perfect” fillers (graphite) or defective fillers (graphite oxide) at 25 °C. For details of sample preparation and thermal diffusivity measurements, please refer to the experimental section in the supplementary materials. The error bars in the figure are the population standard deviation. The error bars of the cross-plane thermal diffusivities of the fillers are based on error propagation from the measured cross-plane thermal diffusivity obtained from 12 samples across 5 batches. We note that the measured cross-plane thermal diffusivities of compressed pellets made from “perfect” fillers (graphite) could be higher than those of the referenced graphite (*93*). This could be due to the fact that the “perfect” fillers (graphite, e.g., $\pi-\pi$ stacking direction in graphite) in our compressed pellets may not be perfectly aligned along the k_{33}^* direction, as shown in Fig. S7 below. **(C)** Measured specific heat capacities of “perfect” fillers (graphite) and defective fillers (graphite oxide) at 25 °C. For details of differential scanning calorimetry measurements, please refer to the experimental section in the supplementary materials. The error bars of the specific heat capacities

of the "perfect" fillers (graphite) are based on measured specific heat capacity obtained from 9 samples across 5 batches. The error bars of the specific heat capacities of the defective fillers (graphite oxide) are based on measured specific heat capacity obtained from 15 samples across 5 batches. **(D)** Measured densities of "perfect" fillers (graphite) and defective fillers (graphite oxide) at 25 °C. The densities are calculated by measuring the thicknesses and weights of the pressed pellet of the fillers ("perfect" fillers (graphite) or defective fillers). The error bars in the figure are the population standard deviation. The error bars in the figure are the population standard deviation. The error bars of the densities of the pressed pellet of fillers are based on error propagation from the measured density obtained from 15 samples across 5 batches.

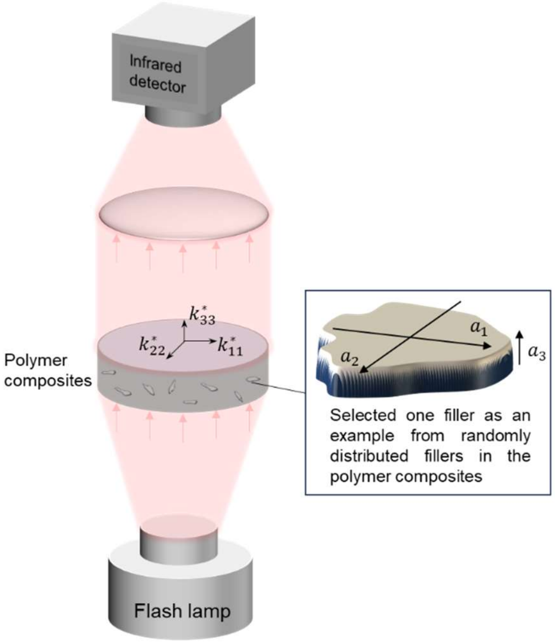

Fig. S7. Schematic illustration of thermal diffusivity measurements in "perfect" fillers (graphite), defective fillers (graphite oxide), polymer (PVA) films, and polymer/filler composites, conducted using the laser flash method. One filler was selected as an example from the randomly distributed fillers in the polymer composites. The lateral dimensions/sizes (a_1 and a_2) and thickness (a_3) of the selected filler are specified.

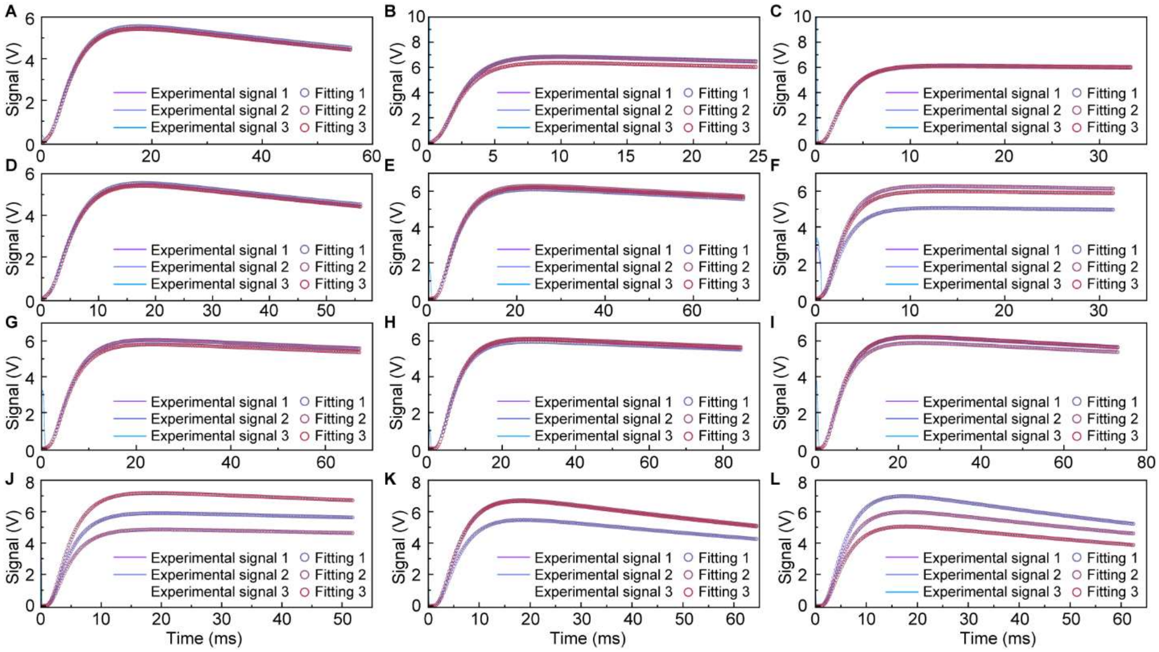

Fig. S8. Measured cross-plane thermal diffusivities of compressed pellets made from defective fillers (graphite oxide). To minimize random errors and ensure the reproducibility of thermal diffusivity measurements using the laser flash method, we tested the thermal diffusivities of each sample three times. Representative thermal diffusivity results of 12 different pressed pellets of fillers (defective fillers (graphite oxide)) were shown. We labeled as sample No.1, No.2, No.3, No.4, No.5, No.6, No.7, No.8, and No.9 which were sprayed with graphite coatings (DGF 123) on both sides before thermal diffusivity testing. We labeled as sample No.10, No.11, and No.12, which were not sprayed with graphite coatings (DGF 123) on both sides before thermal diffusivity testing. The "penetration model" in the LFA 467 software was used to fit the cross-plane thermal diffusivity experimental signals obtained from the laser flash method. **(A)** The experimental and fitting results in cross-plane thermal diffusivities of a pressed pellet of fillers (defective fillers (graphite oxide)) (No.1) with thickness of 0.233 mm. **(B)** The experimental and fitting results in cross-plane thermal diffusivities of a pressed pellet of fillers (defective fillers (graphite oxide)) (No.2) with thickness of 0.138 mm. **(C)** The experimental and fitting results in cross-plane thermal diffusivities of a pressed pellet of fillers (defective fillers (graphite oxide)) (No.3) with thickness of 0.181 mm. **(D)** The experimental and fitting results in cross-plane thermal diffusivities of a pressed pellet of fillers (defective fillers (graphite oxide)) (No.4) with thickness of 0.250 mm. **(E)** The experimental and fitting results in cross-plane thermal diffusivities of a pressed pellet of fillers (defective fillers (graphite oxide)) (No.5) with thickness of 0.260 mm. **(F)** The experimental and fitting results in cross-plane thermal diffusivities of a pressed pellet of fillers (defective fillers (graphite oxide)) (No.6) with thickness of 0.171 mm. **(G)** The experimental and fitting results in cross-plane thermal diffusivities of a pressed pellet of fillers (defective fillers (graphite oxide)) (No.7) with thickness of 0.254 mm. **(H)** The experimental and fitting results in cross-plane thermal diffusivities of a pressed pellet of fillers (defective fillers (graphite oxide)) (No.8) with thickness of 0.281 mm. **(I)** The experimental and fitting results in cross-plane thermal diffusivities of a pressed pellet of fillers (defective fillers (graphite oxide)) (No.9) with thickness of 0.262 mm. **(J)** The experimental and fitting results in cross-plane thermal diffusivities of a pressed pellet of fillers (defective fillers (graphite oxide)) (No.10) with thickness of 0.246 mm. **(K)** The experimental and fitting results in cross-plane thermal diffusivities of a pressed pellet of

fillers (defective fillers (graphite oxide)) (No.11) with thickness of 0.291 mm. **(L)** The experimental and fitting results in cross-plane thermal diffusivities of a pressed pellet of fillers (defective fillers (graphite oxide)) (No.12) with thickness of 0.284 mm. For details of sample preparations and thermal diffusivity measurements, please refer to the experimental section in the supplementary materials.

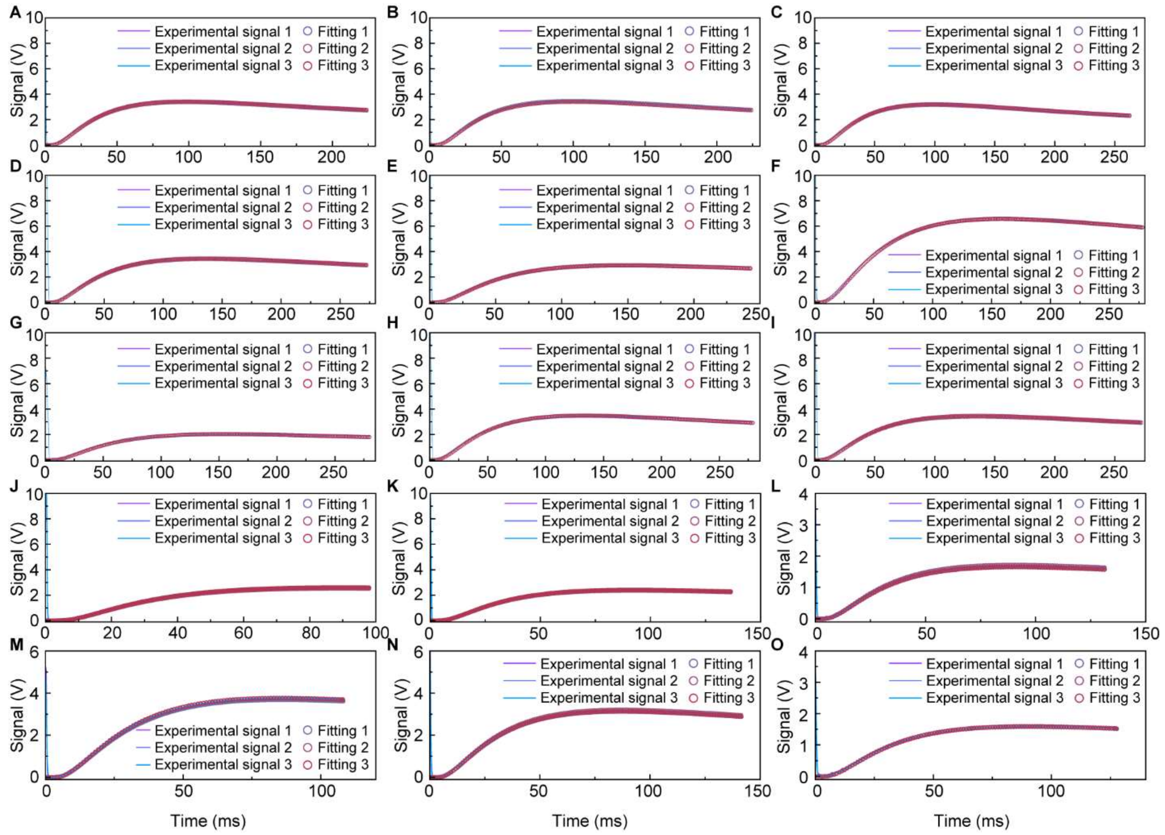

Fig. S9. Measured in-plane thermal diffusivities of compressed pellets made from defective fillers (graphite oxide). To minimize random errors and ensure the reproducibility of thermal diffusivity measurements using the laser flash method, we tested the thermal diffusivities of each sample three times. Representative thermal diffusivity results of 15 different pressed pellets of fillers (defective fillers (graphite oxide)) were shown. We labeled as sample No.1, No.2, No.3, No.4, No.5, No.6, No.7, No.8, and No.9, which were sprayed with graphite coatings (DGF 123) on both sides before thermal diffusivity testing. We labeled as sample No.10, No.11, No.12, No. 13, No. 14, and No. 15, which were not sprayed with graphite coatings (DGF 123) on both sides before thermal diffusivity testing. The "in-plane anisotropic model" in the LFA 467 software was used to fit the in-plane thermal diffusivity experimental signals obtained from the laser flash method. **(A)** The experimental and fitting results in in-plane thermal diffusivities of a pressed pellet of fillers (defective fillers (graphite oxide)) (No.1) with thickness of 0.195 mm. **(B)** The experimental and fitting results in in-plane thermal diffusivities of a pressed pellet of fillers (defective fillers (graphite oxide)) (No.2) with thickness of 0.153 mm. **(C)** The experimental and fitting results in in-plane thermal diffusivities of a pressed pellet of fillers (defective fillers (graphite oxide)) (No.3) with thickness of 0.236 mm. **(D)** The experimental and fitting results in in-plane thermal diffusivities of a pressed pellet of fillers (defective fillers (graphite oxide)) (No.4) with thickness of 0.263 mm. **(E)** The experimental and fitting results in in-plane thermal diffusivities of a pressed pellet of fillers (defective fillers (graphite oxide)) (No.5) with thickness of 0.203 mm. **(F)** The experimental and fitting results in in-plane thermal diffusivities of a pressed pellet of fillers (defective fillers (graphite oxide)) (No.6) with thickness of 0.212 mm. **(G)** The experimental and fitting results in in-plane thermal diffusivities of a pressed pellet of fillers (defective fillers (graphite oxide)) (No.7) with thickness of 0.214 mm. **(H)** The experimental and fitting results in in-plane thermal diffusivities of a pressed pellet of fillers (defective fillers

(graphite oxide)) (No.8) with thickness of 0.230 mm. **(I)** The experimental and fitting results in in-plane thermal diffusivities of a pressed pellet of fillers (defective fillers (graphite oxide)) (No.9) with thickness of 0.214 mm. **(J)** The experimental and fitting results in in-plane thermal diffusivities of a pressed pellet of fillers (defective fillers (graphite oxide)) (No.10) with thickness of 0.237 mm. **(K)** The experimental and fitting results in in-plane thermal diffusivities of a pressed pellet of fillers (defective fillers (graphite oxide)) (No.11) with thickness of 0.250 mm. **(L)** The experimental and fitting results in in-plane thermal diffusivities of a pressed pellet of fillers (defective fillers (graphite oxide)) (No.12) with thickness of 0.278 mm. **(M)** The experimental and fitting results in in-plane thermal diffusivities of a pressed pellet of fillers (defective fillers (graphite oxide)) (No.13) with thickness of 0.270 mm. **(N)** The experimental and fitting results in in-plane thermal diffusivities of a pressed pellet of fillers (defective fillers (graphite oxide)) (No.14) with thickness of 0.289 mm. **(O)** The experimental and fitting results in in-plane thermal diffusivities of a pressed pellet of fillers (defective fillers (graphite oxide)) (No.15) with thickness of 0.265 mm. For details of sample preparations and thermal diffusivity measurements, please refer to the experimental section in the supplementary materials.

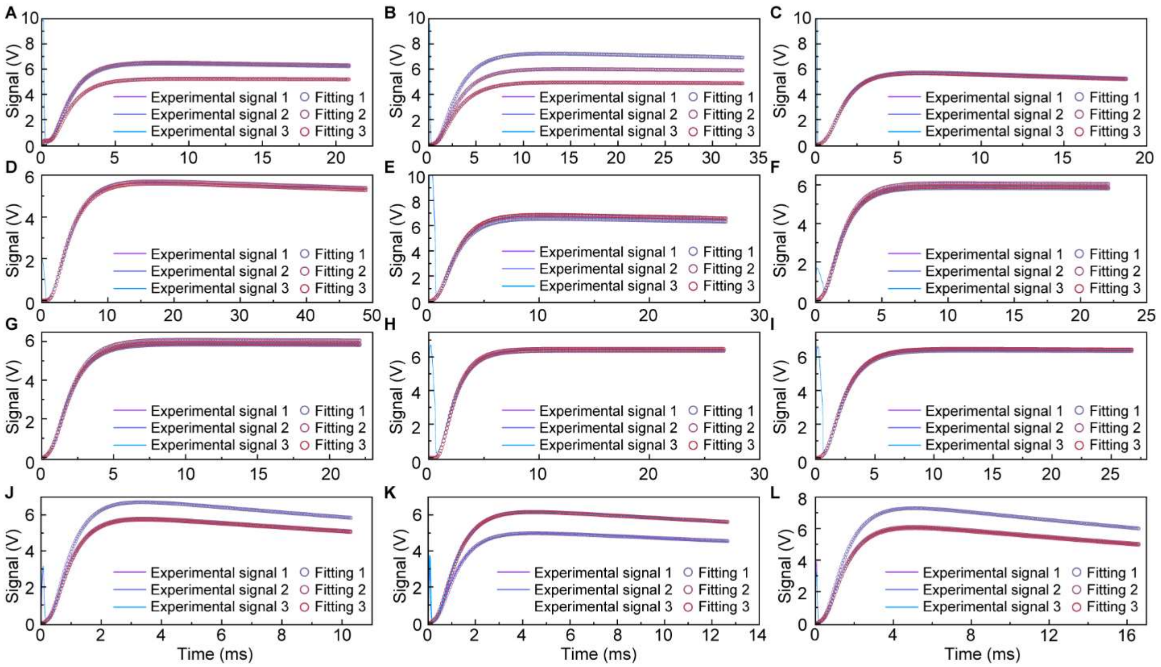

Fig. S10. Measured cross-plane thermal diffusivities of compressed pellets made from "perfect" fillers (graphite). To minimize random errors and ensure the reproducibility of thermal diffusivity measurements using the laser flash method, we tested the thermal diffusivities of each sample three times. Representative thermal diffusivity results of 12 different pressed pellets of fillers (the "perfect" fillers (graphite)) were shown. We labeled as sample No.1, No.2, No.3, No.4, No.5, No.6, No.7, No.8, and No.9, which were sprayed with graphite coatings (DGF 123) on both sides before thermal diffusivity testing. We labeled as sample No.10, No.11, and No.12, which were not sprayed with graphite coatings (DGF 123) on both sides before thermal diffusivity testing. The "penetration model" in the LFA 467 software was used to fit the cross-plane thermal diffusivity experimental signals obtained from the laser flash method. **(A)** The experimental and fitting results in cross-plane thermal diffusivities of a pressed pellet of fillers ("perfect" fillers (graphite)) (No.1) with thickness of 0.312 mm. **(B)** The experimental and fitting results in cross-plane thermal diffusivities of a pressed pellet of fillers ("perfect" fillers (graphite)) (No.2) with thickness of 0.399 mm. **(C)** The experimental and fitting results in cross-plane thermal diffusivities of a pressed pellet of fillers ("perfect" fillers (graphite)) (No.3) with thickness of 0.296 mm. **(D)** The experimental and fitting results in cross-plane thermal diffusivities of a pressed pellet of fillers ("perfect" fillers (graphite)) (No.4) with thickness of 0.462 mm. **(E)** The experimental and fitting results in cross-plane thermal diffusivities of a pressed pellet of fillers ("perfect" fillers (graphite)) (No.5) with thickness of 0.362 mm. **(F)** The experimental and fitting results in cross-plane thermal diffusivities of a pressed pellet of fillers ("perfect" fillers (graphite)) (No.6) with thickness of 0.316 mm. **(G)** The experimental and fitting results in cross-plane thermal diffusivities of a pressed pellet of fillers ("perfect" fillers (graphite)) (No.7) with thickness of 0.327 mm. **(H)** The experimental and fitting results in cross-plane thermal diffusivities of a pressed pellet of fillers ("perfect" fillers (graphite)) (No.8) with thickness of 0.335 mm. **(I)** The experimental and fitting results in cross-plane thermal diffusivities of a pressed pellet of fillers ("perfect" fillers (graphite)) (No.9) with thickness of 0.345 mm. **(J)** The experimental and fitting results in cross-plane thermal diffusivities of a pressed pellet of fillers ("perfect" fillers (graphite)) (No.10) with thickness of 0.272 mm. **(K)** The experimental and fitting results in cross-plane thermal diffusivities of a pressed pellet of fillers

(“perfect” fillers (graphite)) (No.11) with thickness of 0.316 mm. **(L)** The experimental and fitting results in cross-plane thermal diffusivities of a pressed pellet of fillers (“perfect” fillers (graphite)) (No.12) with thickness of 0.362 mm. For details of sample preparations and thermal diffusivity measurements, please refer to the experimental section in the supplementary materials.

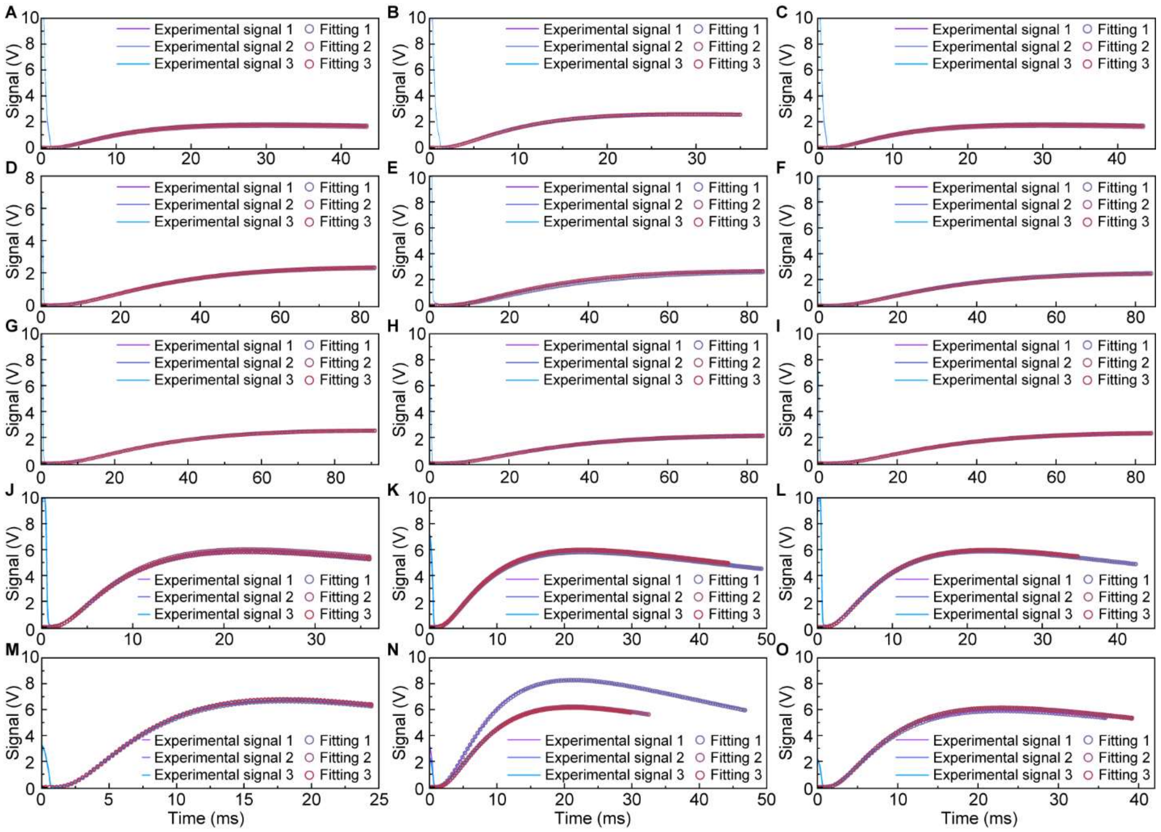

Fig. S11. Measured in-plane thermal diffusivities of compressed pellets made from "perfect" fillers (graphite). To minimize random errors and ensure the reproducibility of thermal diffusivity measurements using the laser flash method, we tested the thermal diffusivities of each sample three times. Representative thermal diffusivity results of 15 different pressed pellets of fillers (the "perfect" fillers (graphite)) were shown. We labeled as sample No.1, No.2, No.3, No.4, No.5, No.6, No.7, No.8, and No.9, which were sprayed with graphite coatings (DGF 123) on both sides before thermal diffusivity testing. We labeled as sample No.10, No.11, No.12, No. 13, No. 14, and No. 15 which were not sprayed with graphite coatings (DGF 123) on both sides before thermal diffusivity testing. The "in-plane anisotropic model" was used to fit the in-plane thermal diffusivity experimental signals obtained from the laser flash method. **(A)** The experimental and fitting results in in-plane thermal diffusivities of a pressed pellet of fillers ("perfect" fillers (graphite)) (No.1) with thickness of 0.463 mm. **(B)** The experimental and fitting results in in-plane thermal diffusivities of a pressed pellet of fillers ("perfect" fillers (graphite)) (No.2) with thickness of 0.296 mm. **(C)** The experimental and fitting results in in-plane thermal diffusivities of a pressed pellet of fillers ("perfect" fillers (graphite)) (No.3) with thickness of 0.381 mm. **(D)** The experimental and fitting results in in-plane thermal diffusivities of a pressed pellet of fillers ("perfect" fillers (graphite)) (No.4) with thickness of 0.277 mm. **(E)** The experimental and fitting results in in-plane thermal diffusivities of a pressed pellet of fillers ("perfect" fillers (graphite)) (No.5) with thickness of 0.254 mm. **(F)** The experimental and fitting results in in-plane thermal diffusivities of a pressed pellet of fillers ("perfect" fillers (graphite)) (No.6) with thickness of 0.226 mm. **(G)** The experimental and fitting results in in-plane thermal diffusivities of a pressed pellet of fillers ("perfect" fillers (graphite)) (No.7) with thickness of 0.261 mm. **(H)** The experimental and fitting results in in-plane thermal diffusivities of a pressed pellet of fillers ("perfect" fillers (graphite)) (No.8) with thickness of 0.279 mm. **(I)** The experimental and fitting results in in-plane thermal

diffusivities of a pressed pellet of fillers (“perfect” fillers (graphite)) (No.9) with thickness of 0.246 mm. **(J)** The experimental and fitting results in in-plane thermal diffusivities of a pressed pellet of fillers (“perfect” fillers (graphite)) (No.10) with thickness of 0.278 mm. **(K)** The experimental and fitting results in in-plane thermal diffusivities of a pressed pellet of fillers (“perfect” fillers (graphite)) (No.11) with thickness of 0.333 mm. **(L)** The experimental and fitting results in in-plane thermal diffusivities of a pressed pellet of fillers (“perfect” fillers (graphite)) (No.12) with thickness of 0.334 mm. **(M)** The experimental and fitting results in in-plane thermal diffusivities of a pressed pellet of fillers (“perfect” fillers (graphite)) (No.13) with thickness of 0.235 mm. **(N)** The experimental and fitting results in in-plane thermal diffusivities of a pressed pellet of fillers (“perfect” fillers (graphite)) (No.14) with thickness of 0.275 mm. **(O)** The experimental and fitting results in in-plane thermal diffusivities of a pressed pellet of fillers (“perfect” fillers (graphite)) (No.15) with thickness of 0.291 mm. For details of sample preparations and thermal diffusivity measurements, please refer to the experimental section in the supplementary materials.

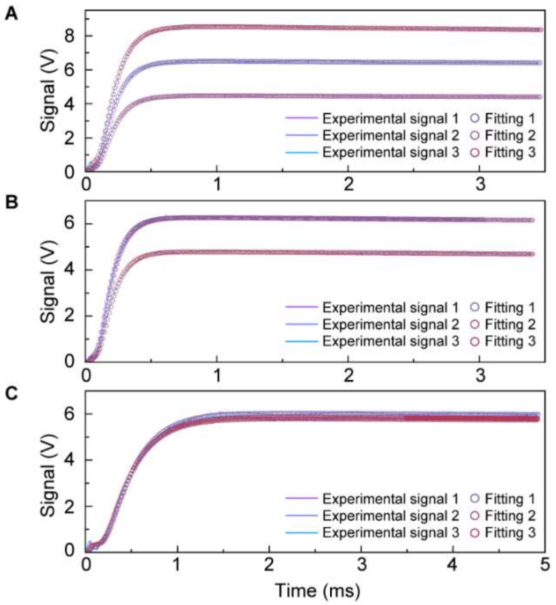

Fig. S12. Measured cross-plane thermal diffusivities of PVA films. To minimize random errors and ensure the reproducibility of thermal diffusivity measurements using the laser flash method, we tested the thermal diffusivities of each sample three times. Representative thermal diffusivity results of three different PVA film samples were shown. We labeled as sample No.1, No.2, and No.3. The "transparent model" in the LFA 467 software was used to fit the experimental signals obtained from the laser flash method. **(A)** The experimental and fitting results in thermal diffusivity of PVA film (No.1) with a thickness of 0.014 mm. **(B)** The experimental and fitting results in thermal diffusivity of PVA film (No.2) with a thickness of 0.013 mm. **(C)** The experimental and fitting results in thermal diffusivity of PVA film (No.3) with a thickness of 0.022 mm.

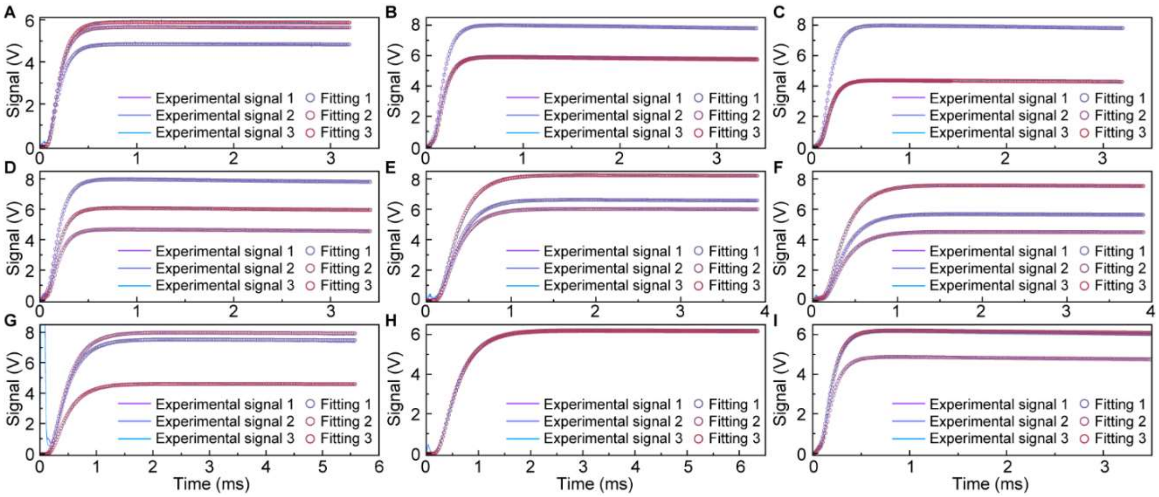

Fig. S13. Measured cross-plane thermal diffusivities of PVA/defective filler (graphite oxide, 1 vol%) composites. To minimize random errors and ensure the reproducibility of thermal diffusivity measurements using the laser flash method, we tested the thermal diffusivities of each sample (thin film) three times. Representative thermal diffusivity results of nine different PVA/defective filler (graphite oxide, 1 vol%) composites were shown. We labeled as sample No.1, No.2, No.3, No.4, No.5, No.6, No.7, No.8, and No.9. The "transparent model" in the LFA 467 software was used to fit the experimental signals obtained from the laser flash method. **(A)** The experimental and fitting results in thermal diffusivities of sample No.1 with a thickness of 0.015 mm. **(B)** The experimental and fitting results in thermal diffusivities of sample No.2 with a thickness of 0.016 mm. **(C)** The experimental and fitting results in thermal diffusivity of sample No.3 with a thickness of 0.015 mm. **(D)** The experimental and fitting results in thermal diffusivities of sample No.4 with a thickness of 0.017 mm. **(E)** The experimental and fitting results in thermal diffusivities of sample No.5 with a thickness of 0.014 mm. **(F)** The experimental and fitting results in thermal diffusivities of sample No.6 with a thickness of 0.014 mm. **(G)** The experimental and fitting results in thermal diffusivities of sample No.7 with a thickness of 0.015 mm. **(H)** The experimental and fitting results in thermal diffusivities of sample No.8 with a thickness of 0.015 mm. **(I)** The experimental and fitting results in thermal diffusivities of sample No.9 with a thickness of 0.017 mm.

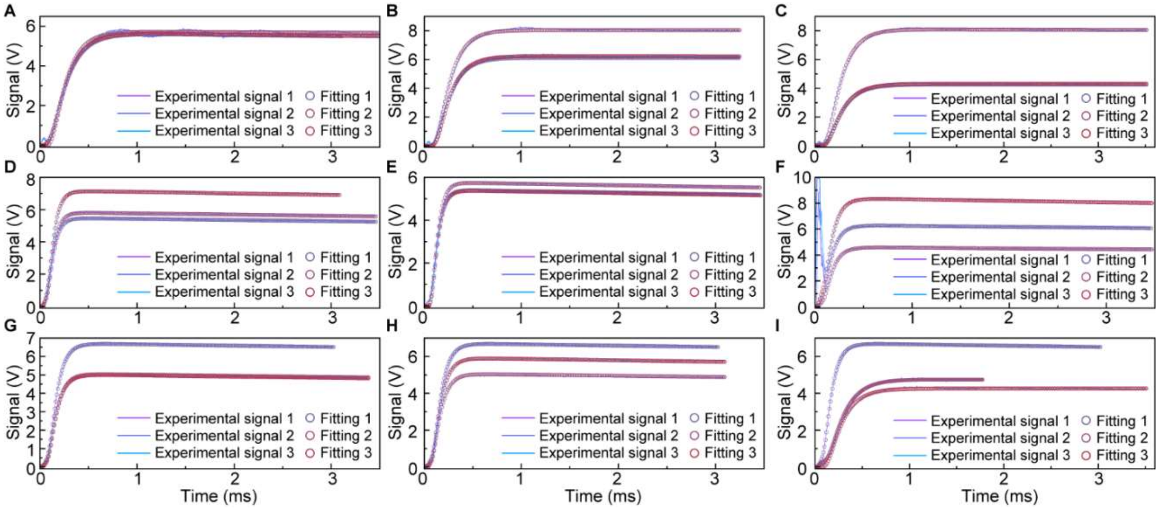

Fig. S14. Measured cross-plane thermal diffusivities of PVA/defective filler (graphite oxide, 3 vol%) composites. To minimize random errors and ensure the reproducibility of thermal diffusivity measurements using the laser flash method, we tested the thermal diffusivities of each sample (thin film) three times. Representative thermal diffusivity results of nine different PVA/defective filler (graphite oxide, 3 vol%) composites were shown. We labeled as sample No.1, No.2, No.3, No.4, No.5, No.6, No.7, No.8, and No.9. The "transparent model" in the LFA 467 software was used to fit the experimental signals obtained from the laser flash method. **(A)** The experimental and fitting results in thermal diffusivities of sample No.1 with a thickness of 0.025 mm. **(B)** The experimental and fitting results in thermal diffusivities of sample No.2 with a thickness of 0.024 mm. **(C)** The experimental and fitting results in thermal diffusivities of sample No.3 with a thickness of 0.025 mm. **(D)** The experimental and fitting results in thermal diffusivities of sample No.4 with a thickness of 0.016 mm. **(E)** The experimental and fitting results in thermal diffusivities of sample No.5 with a thickness of 0.016 mm. **(F)** The experimental and fitting results in thermal diffusivities of sample No.6 with a thickness of 0.018 mm. **(G)** The experimental and fitting results in thermal diffusivities of sample No.7 with a thickness of 0.021 mm. **(H)** The experimental and fitting results in thermal diffusivities of sample No.8 with a thickness of 0.017 mm. **(I)** The experimental and fitting results in thermal diffusivities of sample No.9 with a thickness of 0.015 mm.

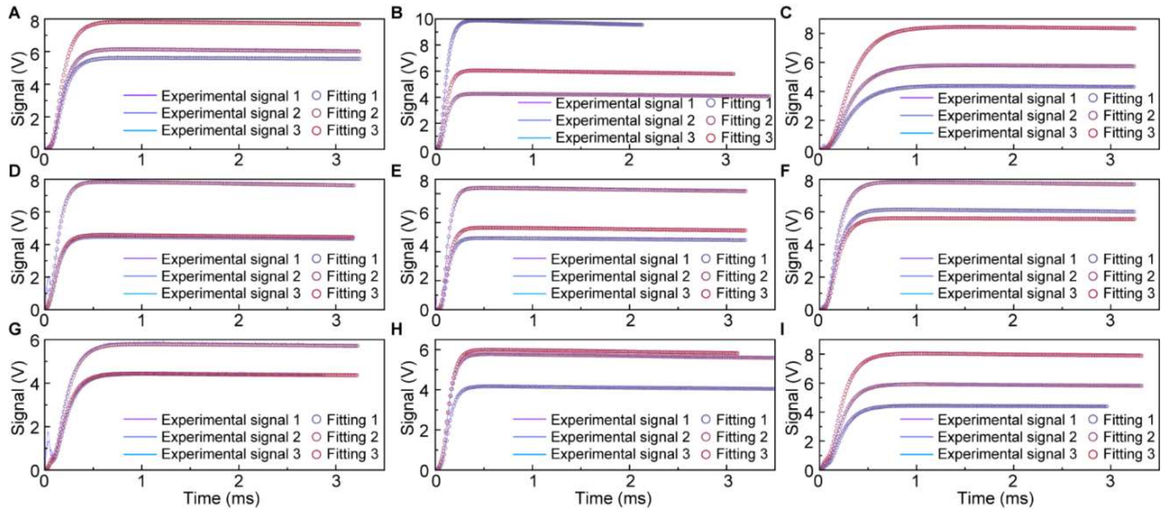

Fig. S15. Measured cross-plane thermal diffusivities of PVA/defective filler (graphite oxide, 5 vol%) composites. To minimize random errors and ensure the reproducibility of thermal diffusivity measurements using the laser flash method, we tested the thermal diffusivities of each sample (thin film) three times. Representative thermal diffusivity results of nine different PVA/defective filler (graphite oxide, 5 vol%) composites were shown. We labeled as sample No.1, No.2, No.3, No.4, No.5, No.6, No.7, No.8, and No.9. The "transparent model" in the LFA 467 software was used to fit the experimental signals obtained from the laser flash method. **(A)** The experimental and fitting results in thermal diffusivities of sample No.1 with a thickness of 0.030 mm. **(B)** The experimental and fitting results in thermal diffusivities of sample No.2 with a thickness of 0.019 mm. **(C)** The experimental and fitting results in thermal diffusivities of sample No.3 with a thickness of 0.031 mm. **(D)** The experimental and fitting results in thermal diffusivities of sample No.4 with a thickness of 0.028 mm. **(E)** The experimental and fitting results in thermal diffusivities of sample No.5 with thickness of 0.021 mm. **(F)** The experimental and fitting results in thermal diffusivities of sample No.6 with thickness of 0.029 mm. **(G)** The experimental and fitting results in thermal diffusivities of sample No.7 with a thickness of 0.031 mm. **(H)** The experimental and fitting results in thermal diffusivities of sample No.8 with a thickness of 0.023 mm. **(I)** The experimental and fitting results in thermal diffusivities of sample No.9 with a thickness of 0.032 mm.

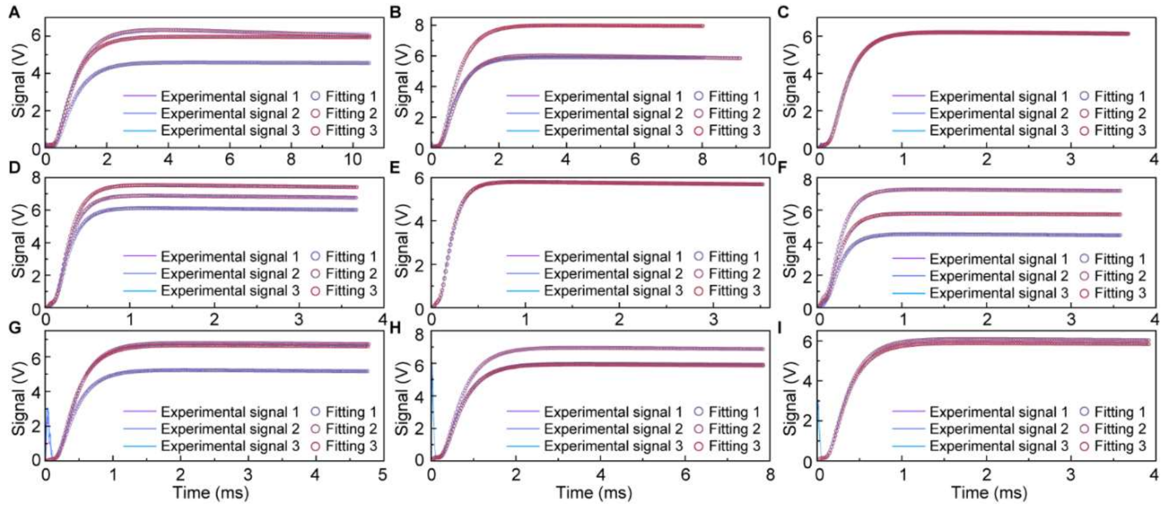

Fig. S16. Measured cross-plane thermal diffusivities of PVA/"perfect" filler (graphite, 1 vol%) composites. To minimize random errors and ensure the reproducibility of thermal diffusivity measurements using the laser flash method, we tested the thermal diffusivities of each sample (thin film) three times. Representative thermal diffusivity results of nine different PVA/"perfect" filler (graphite, 1 vol%) composites were shown. We labeled as sample No.1, No.2, No.3, No.4, No.5, No.6, No.7, No.8, and No.9. The "transparent model" in the LFA 467 software was used to fit the experimental signals obtained from the laser flash method. **(A)** The experimental and fitting results in thermal diffusivity of sample No.1 with thickness of 0.019 mm. **(B)** The experimental and fitting results in thermal diffusivity of sample No.2 with thickness of 0.018 mm. **(C)** The experimental and fitting results in thermal diffusivity of sample No.3 with thickness of 0.016 mm. **(D)** The experimental and fitting results in thermal diffusivity of sample No.4 with thickness of 0.034 mm. **(E)** The experimental and fitting results in thermal diffusivity of sample No.5 with thickness of 0.017 mm. **(F)** The experimental and fitting results in thermal diffusivity of sample No.6 with thickness of 0.029 mm. **(G)** The experimental and fitting results in thermal diffusivity of sample No.7 with thickness of 0.022 mm. **(H)** The experimental and fitting results in thermal diffusivity of sample No.8 with thickness of 0.028 mm. **(I)** The experimental and fitting results in thermal diffusivity of sample No.9 with thickness of 0.030 mm.

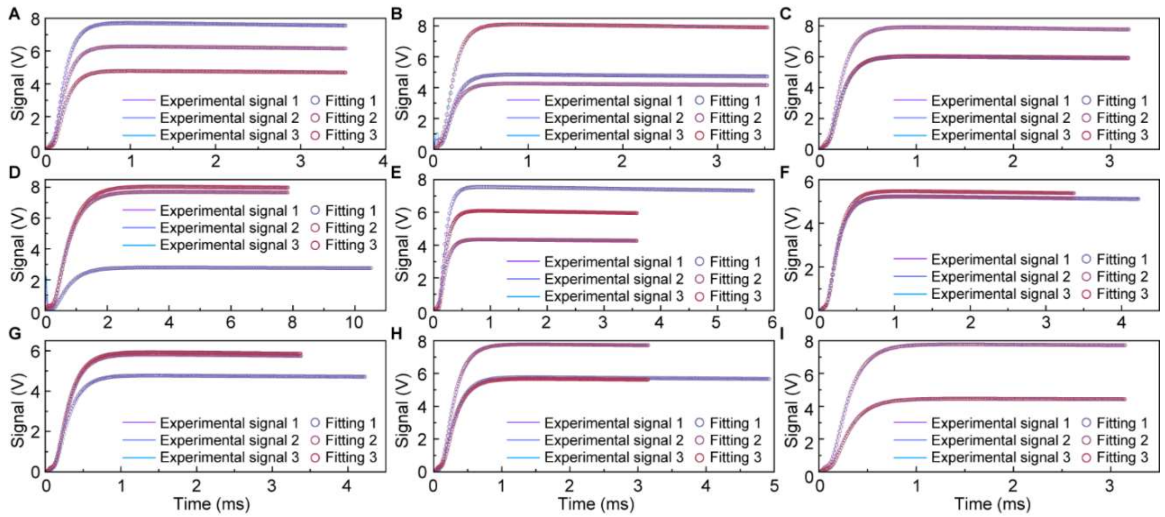

Fig. S17. Measured cross-plane thermal diffusivities of PVA/"perfect" filler (graphite, 3 vol%) composites. To minimize random errors and ensure the reproducibility of thermal diffusivity measurements using the laser flash method, we tested the thermal diffusivities of each sample (thin film) three times. Representative thermal diffusivity results of nine different PVA/"perfect" filler (graphite, 3 vol%) composites were shown. We labeled as sample No.1, No.2, No.3, No.4, No.5, No.6, No.7, No.8, and No.9. The "transparent model" in the LFA 467 software was used to fit the experimental signals obtained from the laser flash method. **(A)** The experimental and fitting results in thermal diffusivity of sample No.1 with thickness of 0.020 mm. **(B)** The experimental and fitting results in thermal diffusivity of sample No.2 with thickness of 0.020 mm. **(C)** The experimental and fitting results in thermal diffusivity of sample No.3 with thickness of 0.022 mm. **(D)** The experimental and fitting results in thermal diffusivity of sample No.4 with thickness of 0.036 mm. **(E)** The experimental and fitting results in thermal diffusivity of sample No.5 with thickness of 0.018 mm. **(F)** The experimental and fitting results in thermal diffusivity of sample No.6 with thickness of 0.020 mm. **(G)** The experimental and fitting results in thermal diffusivity of sample No.7 with thickness of 0.022 mm. **(H)** The experimental and fitting results in thermal diffusivity of sample No.8 with thickness of 0.023 mm. **(I)** The experimental and fitting results in thermal diffusivity of sample No.9 with thickness of 0.023 mm.

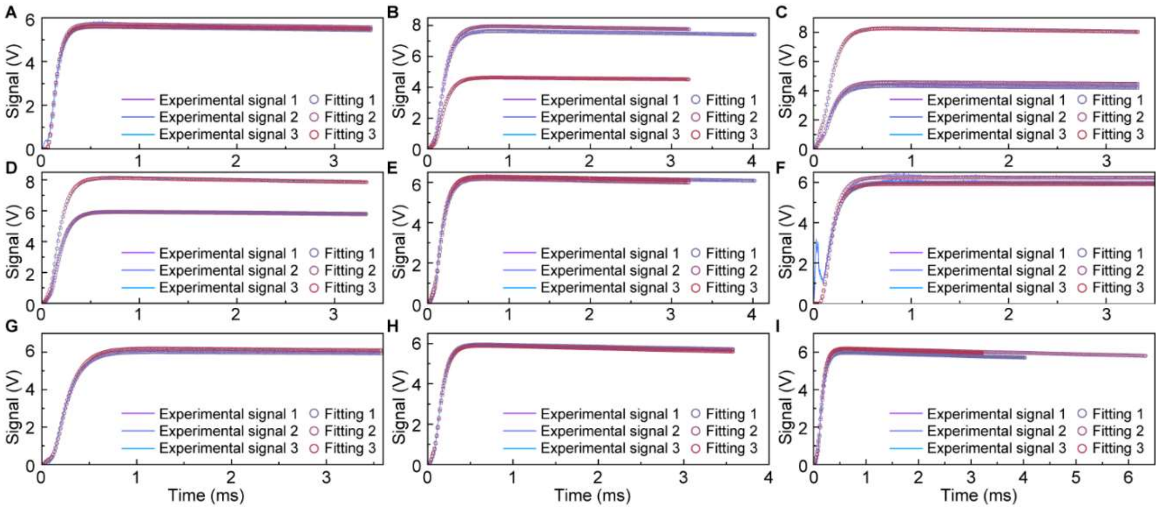

Fig. S18. Measured cross-plane thermal diffusivities of PVA/"perfect" filler (graphite, 5 vol%) composites. To minimize random errors and ensure the reproducibility of thermal diffusivity measurements using the laser flash method, we tested the thermal diffusivities of each sample (thin film) three times. Representative thermal diffusivity results of nine different PVA/"perfect" filler (graphite, 5 vol%) composites were shown. We labeled as sample No.1, No.2, No.3, No.4, No.5, No.6, No.7, No.8, and No.9. The "transparent model" in the LFA 467 software was used to fit the experimental signals obtained from the laser flash method. **(A)** The experimental and fitting results in thermal diffusivity of sample No.1 with thickness of 0.019 mm. **(B)** The experimental and fitting results in thermal diffusivity of sample No.2 with thickness of 0.021 mm. **(C)** The experimental and fitting results in thermal diffusivity of sample No.3 with thickness of 0.022 mm. **(D)** The experimental and fitting results in thermal diffusivity of sample No.4 with thickness of 0.022 mm. **(E)** The experimental and fitting results in thermal diffusivity of sample No.5 with thickness of 0.021 mm. **(F)** The experimental and fitting results in thermal diffusivity of sample No.6 with thickness of 0.022 mm. **(G)** The experimental and fitting results in thermal diffusivity of sample No.7 with thickness of 0.026 mm. **(H)** The experimental and fitting results in thermal diffusivity of sample No.8 with thickness of 0.020 mm. **(I)** The experimental and fitting results in thermal diffusivity of sample No.9 with thickness of 0.022 mm.

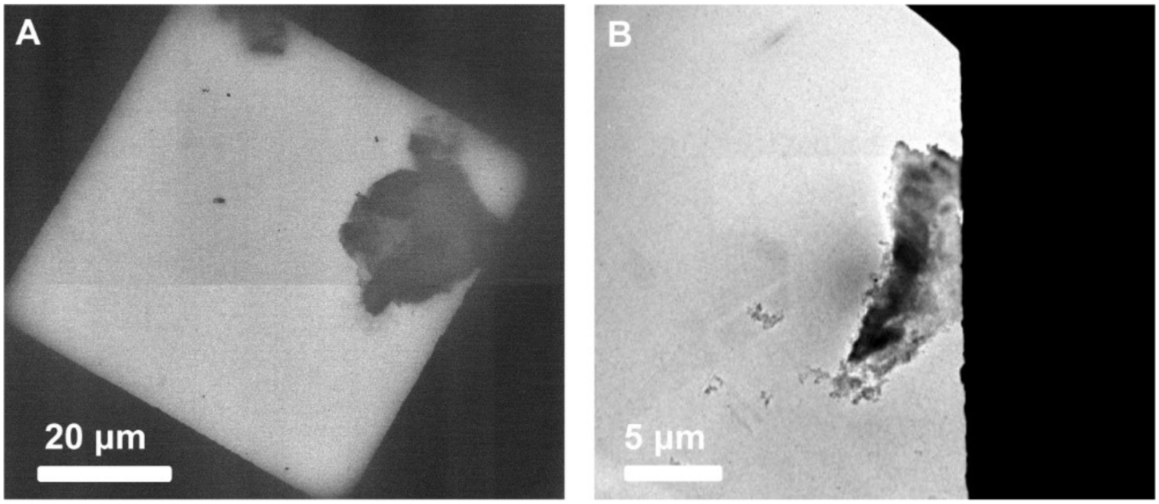

Fig. S19. Representative transmission electron microscopy (TEM) images of polymer composite thin films. (A) TEM images of PVA/defective filler (graphite oxide, 5 vol%) composites with filler at 5 vol% (volume fraction). **(B)** TEM images of PVA/“perfect” filler (graphite, 5 vol%) composites with filler at 5 vol%.

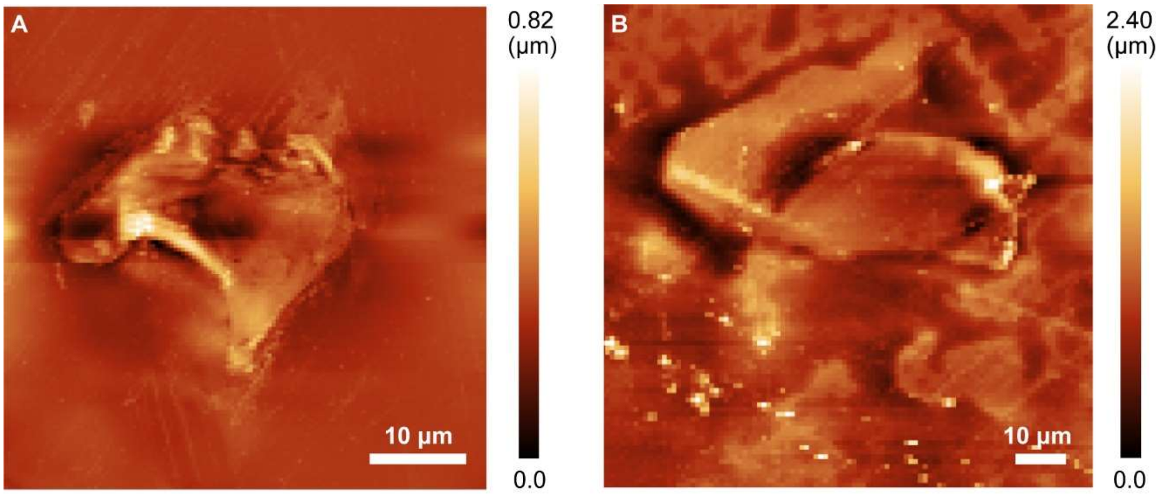

Fig. S20. Representative tapping-mode atomic force microscopy images of polymer composite films on glass substrates. The measurements were taken under normal atmospheric conditions at room temperature. **(A)** An atomic force microscopy image of a PVA/“perfect” filler (graphite) composites film shows the morphology and distribution of the fillers at 5 vol%. **(B)** An atomic force microscopy image of a PVA/defective filler (graphite oxide) composites film shows the morphology and distribution of the fillers at 5 vol%. The model numbers are Asylum MFP-3D and Bruker NCLV for the atomic force microscopy system and atomic force microscopy tip, respectively, in our lab.

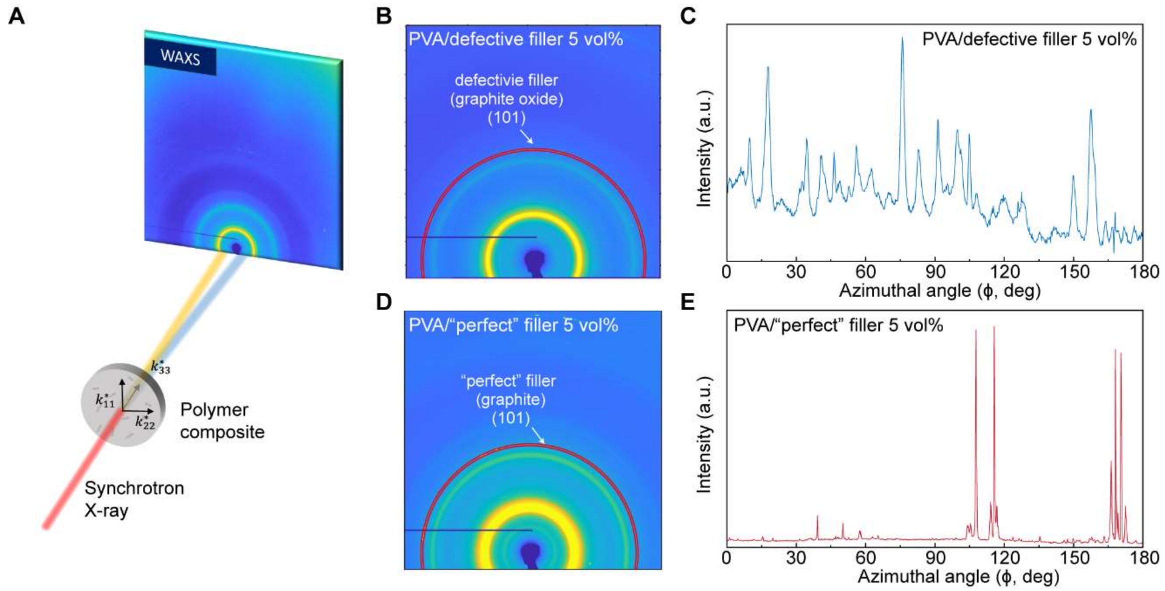

Fig. S21. Structural characterizations of polymer composites using synchrotron wide-angle X-ray scattering (WAXS). (A) Illustration of the experimental setup and a polymer composite film. **(B-E)** Figs. S21B and S21D depict the WAXS scattering patterns of PVA/defective filler (graphite oxide, 5 vol%) composites and PVA/“perfect” filler (graphite, 5 vol%) composites, respectively. Fig. S21C and Fig. S21E represent azimuthal line cuts integrated around the (101) peak ($q = 3.075$ Å^{-1}) within a ± 0.025 Å^{-1} width of the fillers (depicted as concentric red circles in Figs. S21B and S21D, respectively). Spikes on the curves in Figs. S21C and S21E indicate poorly dispersed crystallites. The baseline of the azimuthal intensity curves shows no clear angular dependence, suggesting that with the X-rays' resolving capability we did not detect orientational dependence. The calculated value is $\frac{1}{3}$ for the statistical orientations ($< \cos^2 \theta >$) of the fillers in PVA/defective filler (graphite oxide, 5 vol%) composites and PVA/“perfect” filler (graphite, 5 vol%) composites, using the measured data in Figs. 4B, 4C, and 4E.

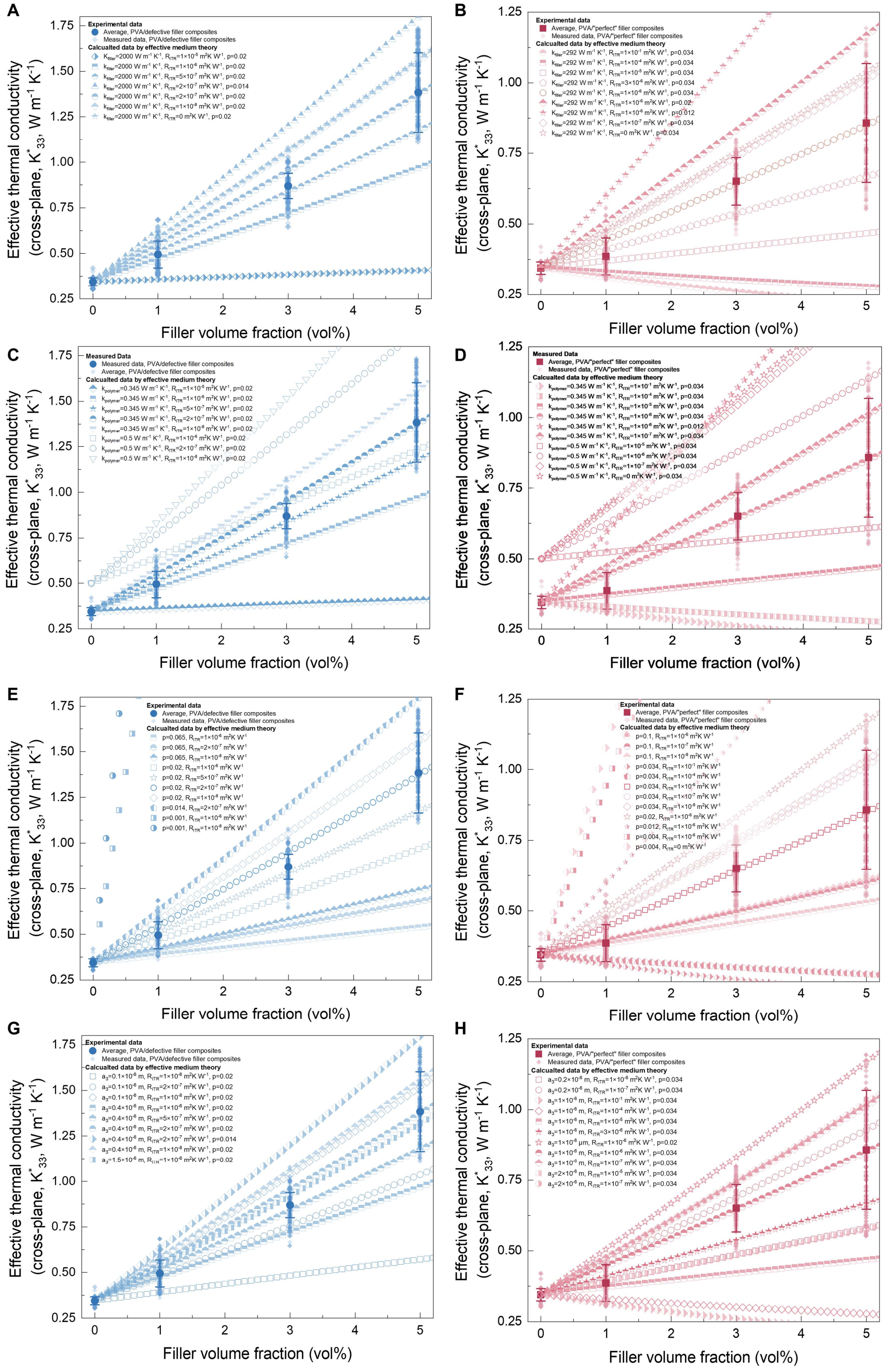

Fig. S22. Comparisons between thermal conductivities calculated using effective medium theory and measured thermal conductivities in PVA/filler composites in the cross-plane direction. Additional calculation details are available in supplementary materials Section S2. **(A)** The relationships between the measured thermal conductivities and the calculated thermal conductivities in the cross-plane direction (k_{33}^{*}), using different variables interfacial thermal resistances (R_{ITR}) and thermal conductivities of fillers (k_{filler}) in PVA/defective filler (graphite

oxide) composites, suggest the overestimated R_{ITR} in PVA/defective filler (graphite oxide, 5 vol%) composites at approximately $2 \times 10^{-7}\ m^2\ K\ W^{-1}$. **(B)** The relationships between the measured thermal conductivities and calculated k_{33}^*, employing different variables (R_{ITR} and k_{filler}) in PVA/"perfect" filler (graphite) composites, suggest an underestimated R_{ITR} in PVA/"perfect" filler (graphite, 5 vol%) composites at approximately $1 \times 10^{-6}\ m^2\ K\ W^{-1}$. **(C)** The relationships between the measured thermal conductivities and the calculated k_{33}^*, using different variables interfacial thermal resistances (R_{ITR}) and thermal conductivities of polymer matrices ($k_{polymer}$) in PVA/defective filler (graphite oxide) composites, suggest the overestimated R_{ITR} in PVA/defective filler (graphite oxide, 5 vol%) composites at approximately $2 \times 10^{-7}\ m^2\ K\ W^{-1}$. **(D)** The relationships between the measured thermal conductivities and the calculated k_{33}^*, using different variables (R_{ITR} and $k_{polymer}$) in PVA/"perfect" filler (graphite, 5 vol%) composites, suggest the underestimated R_{ITR} in PVA/defective filler (graphite oxide) composites at approximately $1 \times 10^{-6}\ m^2\ K\ W^{-1}$. **(E)** The relationships between the measured thermal conductivities and the calculated k_{33}^*, using different variables (interfacial thermal resistances (R_{ITR}) and aspect ratios of fillers (p)) in PVA/defective filler (graphite oxide) composites, suggest the overestimated R_{ITR} in PVA/defective filler (graphite oxide, 5 vol%) composites at approximately $2 \times 10^{-7}\ m^2\ K\ W^{-1}$. **(F)** The relationships between the measured thermal conductivity and the calculated k_{33}^*, using different variables (R_{ITR} and p) in PVA/"perfect" filler (graphite) composites, suggest the underestimated R_{ITR} in PVA/defective filler (graphite oxide, 5 vol%) composites at approximately $1 \times 10^{-6}\ m^2\ K\ W^{-1}$. **(G)** The relationships between the measured thermal conductivities and the calculated k_{33}^*, using different variables (interfacial thermal resistances (R_{ITR}) and thicknesses (a_3)) in PVA/defective filler (graphite oxide) composites, suggest the overestimated R_{ITR} in PVA/defective filler (graphite oxide) composites at approximately $2 \times 10^{-7}\ m^2\ K\ W^{-1}$. **(H)** The relationships between the measured thermal conductivity and the calculated k_{33}^*, using different variables (R_{ITR} and a_3) in PVA/"perfect" filler (graphite) composites, suggest the underestimated R_{ITR} in PVA/"perfect" filler (graphite) composites at approximately $1 \times 10^{-6}\ m^2\ K\ W^{-1}$. Comparing our measured thermal conductivities with calculated thermal conductivities using effective medium theory (Fig. 5, Fig. S22, and section S2), the comparison results show that overestimated R_{ITR} in PVA/defective filler (graphite oxide, 5 vol%) composites ($2 \times 10^{-7}\ m^2\ K\ W^{-1}$) are lower than underestimated R_{ITR} in PVA/"perfect" filler (graphite, 5 vol%) composites ($1 \times 10^{-6}\ m^2\ K\ W^{-1}$).

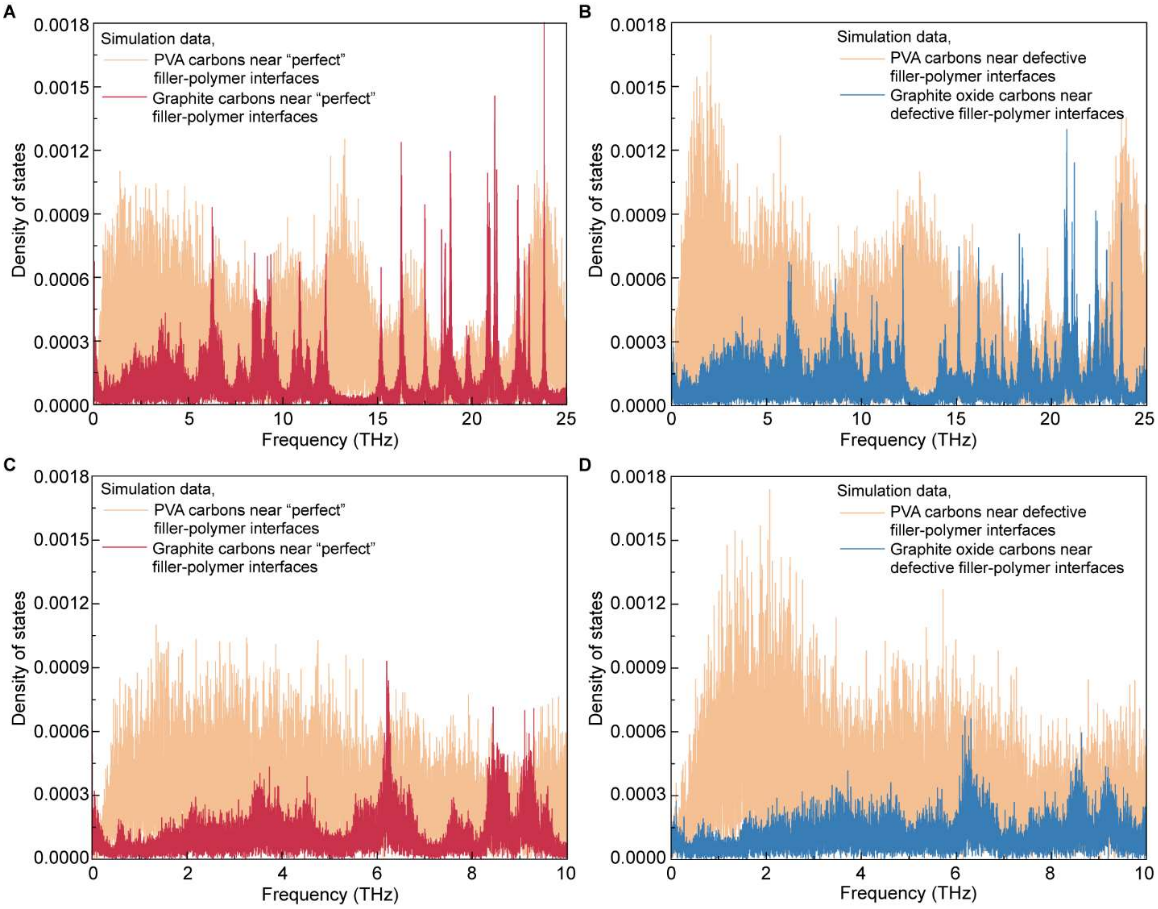

Fig. S23. The estimated density of states (DOS) by molecular dynamics simulation for carbon atoms in the filler and polymer near the polymer/filler interfaces in the composites. (A) DOS of "perfect" filler (graphite) carbon atoms and polymer (PVA) carbon atoms near the "perfect" filler-polymer interfaces in PVA/"perfect" filler (graphite) composites. **(B)** DOS of defective filler (graphite oxide) carbon atoms and polymer (PVA) carbon atoms near the defective filler-polymer interfaces in PVA/defective filler (graphite oxide) composites. The DOS results in Fig. S23A and Fig. S23B show sharper peaks for graphite carbons near the interface in the PVA/"perfect" filler (graphite) composites, compared to PVA/defective filler (graphite oxide) composites, up to a frequency of 25 THz. **(C)-(D)** The DOS up to 10 THz shows higher peaks at low frequencies for PVA carbons near the filler-polymer interface in the PVA/defective filler (graphite oxide) composites compared to the PVA/"perfect" filler (graphite) composites.

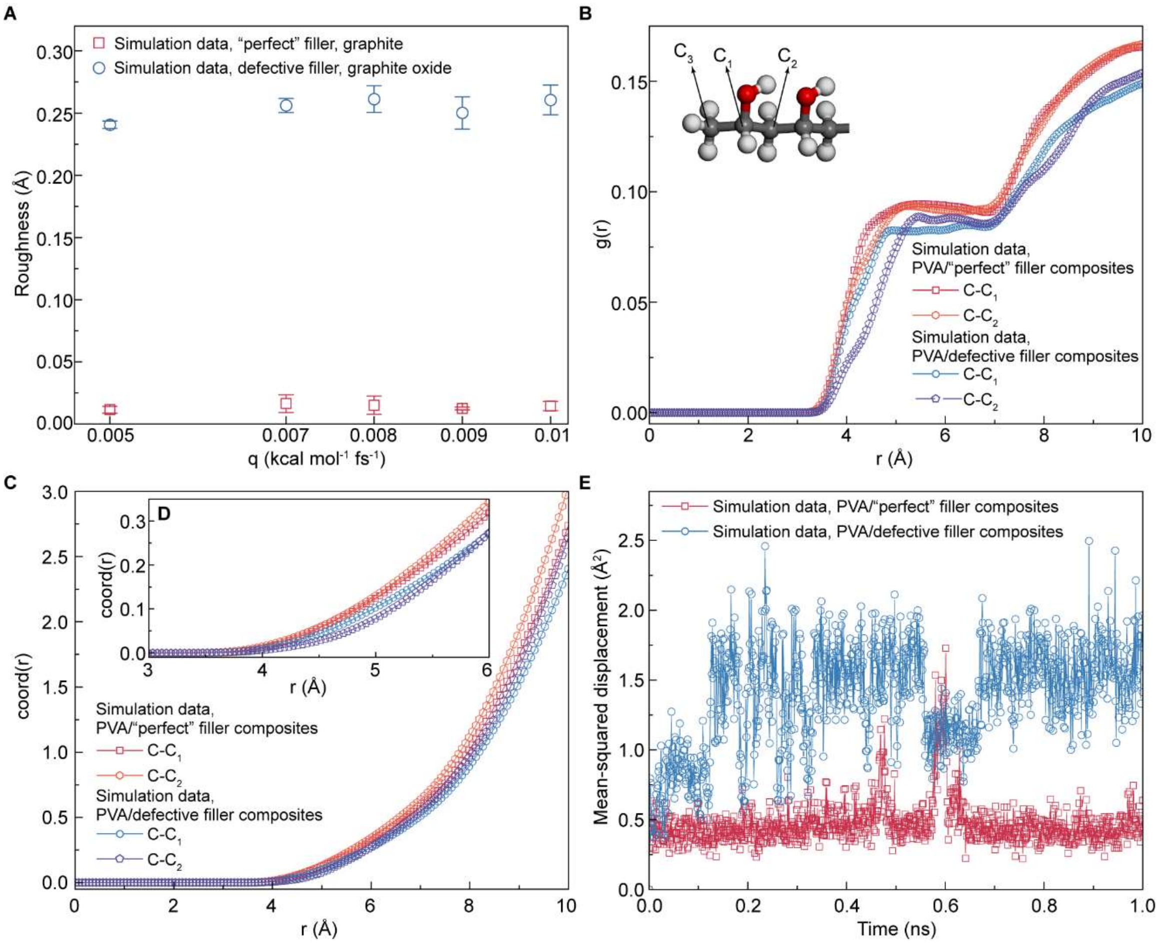

Fig. S24. Molecular dynamics simulation studies to examine the structures of the PVA/filler interface in composites. (A) The root-mean-square roughness of the top graphite layer at the graphite/PVA interface in PVA/"perfect" filler (graphite) composites and the root-mean-square roughness of the top graphite layer at the graphite oxide-PVA interface in PVA/defective filler (graphite oxide) composites differs significantly. The average roughness of the top graphite layer in PVA/defective filler (graphite oxide) composites is at least an order of magnitude higher than that in PVA/"perfect" filler (graphite) composites, due to the presence of functional groups (e.g., -OH) on the surface of defective fillers. The error bars represent the sample standard deviation. **(B)** Radial distribution function (RDF) of type C_1 and C_2 carbons in PVA with respect to graphite (or graphite oxide) carbons (represented as C). Graphite layers in graphite-oxide have an additional type of carbons which are connected to the -OH group. These carbons in graphite oxide were combined with the normal carbons in graphite oxide which are not connected to any -OH group for the calculation of RDF and coordination number (coord(r)) in PVA/defective filler (graphite oxide) composite. There are three types of carbon atoms in PVA. The carbon connected with three hydrogens is defined as type C_3, the carbon connected with two hydrogens is defined as type C_2, and the carbon connected with one hydrogen is defined as type C_1. The type C_3 carbon which is connected to three hydrogens is only one per chain. Therefore, the RDF and coordination number for type C_3 carbons are not shown. **(C-D)** Coordination number of type C_1 and C_2 carbons in PVA with respect to graphite (or graphite oxide) carbons (represented as C). **(E)** The mean-squared displacement (MSD) of PVA carbons near the PVA/filler interface was analyzed for both PVA/"perfect" filler (graphite) composites and PVA/defective filler (graphite oxide) composites.

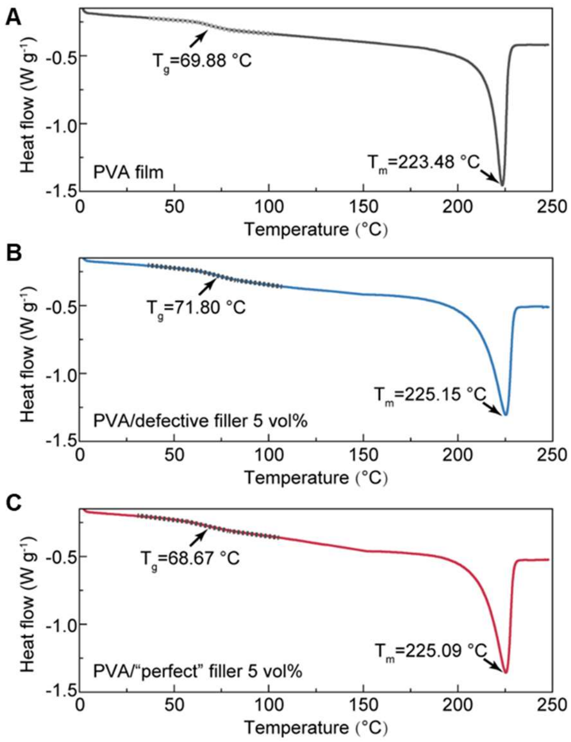

Fig. S25. Glass transition temperatures (T_g) and melting temperatures (T_m) of samples. (A) PVA, **(B)** PVA/defective filler (graphite oxide, 5 vol%) composites, and **(C)** PVA/"perfect" filler (graphite, 5 vol%) composites, measured using differential scanning calorimetry.

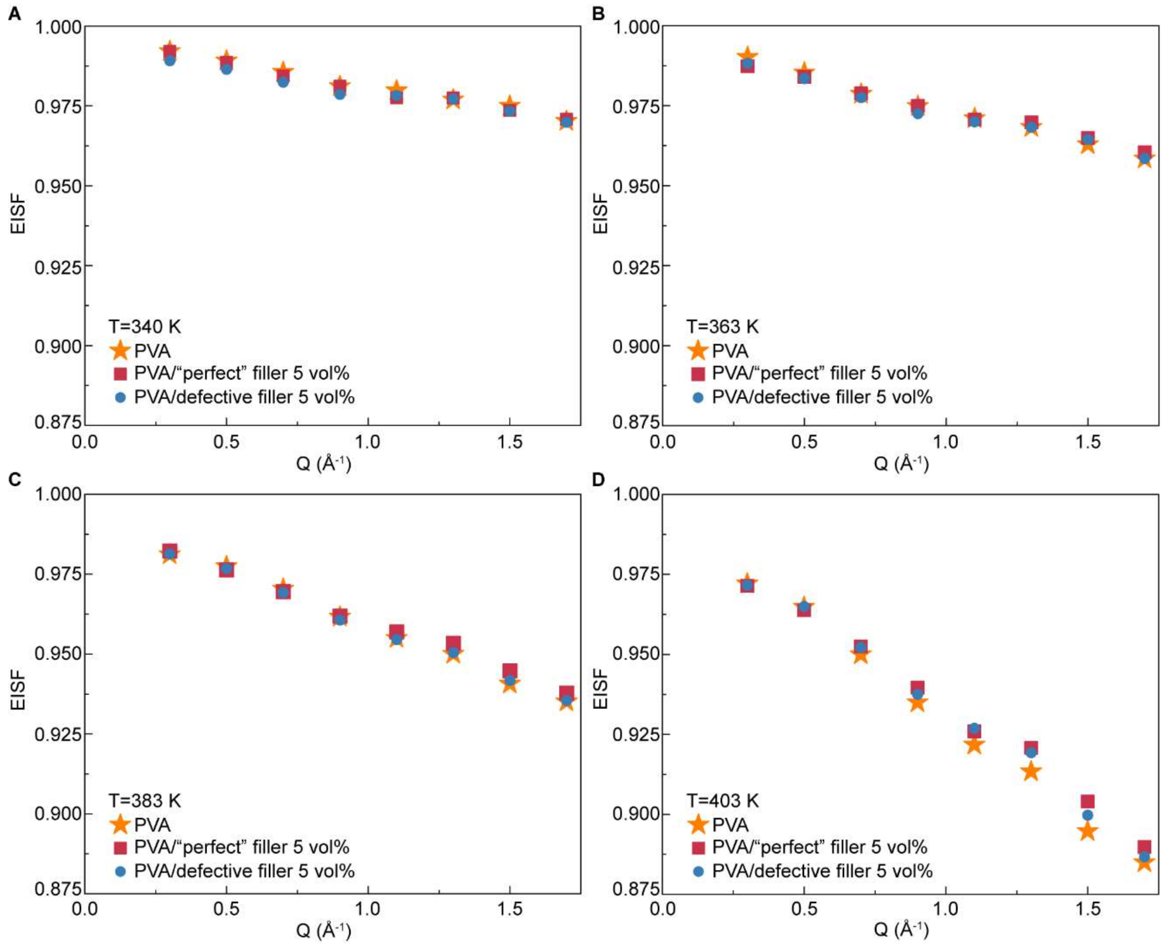

Fig. S26. Elastic incoherent structure factor (EISF) obtained from the analysis of the QENS spectra of PVA, PVA/“perfect” filler (graphite, 5 vol%), and PVA/defective filler (graphite oxide, 5 vol%) measured at various temperatures: (A) 340 K, **(B)** 363 K, **(C)** 383 K, **(D)** 403K.

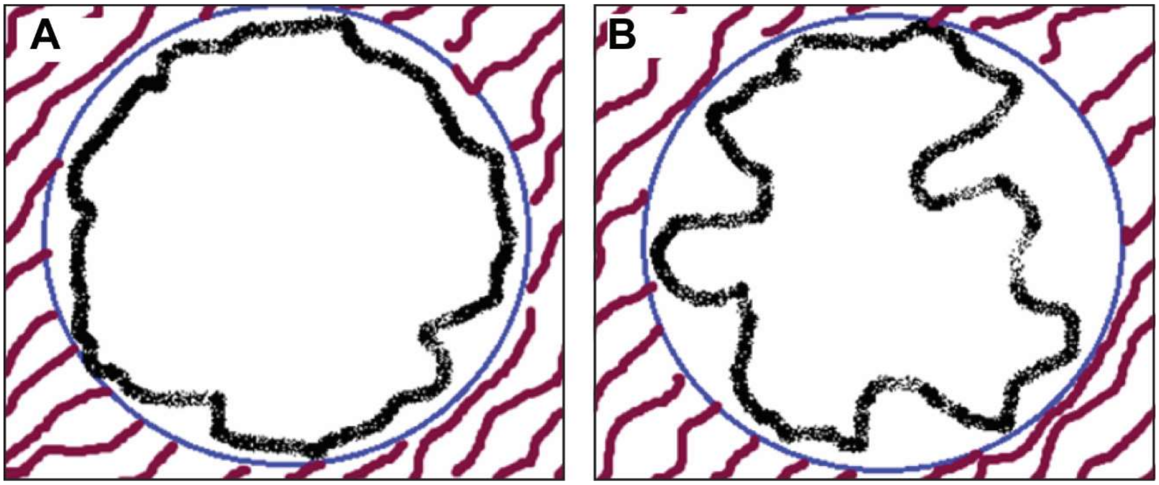

Fig. S27. A schematic of polymer chains arranged around the filler at the polymer/filler interface in composites. (A) Schematic of PVA chains arranged around a graphite filler in PVA/“perfect” filler (graphite, 5 vol%) composites. **(B)** Schematic of PVA chains arranged around a graphite oxide filler in PVA/defective filler (graphite oxide, 5 vol%). Lines with dark red color represent PVA molecules, and black color represents the surface of the fillers. The blue circle around the particles indicates the area inaccessible for the PVA molecules due to the filler’s roughness.

Table S1. The values of the parameters used in the numerical analysis (simple mechanical model) (*65*). This table has the adjustable parameters and physical constants used to accommodate a broader scope.

physical constants	ω_k	$5.07 \times 10^{-21}\, J$
physical constants	T	290
Physical constants	$k_b T$	$4.02 \times 10^{-12}\, J$
physical constants	V_{12}	0
adjustable parameters	$\frac{\omega_1}{\omega_k}$	
adjustable parameters	$\frac{V_k}{\omega_k}$	
adjustable parameters	O^+	0.1*omega
adjustable parameters	n_i	

Where ω_k represents the typical vibrational frequency within the polymer chains through bonded interactions, and is estimated from a previous study on polyvinyl alcohol and graphene oxide interfaces (*65*). There, by fitting the potential energy profile with respect to the atomic displacement, an effective force constant ranging from 23000 to 39000 $kJ\, mol^{-1}\, nm^{-2}$ can be obtained (*65*). This is then converted to the fundamental vibrational frequency based on the mass of carbon and hydrogen atoms in the polymer chain. ω_k mentioned in this table is the same as the $\omega_{\mathbf{q}}$ in the equation 2 in the main manuscript. n_i represents the number of defects per unit volume. The temperature (T) was set at 290 K for the numerical analysis. As a result, $k_b T$ is equal to $4.02 \times 10^{-12}\, J$, where k_b is the Bolzman constant.

V_{12} is taken as zero to emphasize that our model focuses on the vibrational coupling between PVA and graphite fillers.

This leaves only three adjustable parameters, including $\frac{\omega_1}{\omega_k}$, $\frac{V_k}{\omega_k}$, O^+, and n_i.

We solved the equations in Section 3.1 of this supplementary materials by adjusting the relevant adjustable parameters using the experimental thermal transport data from Fig. 2 in the main text. Through systematic tuning, we found that the values of $\frac{\omega_1}{\omega_k}$, $\frac{V_k}{\omega_k}$, O^+, and n_i fall within the specific ranges [0.08, 0.5], [0.08, 1.4], 0.1 $\times\omega$, and [25, 50], respectively. These values provide a good solution to the equations in Section 3.1.

We would like to point out that the regions presented in this figure depend strongly on the type of materials and their vibrational coupling strength. To justify whether the ranges of parameters we obtained for region II are reasonable, we evaluate the $\frac{\omega_1}{\omega_k}$ and $\frac{V_k}{\omega_k}$ using the specific materials in our study. Firstly, using the Debye temperature of pristine graphite in the c-axis, 450 K, we can estimate the dominant vibrational energy in graphite as $6.21 \times 10^{-21}\, J$. Therefore, the ratio of

$\frac{\omega_1}{\omega_k}$=1.22. Considering the defective graphite dominant vibrational energy should be smaller than the perfect graphite, as we can see from the vibrational density of states analysis, this ratio could be even smaller, so the current range of [0.08,1.4] for $\frac{\omega_1}{\omega_k}$ is reasonable. Secondly, we estimated the interfacial coupling energy between the perfect graphite and PVA is about 5% of the total energy of PVA, using molecular dynamics simulations. We also show that the interfacial vibrational coupling is stronger between the defective graphite and PVA, so we believe the range of [0.08,0.5] is reasonable for $\frac{V_k}{\omega_k}$ in the system of defective graphite filler in PVA.

REFERENCES AND NOTES

1. M. Peplow, The plastics revolution: How chemists are pushing polymers to new limits. *Nature* **536**, 266–268 (2016).

2. A. Dodabalapur, Organic and polymer transistors for electronics. *Mater. Today* **9**, 24–30 (2006).

3. T. Someya, Z. Bao, G. G. Malliaras, The rise of plastic bioelectronics. *Nature* **540**, 379–385 (2016).

4. K. K. Fu, J. Cheng, T. Li, L. Hu, Flexible batteries: From mechanics to devices. *ACS Energy Lett.* **1**, 1065–1079 (2016).

5. T. M. Swager, 50th anniversary perspective: Conducting/semiconducting conjugated polymers. A personal perspective on the past and the future. *Macromolecules* **50**, 4867–4886 (2017).

6. J. Chen, Y. Zhou, X. Huang, C. Yu, D. Han, A. Wang, Y. Zhu, K. Shi, Q. Kang, P. Li, P. Jiang, X. Qian, H. Bao, S. Li, G. Wu, X. Zhu, Q. Wang, Ladderphane copolymers for high-temperature capacitive energy storage. *Nature* **615**, 62–66 (2023).

7. E. Pop, Energy dissipation and transport in nanoscale devices. *Nano Res.* **3**, 147–169 (2010).

8. D. G. Cahill, W. K. Ford, K. E. Goodson, G. D. Mahan, A. Majumdar, H. J. Maris, R. Merlin, S. R. Phillpot, Nanoscale thermal transport. *J. Appl. Phys.* **93**, 793–818 (2002).

9. G. Chen, *Nanoscale energy transport and conversion: a parallel treatment of electrons*, *molecules*, *phonons*, *and photons* (Oxford Univ. Press, 2005).

10. A. L. Moore, L. Shi, Emerging challenges and materials for thermal management of electronics. *Mater. Today* **17**, 163–174 (2014).

11. Y. Guo, Y. Zhou, Y. Xu, Engineering polymers with metal-like thermal conductivity—Present status and future perspectives. *Polymer* **233**, 124168 (2021).

12. M. Goel, M. Thelakkat, Polymer thermoelectrics: Opportunities and challenges. *Macromolecules* **53**, 3632–3642 (2020).

13. S. Chen, A. L. Moore, W. Cai, J. W. Suk, J. An, C. Mishra, C. Amos, C. W. Magnuson, J. Kang, L. Shi, R. S. Ruoff, Raman measurements of thermal transport in suspended monolayer graphene of variable sizes in vacuum and gaseous environments. *ACS Nano* **5**, 321–328 (2011).

14. A. A. Balandin, Thermal properties of graphene and nanostructured carbon materials. *Nat. Mater.* **10**, 569–581 (2011).

15. Z. Han, A. Fina, Thermal conductivity of carbon nanotubes and their polymer nanocomposites: A review. *Prog. Polym. Sci.* **36**, 914–944 (2011).

16. Y. Xu, X. Wang, Q. Hao, A mini review on thermally conductive polymers and polymer-based composites. *Compos. Commun.* **24**, 100617 (2021).

17. B. Zhang, P. Mao, Y. Liang, Y. He, W. Liu, Z. Liu, Modulating thermal transport in polymers and interfaces: Theories, simulations, and experiments. *ES Energy Environ.* **5**, 37–55 (2019).

18. J. Chen, X. Xu, J. Zhou, B. Li, Interfacial thermal resistance: Past, present, and future. *Rev. Mod. Phys.* **94**, 025002 (2022).

19. P. Zhang, P. Yuan, X. Jiang, S. Zhai, J. Zeng, Y. Xian, H. Qin, D. Yang, A theoretical review on interfacial thermal transport at the nanoscale. *Small* **14**, 1702769 (2018).

20. A. R. J. Hussain, A. A. Alahyari, S. A. Eastman, C. Thibaud-Erkey, S. Johnston, M. J. Sobkowicz, Review of polymers for heat exchanger applications: factors concerning thermal conductivity. *Appl. Therm. Eng.* **113**, 1118–1127 (2017).

21. X. Zhang, G. Yang, B. Cao, Bonding-enhanced interfacial thermal transport: Mechanisms, materials, and applications. *Adv. Mater. Interfaces* **9**, 2200078 (2022).

22. P. E. Hopkins, Thermal transport across solid interfaces with nanoscale imperfections: Effects of roughness, disorder, dislocations, and bonding on thermal boundary conductance. *ISRN Mech. Eng.* **2013**, 682586 (2013).

23. L. H. Sperling, *Introduction to Physical Polymer Science* (Wiley, 2005).

24. R. Hanus, R. Gurunathan, L. Lindsay, M. T. Agne, J. Shi, S. Graham, G. Jeffrey Snyder, Thermal transport in defective and disordered materials. *Appl. Phys. Rev.* **8**, 031311 (2021).

25. X. Qian, J. Zhou, G. Chen, Phonon-engineered extreme thermal conductivity materials. *Nat. Mater.* **20**, 1188–1202 (2021).

26. E. T. Swartz, R. O. Pohl, Thermal boundary resistance. *Rev. Mod. Phys.* **61**, 605–668 (1989).

27. P. E. Hopkins, L. M. Phinney, J. R. Serrano, T. E. Beechem, Effects of surface roughness and oxide layer on the thermal boundary conductance at aluminum/silicon interfaces. *Phys. Rev. B* **82**, 085307 (2010).

28. K. Gordiz, A. Henry, Phonon transport at interfaces between different phases of silicon and germanium. *J. Appl. Phys.* **121**, 025102 (2017).

29. A. Giri, P. E. Hopkins, J. G. Wessel, J. C. Duda, Kapitza resistance and the thermal conductivity of amorphous superlattices. *J. Appl. Phys.* **118**, 165303 (2015).

30. A. Giri, S. W. King, W. A. Lanford, A. B. Mei, D. Merrill, L. Li, R. Oviedo, J. Richards, D. H. Olson, J. L. Braun, J. T. Gaskins, F. Deangelis, A. Henry, P. E. Hopkins, Interfacial defect vibrations enhance thermal transport in amorphous multilayers with ultrahigh thermal boundary conductance. *Adv. Mater.* **30**, e1804097 (2018).

31. M. D. Losego, M. E. Grady, N. R. Sottos, D. G. Cahill, P. V. Braun, Effects of chemical bonding on heat transport across interfaces. *Nat. Mater.* **11**, 502–506 (2012).

32. H. Zhang, A. F. Fonseca, K. Cho, Tailoring thermal transport property of graphene through oxygen functionalization. *J. Phys. Chem. C* **118**, 1436–1442 (2014).

33. A. Giri, P. E. Hopkins, A review of experimental and computational advances in thermal boundary conductance and nanoscale thermal transport across solid interfaces. *Adv. Funct. Mater.* **30**, 1903857 (2020).

34. W. S. Hummers Jr., R. E. Offeman, Preparation of graphitic oxide. *J. Am. Chem. Soc.* **80**, 1339–1339 (1958).

35. A. C. Ferrari, Raman spectroscopy of graphene and graphite: Disorder, electron–phonon coupling, doping and nonadiabatic effects. *Solid State Commun.* **143**, 47–57 (2007).

36. A. C. Ferrari, J. Robertson, Interpretation of Raman spectra of disordered and amorphous carbon. *Phys. Rev. B* **61**, 14095–14107 (2000).

37. M. A. Pimenta, G. Dresselhaus, M. S. Dresselhaus, L. G. Cançado, A. Jorio, R. Saito, Studying disorder in graphite-based systems by Raman spectroscopy. *Phys. Chem. Chem. Phys.* **9**, 1276–1290 (2007).

38. D. López-Díaz, M. López Holgado, J. L. García-Fierro, M. M. Velázquez, Evolution of the Raman spectrum with the chemical composition of graphene oxide. *J. Phys. Chem. C* **121**, 20489–20497 (2017).

39. I. B. Mason, R. H. Knibbs, Influence of crystallite size on the thermal conductivity of irradiated polycrystalline graphite. *Nature* **198**, 850–851 (1963).

40. H. W. Deem, W. D. Wood, Flash thermal-diffusivity measurements using a laser. *Rev. Sci. Instrum.* **33**, 1107–1109 (1962).

41. M. J. O'Neill, Measurement of specific heat functions by differential scanning calorimetry. *Anal. Chem.* **38**, 1331–1336 (1966).

42. K. M. Razeeb, E. Dalton, G. L. W. Cross, A. J. Robinson, Present and future thermal interface materials for electronic devices. *Int. Mater. Rev.* **63**, 1–21 (2018).

43. K. M. F. Shahil, A. A. Balandin, Graphene–multilayer graphene nanocomposites as highly efficient thermal interface materials. *Nano Lett.* **12**, 861–867 (2012).

44. Y. Xu, X. Wang, J. Zhou, B. Song, Z. Jiang, E. M. Y. Lee, S. Huberman, K. K. Gleason, G. Chen, Molecular engineered conjugated polymer with high thermal conductivity. *Sci. Adv.* **4**, eaar3031 (2018).

45. M. Mermoux, Y. Chabre, A. Rousseau, FTIR and 13C NMR study of graphite oxide. *Carbon* **29**, 469–474 (1991).

46. L. Yang, W. Weng, X. Fei, L. Pan, X. Li, W. Xu, Z. Hu, M. Zhu, Revealing the interrelation between hydrogen bonds and interfaces in graphene/PVA composites towards highly electrical conductivity. *Chem. Eng. J.* **383**, 123126 (2020).

47. H. S. Mansur, R. L. Oréfice, A. A. Mansur, Characterization of poly (vinyl alcohol)/poly (ethylene glycol) hydrogels and PVA-derived hybrids by small-angle X-ray scattering and FTIR spectroscopy. *Polymer* **45**, 7193–7202 (2004).

48. Y. He, B. Zhu, Y. Inoue, Hydrogen bonds in polymer blends. *Prog. Polym. Sci.* **29**, 1021–1051 (2004).

49. P. Hobza, Z. Havlas, Blue-shifting hydrogen bonds. *Chem. Rev.* **100**, 4253–4264 (2000).

50. B. Reif, S. E. Ashbrook, L. Emsley, M. Hong, Solid-state NMR spectroscopy. *Nat. Rev. Method Prime.* **1**, 2 (2021).

51. K. Masuda, H. Kaji, F. Horii, Solid-state ^{13}C NMR and ^{1}H CRAMPS investigations of the hydration process and hydrogen bonding for poly(vinyl alcohol) films. *Polyym. J.* **33**, 356–363 (2001).

52. M. Kobayashi, I. Ando, T. Ishii, S. Amiya, Structural study of poly(vinyl alcohol) in the gel state by high-resolution solid-state 13C NMR spectroscopy. *Macromolecules* **28**, 6677–6679 (1995).

53. D. D. Laws, H.-M. L. Bitter, A. Jerschow, Solid-state NMR spectroscopic methods in chemistry. *Angew. Chem. Int. Ed. Engl.* **41**, 3096–3129 (2002).

54. M. J. Duer, *Introduction to solid–state NMR spectroscopy* (Wiley, 2005).

55. M. J. Duer, *Solid state NMR spectroscopy: principles and applications* (Wiley, 2008).

56. R. J. Roe, *Methods of X–ray and neutron scattering in polymer science* (Oxford Univ. Press, 2000).

57. H. E. Assender, A. H. Windle, Crystallinity in poly(vinyl alcohol). 1. An X-ray diffraction study of atactic PVOH. *Polymer* **39**, 4295–4302 (1998).

58. Z. Q. Li, C. J. Lu, Z. P. Xia, Y. Zhou, Z. Luo, X-ray diffraction patterns of graphite and turbostratic carbon. *Carbon* **45**, 1686–1695 (2007).

59. Y. Xu, D. Kraemer, B. Song, Z. Jiang, J. Zhou, J. Loomis, J. Wang, M. Li, H. Ghasemi, X. Huang, X. Li, G. Chen, Nanostructured polymer films with metal-like thermal conductivity. *Nat. Commun.* **10**, 1771 (2019).

60. C. Nan, R. Birringer, D. R. Clarke, H. Gleiter, Effective thermal conductivity of particulate composites with interfacial thermal resistance. *J. Appl. Phys.* **81**, 6692–6699 (1997).

61. M. Deutsch, Orientational order determination in liquid crystals by X-ray diffraction. *Phys. Rev. A* **44**, 8264–8270 (1991).

62. S. Ghosh, W. Bao, D. L. Nika, S. Subrina, E. P. Pokatilov, C. N. Lau, A. A. Balandin, Dimensional crossover of thermal transport in few-layer graphene. *Nat. Mater.* **9**, 555–558 (2010).

63. N. Wang, M. K. Samani, H. Li, L. Dong, Z. Zhang, P. Su, S. Chen, J. Chen, S. Huang, G. Yuan, Tailoring the thermal and mechanical properties of graphene film by structural engineering. *Small* **14**, e1801346 (2018).

64. R. K. Gupta, E. Kennel, K.-J. Kim, *Polymer Nanocomposites Handbook* (CRC Press, 2009).

65. C. Zhu, N. Wei, J. Zhao, Coarse-Grained potentials of poly (vinyl alcohol)/graphene oxide interfaces. *Macromolecules* **55**, 1104–1119 (2022).

66. P. W. Anderson, B. I. Halperin, C. M. Varma, Anomalous low-temperature thermal properties of glasses and spin glasses. *Philos. Mag. J. Theor. Exp. Appl. Phys.* **25**, 1–9 (1972).

67. M. Li, Y. Tsurimaki, Q. Meng, N. Andrejevic, Y. Zhu, G. D. Mahan, G. Chen, Theory of electron–phonon–dislon interacting system—Toward a quantized theory of dislocations. *New J. Phys.* **20**, 023010 (2018).

68. J. Rammer, *Quantum transport theory* (CRC Press, 2018).

69. W. Humphrey, A. Dalke, K. Schulten, VMD: Visual molecular dynamics. *J. Mol. Graph.* **14**, 33–38 (1996).

70. M. J. Yoo, H. B. Park, Effect of hydrogen peroxide on properties of graphene oxide in Hummers method. *Carbon* **141**, 515–522 (2019).

71. V. H. Pham, S. H. Hur, E. J. Kim, B. S. Kim, J. S. Chung, Highly efficient reduction of graphene oxide using ammonia borane. *Chem. Comm.* **49**, 6665–6667 (2013).

72. X. Mei, J. Ouyang, Ultrasonication-assisted ultrafast reduction of graphene oxide by zinc powder at room temperature. *Carbon* **49**, 5389–5397 (2011).

73. M. Lojka, B. Lochman, O. Jankovský, A. Jiříčková, Z. Sofer, D. Sedmidubský, Synthesis, composition, and properties of partially oxidized graphite oxides. *Materials* **12**, 2367 (2019).

74. A. Bannov, A. Manakhov, A. Shibaev, A. Ukhina, J. Polčák, E. Maksimovskii, Synthesis dynamics of graphite oxide. *Thermochim. Acta* **663**, 165–175 (2018).

75. M. F. Fay, T. Dresel, Applications of model-based transparent surface films analysis using coherence-scanning interferometry. *Opt. Eng.* **56**, 111709 (2017).

76. P. de Groot, X. C. de Lega, Angle-resolved three-dimensional analysis of surface films by coherence scanning interferometry. *Opt. Lett.* **32**, 1638–1640 (2007).

77. E. Mamontov, K. W. Herwig, A time-of-flight backscattering spectrometer at the spallation neutron source, BASIS. *Rev. Sci. Instrum.* **82**, 085109 (2011).

78. C. Mbonu, N. C. Osti, D. Wu, P. Akcora, Quasi-elastic neutron scattering study on dynamically asymmetric polymer blends. *J. Polym. Sci.* **62**, 4177–4185 (2024).

79. N. C. Osti, N. H. Jalarvo, E. Mamontov, Backscattering silicon spectrometer (BASIS): Sixteen years in advanced materials characterization. *Mater. Horiz.* **11**, 4535–4572 (2024).

80. G. Wypych, *Handbook of Polymers* (Elsevier, 2022).

81. J. E. Mark, *Physical Properties of Polymers Handbook* (Springer, 2007).

82. J. M. Luttinger, Theory of thermal transport coefficients. *Phys. Rev.* **135**, A1505–A1514 (1964).

83. S. Plimpton, Fast parallel algorithms for short-range molecular dynamics. *J. Comput. Phys.* **117**, 1–19 (1995).

84. A. P. Thompson, H. M. Aktulga, R. Berger, D. S. Bolintineanu, W. M. Brown, P. S. Crozier, P. J. In't Veld, A. Kohlmeyer, S. G. Moore, T. D. Nguyen, LAMMPS-a flexible simulation tool for particle-based materials modeling at the atomic, meso, and continuum scales. *Comput. Phys. Commun.* **271**, 108171 (2022).

85. H. Sun, Force field for computation of conformational energies, structures, and vibrational frequencies of aromatic polyesters. *J. Comput. Chem.* **15**, 752–768 (1994).

86. H. Sun, S. J. Mumby, J. R. Maple, A. T. Hagler, An ab initio CFF93 all-atom force field for polycarbonates. *J. Am. Chem. Soc.* **116**, 2978–2987 (1994).

87. H. Sun, Ab initio calculations and force field development for computer simulation of polysilanes. *Macromolecules* **28**, 701–712 (1995).

88. H. Sun, Ab initio characterizations of molecular structures, conformation energies, and hydrogen-bonding properties for polyurethane hard segments. *Macromolecules* **26**, 5924–5936 (1993).

89. H. Sun, S. Mumby, J. Maple, A. Hagler, Ab initio calculations on small molecule analogs of polycarbonates. *J. Phys. Chem.* **99**, 5873–5882 (1995).

90. O. Arnold, J.-C. Bilheux, J. Borreguero, A. Buts, S. I. Campbell, L. Chapon, M. Doucet, N. Draper, R. F. Leal, M. Gigg, Mantid—Data analysis and visualization package for neutron scattering and μ SR experiments. *Nucl. Instrum. Methods Phys. Res. A* **764**, 156–166 (2014).

91. E. Mamontov, R. W. Smith, J. J. Billings, A. J. Ramirez-Cuesta, Simple analytical model for fitting QENS data from liquids. *Phys. B Condens. Matter* **566**, 50–54 (2019).

92. K. S. Cole, R. H. Cole, Dispersion and absorption in dielectrics I. Alternating current characteristics. *J. Chem. Phys.* **9**, 341–351 (1941).

93. Z. Lu, T. Jiang, Z. Min, S. Ke, A review of the coefficient of thermal expansion and thermal conductivity of graphite. *New Carbon Mater.* **37**, 544–555 (2022).
